# Bis-3-chloropiperidines: a novel motif for anthelmintic drug design†

**DOI:** 10.1039/d4ra05699j

**Published:** 2025-01-10

**Authors:** Michael Kirchner, Michael Marner, Tim Kramer, Felix Mühlemeyer, Johanna Eichberg, Markus Oberpaul, Simone Haeberlein, Richard Göttlich

**Affiliations:** a Institute of Organic Chemistry, Justus Liebig University 35392 Giessen Germany Richard.goettlich@org.chemie.uni-giessen.de https://www.uni-giessen.de/de/fbz/fb08/Inst/organische-chemie/AGGoettlich; b Branch for Bioresources of the Fraunhofer Institute for Molecular Biology and Applied Ecology 35394 Giessen Germany; c Institute for Parasitology, Justus Liebig University 35392 Giessen Germany; d BMBF Junior Research Group in Infection Research „ASCRIBE”, Branch for Bioresources of the Fraunhofer Institute for Molecular Biology and Applied Ecology IME Ohlebergsweg 12 35392 Giessen Germany

## Abstract

Parasites account for huge economic losses by infecting agriculturally important plants and animals. Furthermore, morbidity and death caused by parasites affect a large part of the world population, especially in economically weak regions. Anthelmintic drugs to tackle this challenge remain scarce and their efficiency becomes increasingly endangered by the advent of drug resistance development. In the present study, we assessed the anthelmintic potential of bis-3-chloropiperidines, a family of compounds which have already demonstrated antiproliferative activity against various cell lines. We synthesized and tested the activity of 21 bis-3-chloropiperidine derivatives against two strains of the free-living nematode *Caenorhabditis elegans* (N2 and DC19) and the parasitic flatworm *Schistosoma mansoni*. Overall, bifunctional chloropiperidines featuring an aromatic linker performed best against the tested indicator organisms and could be considered for future optimization efforts. Ultimately, out of the 21 analyzed bis-3-chloropiperidines, four derivatives (2, 5, 9 and 11) reduced vitality parameters against *S. mansoni* and five the motility of *C. elegans* (2, 4, 5, 13, 21) while exhibiting no or low cytotoxicity.

## Abstract 2

We report the *in vitro* screening of bis-3-chloropiperidine derivatives against the free-living *Caenorhabditis elegans* and the parasitic *Schistosoma mansoni*. 56% of compounds active against *C. elegans* were also active against *S. mansoni*.

## Graphical abstract

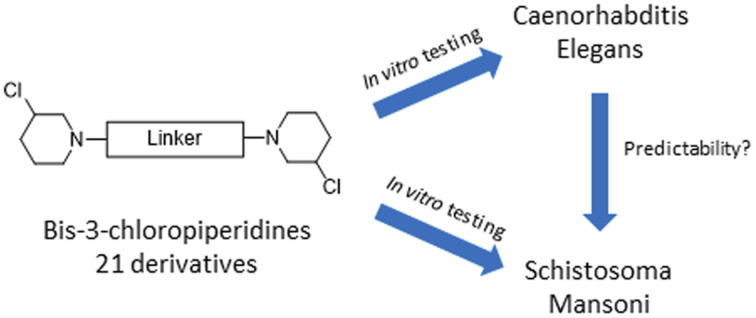

## 1 Introduction

Schistosomiasis and soil-transmitted helminthiases are among the most common infectious diseases and pose a major challenge in veterinary and human medicine. The World Health Organization (WHO) estimated that 1.5 billion people are infected by soil-transmitted helminths such as *Ascaris lumbricoides*, *Trichuris trichiura* or *Necator americanus*.^1^ At the same time, helminths like *Haemonchus contortus* cause immense economic losses in livestock by infecting in particular small ruminants like sheep, goats and young cattle.^2^

In 2022, more than 0.25 billion people required preventive chemotherapy, while several millions suffered from severe morbidity as a consequence of infections with the blood fluke(s) *Schistosoma* sp. Since the introduction of praziquantel (PZQ) in the 1980s, no alternative treatment option for schistosomiasis was developed. Hence, besides vector control and the improvement of sanitation and water safety, strategies to fight neglected tropical diseases (NTD) in low- and middle-income countries (LMIC) are the development of multiplex diagnostics accompanied with new treatment approaches.^3^ The situation is further aggravated by anthelmintic resistance (AR) to the already limited number of efficacious drugs. For each major anthelminthic compound class, widespread resistance emerged within parasitic nematodes.^4,5^ Likewise, reduced efficacy of the only antischistosomal drug PZQ was reported.^6^ Thus, research and development towards reproposed or new anti-infective agents is urgently needed.^7–10^ This urgency has already been manifested in the emergence of some promising lead scaffolds including organic peroxides^11^ and avermectins.^12^

Many licenced anthelmintic small-molecules exhibit several biological activities including antimicrobial and anti-tumour.^13^ Hence, in a first step of anthelmintic drug discovery, the activity profile of a candidate compound is optimized to balance potential conflicting attributes such as parasite efficacy and *in vitro* cytotoxicity.^14^

In this study we set out to investigate the anthelmintic potency of a series of bis-3-chloropiperidines analogs inspired by natural product antibiotic 593A. Initially, Antibiotic 593A was isolated from *Streptomyces griseoluteus* in 1970 by Gittermann^15^ followed by total synthesis by Fukuyama^16^ in 1980. Besides the antibacterial activity, 593A proved to be active against solid tumors and leukemia, most importantly against cancer cells that were resistant against the commonly used antiproliferative drug cyclophosphamide.^17^ Interestingly, our simplified compounds as well showed activity in various cell-based assays.^18–20^ It was demonstrated that *bis*-3-chloropiperidines form an electrophilic and highly reactive aziridinium ion, which can be attacked by nucleophilic agents such as nucleobases or intracellular thiols.^21,22^

**Fig. 1:**
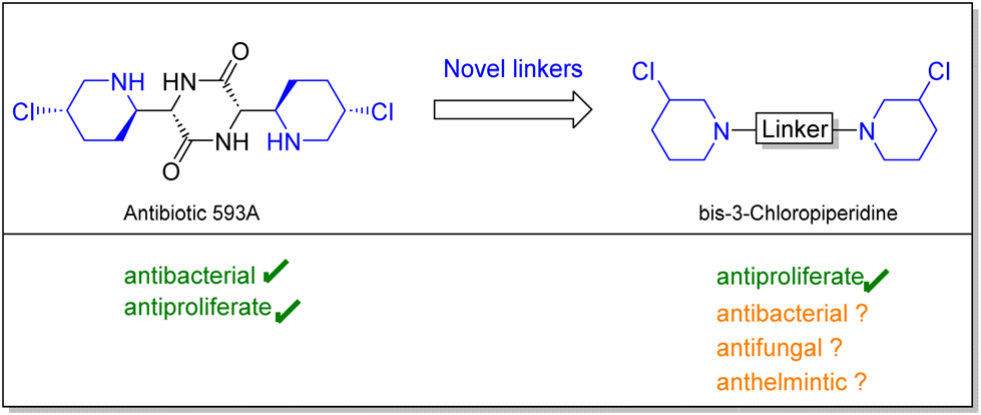
Preparation of simplified analogs of antibiotic 593A could yield novel pharmacologically active compounds.

Compared to clinically used nitrogen mustards, the reactive moiety is congested in a ring system, which could enhance DNA interactions *in vivo*.^23^ Moreover, Sosic *et al.* could show that next to DNA-alkylation, bis-3-chloropiperidines have a second mode of action and serve as an inhibitor of the human topoisomerase II α.^24^ Through previous studies, we could also find that aromatic and l-lysine linkers can significantly increase the activity of bis-3-chloropiperidines *in vitro*.^18,19,25^ Also geminal dimethylation proved to be a valid strategy to increase the activity of these compounds *in vitro*.^20^ Despite these intriguing findings, this series of compounds was, to the best of our knowledge, never screened for further applications (Fig. 1). Hence, in this study, we focused our research efforts to investigate whether these compounds might represent a starting point for an antimicrobial or anthelmintic drug design. First, we investigated the bioactivity of mono-, bi- and trifunctional 3-chloropiperidine agents in antibacterial and antifungal assays, followed by cytotoxicity evaluation in mammalian cell cultures. To analyse anthelmintic activity, we used the free-living nematode *Caenorhabditis elegans* (*C. elegans*) as a surrogate for the parasitic nematodes. Besides the wildtype *C. elegans* N2, we used strain DC19 in our assays. *C. elegans* DC19 combines an enhanced cuticle permeability with low fitness consequences. It is thus regarded as a drug sensitive screening strain with enhanced drug target accessibility. Out of our results we deduced an anthelmintic structure–activity relationship of 3-chloropiperidines and investigated the predictive value of *C. elegans* models for *Schistosoma mansoni* activity.

## 2 Results and discussion

### 2.1 Chemistry

**Fig. 2:**
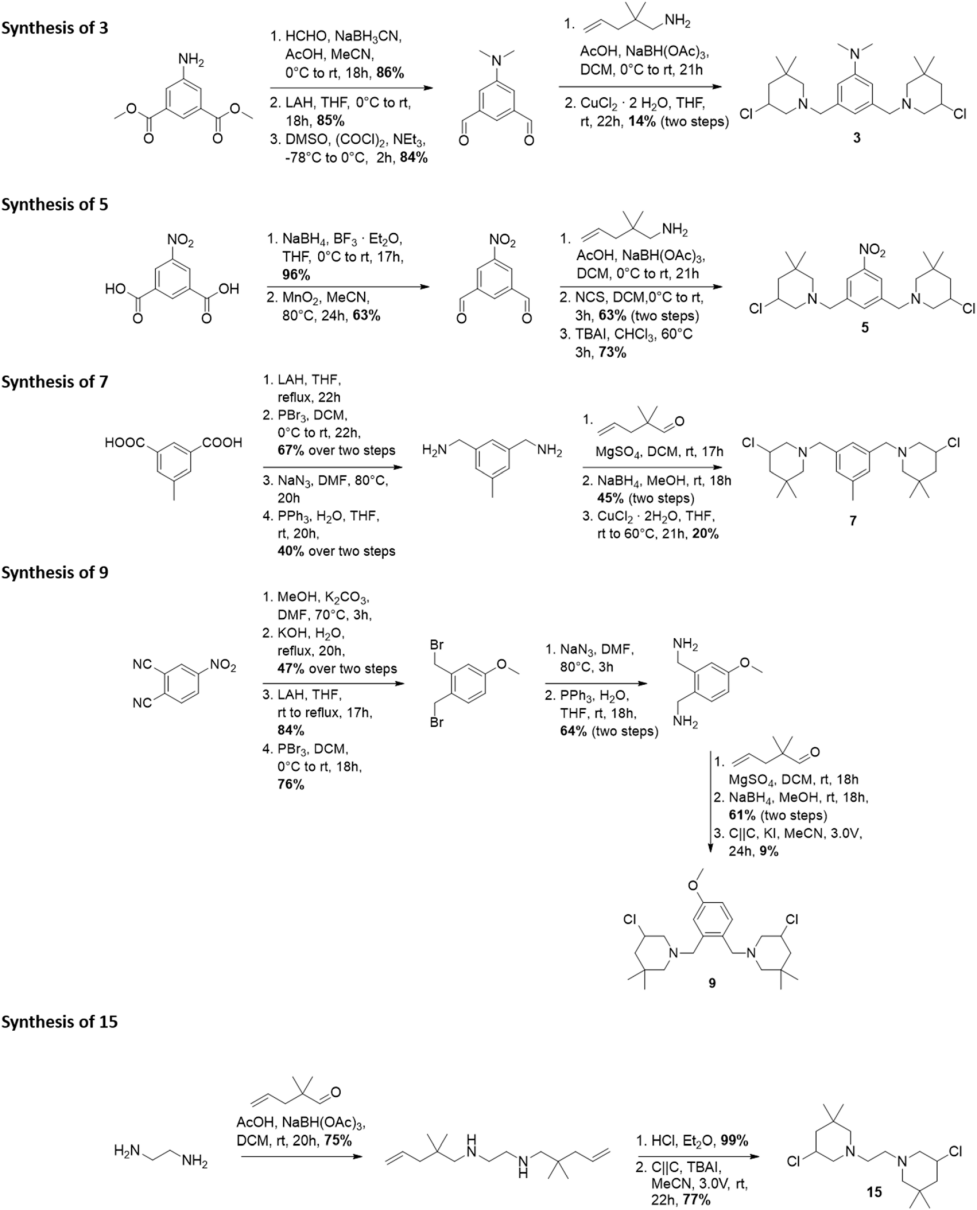
Synthesis of novel bis-3-chloropiperidines 3, 5, 7, 9 and 15.

**Fig. 3:**
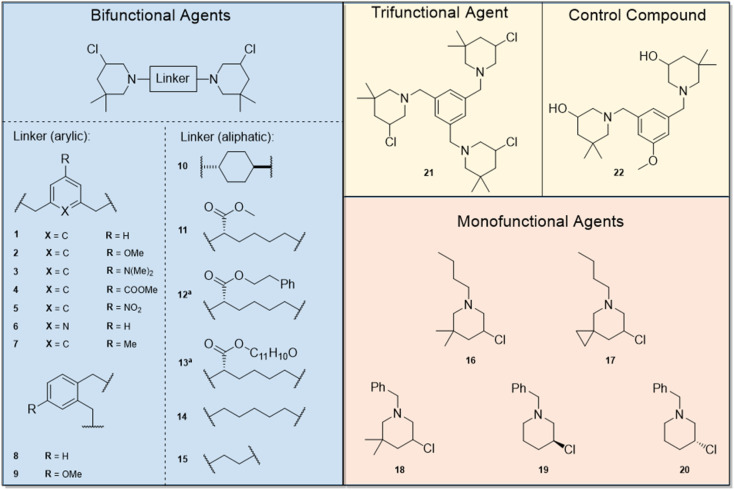
Synthesized mono-, bi and trifunctional 3-chloropiperidine agents. ^*a*^aliphatic linker with aromatic side-chain.

The synthesis of 3-chloropiperidines 1, 2, 4, 6, 8, 10–14 and 16–21 (Fig. 3) has been described in recent literature.^19,20,23,25–27^ This leaves compounds 3, 5, 7, 9 and 15 as novel compounds the synthesis of which we will describe hereinafter (Fig. 2). Compound 3 was prepared in a five-step sequence from commercially available dimethyl-5-aminoisophthalate. In the first step, two methyl groups were introduced *via* reductive amination in a yield of 86%. The respective aldehyde was then prepared by initial reduction with lithium aluminium hydride (LAH) with subsequent *Swern*-oxidation, which was accomplished in yields of 85% and 84% respectively. The 3-chloropiperidine moiety was then formed through reductive amination with 2,2-dimethyl-pent-4-enylamine and consecutive *in situ* chlorination/cyclization with copper(ii)–chloride, following a procedure of Liu^27^*et al.* The final product 3 was thereby obtained in a yield of 14% over two steps. Formation of the 3-chloropiperidine moiety by initial *N*-chlorination of the secondary amine with *N*-chlorosuccinimide (NCS) and successive cyclization with tetrabutylammonium iodide (TBAI)^28^ did not yield any product, presumably due to oxidation of the reactive dimethyl aniline moiety.

Compound 5 was prepared from 5-nitroisophthalic acid. The carbonic acid was first selectively reduced with NaBH_4_ to the alcohol under activation with BF_3_·Et_2_O in a yield of 96%. The obtained alcohol was readily reoxidized to the aldehyde by activated MnO_2_ in acetonitrile in a yield of 63%. The secondary amine was then formed by reductive amination with 2,2-dimethylpent-4-enylamine and could be readily oxidized by NCS without further purification. Afterwards, catalytic cyclization with TBAI^28^ provided 5 in a yield of 73%. The synthesis of 7 was attempted starting from 5-methylisophtalic acid. Thereby, the acid was reduced to the respective alcohol with LAH. The alcohol was then readily brominated with PBr_3_ in a yield of 67% over two steps. Then, the azide was formed by nucleophilic substitution using sodium azide, which was followed by Staudinger reduction to yield the respective amine in a yield of 40% over two steps. Step-wise reductive amination with 2,2-dimethylpent-4-enal was then leveraged to avoid multiple substitutions on one nitrogen center. This gave a yield of 45% of the respective secondary amine. This compound could again be cyclized with copper(ii) chloride in THF^27^ in a yield of 20%. Electroorganic cyclization^29^ did not lead to any conversion, presumably as the respective hydrochloride was insufficiently soluble in acetonitrile.

Compound 9 was prepared in a nine-step sequence from commercially available 4-nitrophthalonitrile. In the first step, the nitro-group was replaced with a methoxy-group by nucleophilic aromatic substitution. As the direct reduction to the diamine with LAH or Red-Al resulted merely in partial reduction, we introduced the amine functionality over a step-wise way. Thereby, the crude residue was first subjected to alkaline hydrolysis which yielded the respective carbonic acid in a yield of 47% over two steps. Reduction with LAH afforded the respective alcohol in 84% yield. Consecutive bromination with PBr_3_ afforded the intermediate bromide in a yield of 76%. The obtained bromide could now be converted to the respective amine by nucleophilic substitution and consecutive *Staudinger* reduction, which resulted in a yield of 64%. Imine formation with 2,2-dimethylpent-4-enal and reduction again afforded the secondary amine in a good yield of 61%, which could then be precipitated quantitatively as the hydrochloride salt with ethereal hydrochloric acid. The hydrochloric acid salt could then be cyclized under electroorganic conditions^29^ to yield compound 9 with a yield of 9%.

Compound 15 was prepared from commercially available ethylene diamine, which was first reductively aminated with 2,2-dimethylpent-4-enal to yield 75% of the corresponding secondary amine. The free amine was then precipitated quantitatively as the hydrochloride salt with ethereal hydrochloric acid. The hydrochloride salt was then cyclized in an electroorganic,^29^ metal-free fashion to form 15 in a yield of 77%. For synthetic details, the reader is referred to the ESI.† With these novel compounds at hand, we tested the antimicrobial and anthelmintic activity.

### 2.2 Antimicrobial and anthelmintic activity tests

The minimum inhibitory concentration (MIC) of the synthesized compounds was initially determined against three microbial indicator strains (*Escherichia coli* ATCC35218, *Streptococcus aureus* ATCC33592, *Septoria tritici* MUCL45408).

Only compounds 8 and 13 showed weak activity against *S. tritici* (64–32 and 16 μg mL^−1^ respectively), while the remaining derivatives were inactive (64 or >64 μg mL^−1^) against the selected indicator strain at the tested concentrations.

To evaluate the anthelmintic potential of compounds 1–22, we chose the nematode *C. elegans* as a model system for parasitic nematodes. In addition, we screened the compound series against the obligate internal parasitic trematode *S. mansoni* and determined cytotoxicity against the canine kidney cell line (MDCK II, Table 1) at a high dose of 100 μM and subsequently determined the CC_50_ for prioritized compounds (Table 2).

**Table 1:** Screening results of the activity tests against *E. coli* (Ec), *S. aureus*, *S. tritici* (St), *C. elegans* (N2 and DC19) and a canine kidney cell line (MDCK II). Positive controls were chosen according to the nature of the test strain. For bacteria: rifampicin (RIF), tetracycline (TET), gentamicin (GEN). For St: tebuconazole (TEB), amphotericin B (AMP), nystatin (NYS). *C. elegans*: ivermectin (IVR) and MDCKII cells: ionomycin (ION). MIC: minimum inhibitory concentration given in μg mL; MMIC: minimum motility inhibitory concentration given in μg mL^−1^. Cytotoxicity at high dose of 100 μM against MDCKII either “+” (toxic) or “−” (not toxic). Compounds that resulted in a cell viability of less than 80% relative to the DMSO control were categorized as cytotoxic. nd: not determined

	MIC [μg mL^−1^]			MMIC [μg mL^−1^]		MDCKII
ID	Ec	Sa	St	N2	DC19	
1	>64	>64	64	64	64–32	+
2	>64	>64	64	16–8	16–8	−
3	>64	>64	>64	16	32–16	+
4	>64	>64	>64	16	16	−
5	>64	>64	>64	16	16	−
6	>64	64	>64	64	64–32	+
7	>64	>64	>64	32–16	16	+
8	>64	>64	64–32	8	8–4	+
9	>64	>64	>64	>64^a^	64	+
10	>64	>64	>64	>64	>64	+
11	>64	>64	>64	>64^a^	>64	−
12	>64	>64	>64	16–8	16	+
13	>64	>64	16	32–16	8–4	−
14	>64	>64	>64	>64	64	+
15	>64	>64	>64	>64	>64	+
16	>64	>64	>64	>64	>64	−
17	>64	>64	>64	>64	>64	−
18	>64	>64	>64	>64	32	+
19	>64	>64	>64	>64	>64	−
20	>64	>64	>64	>64	>64	−
21	>64	>64	>64	16–8	16–8	−
22	>64	>64	>64	>64	>64	−
RIF	4	>64	nd	nd	nd	nd
TET	4–2	32	nd	nd	nd	nd
GEN	1	0.5	nd	nd	nd	nd
TEB	nd	nd	>0.03	nd	nd	nd
AMP	nd	nd	0.125	nd	nd	nd
NYS	nd	nd	0.25	nd	nd	nd
IVR	nd	nd	nd	0.005	0.005	nd
ION	nd	nd	nd	nd	nd	+

a Some worms were underdeveloped at 64–32 μM.

**Table 2:** Summary of prioritized compounds and their effects on tested nematodes and MDCKII cells. Compounds exhibiting moderate effect on *C. elegans* strains N2 and DC19 (MMIC ≤20 μg mL^−1^) and/or inflicting phenotypic changes in *S. mansoni* assays, while not showing reduced MDCKII cell viability at a high dose of 100 μM were subject of CC_50_ determination against the same cell line. MMIC: minimum motility inhibitory concentration. Det: detachment of suckers, Mot: motility reduction, Sep: Pair separation. Compare Table 1 and Fig. 4

ID	MMIC [μg mL^−1^] N2 and DC19	Effect on *S. mansoni*	MDCKII CC_50_ [μM]
2	16–8	Det, Sep, Mot, Lethal	83
4	16	—	205
5	16	Det, Sep, Lethal	>1000
9		Det, Sep, Lethal	70
11		Det, Sep	123
13	8–4	—	>1000
21	16–8	—	>1000

The cuticle of *C. elegans* strain DC19 is known to be more permeable for small molecules compared to the wildtype N2.^30^ DC19 is thereby considered an informative drug-sensitive test organism. In our assays, we determined the minimum mobility inhibitory concentration (MMIC) as a proxy for anthelmintic activity. For most of our 3-chloropiperidines derivatives however, the susceptibility of the two *C. elegans* stains was similar, indicating that the moderate activity is not influenced by the cuticle structure. In contrast, MMICs of some small molecule anthelmintics such as albendazole, mebendazole or PF-1022A were lower for DC19 (Fig. S1†).

In general, we observed that mono-functional agents (16–20) did not have any effect on the test organisms (MIC and MMIC >64 μg mL^−1^) and canine kidney cells, while compounds featuring two or three 3-chloropiperdine building blocks showed a range of activities.

Inactivation of the reactive moiety, by exchange of the chlorine atom in the piperidine scaffold with a hydroxyl group, eliminated the *C. elegans* mobility inhibition (comparison 2 and 22). This suggests, that the observed activity of *e.g.* of compound 2 is indeed related to the previously mentioned reactive aziridinium ion formation, a reaction requiring the electrophilic β-carbon.

The results of the bifunctional 3-chloropiperidines indicate a strong influence of the linker moiety on the bioactivity against our surrogate models N2 and DC19 (Table 1). Derivatives with an aliphatic linker such as a cyclohexane moiety (10) or a linear, aliphatic chain (14 and 15) seem to have weak activity (MMIC 64 μg mL^−1^ and above), while substitution of the chain with aromatic ester groups increased potency to 16 μg mL^−1^ (12) and 8–4 μg mL^−1^ (13) against DC19. This effect was not observed for the methyl ester derivative 11. Compounds featuring an aromatic linker performed better against *C. elegans*. However, the data indicate, that the substitution pattern of the employed aromatic linker is crucial for activity. Switching from a meta-substituted aromatic system to an *ortho*-substituted aromatic compound lowered the MMIC from 64 μg mL^−1^ (1) to 8 μg mL^−1^ (8). Furthermore, substitution of the benzylic linker with a pyridinylic linker (6) also resulted in activity.

By comparison of compound 1 with 2–5, we concluded that a second meta-substitution of the linker might be beneficial for activity. We observed that a linker featuring electron withdrawing or electron donating substituents increases the anti-nematode activity of the compounds (compounds 2–5). Comparison of MMICs of compound 1, 2 and compound 7, a bis-3-chloropiperidine featuring a 5-methylbenzene linker with intermediate electron density compared to 1 and 2, supports this hypothesis (1 = 64–32 μg mL; 7 = 32–16 μg mL^−1^ and 2 = 16–8 μg mL^−1^). A compound with a third 3-chloropiperidine moiety (21) in meta-position exhibits the same degree of activity as 2 with only two active moieties (16–8 μg mL^−1^).

**Fig. 4:**
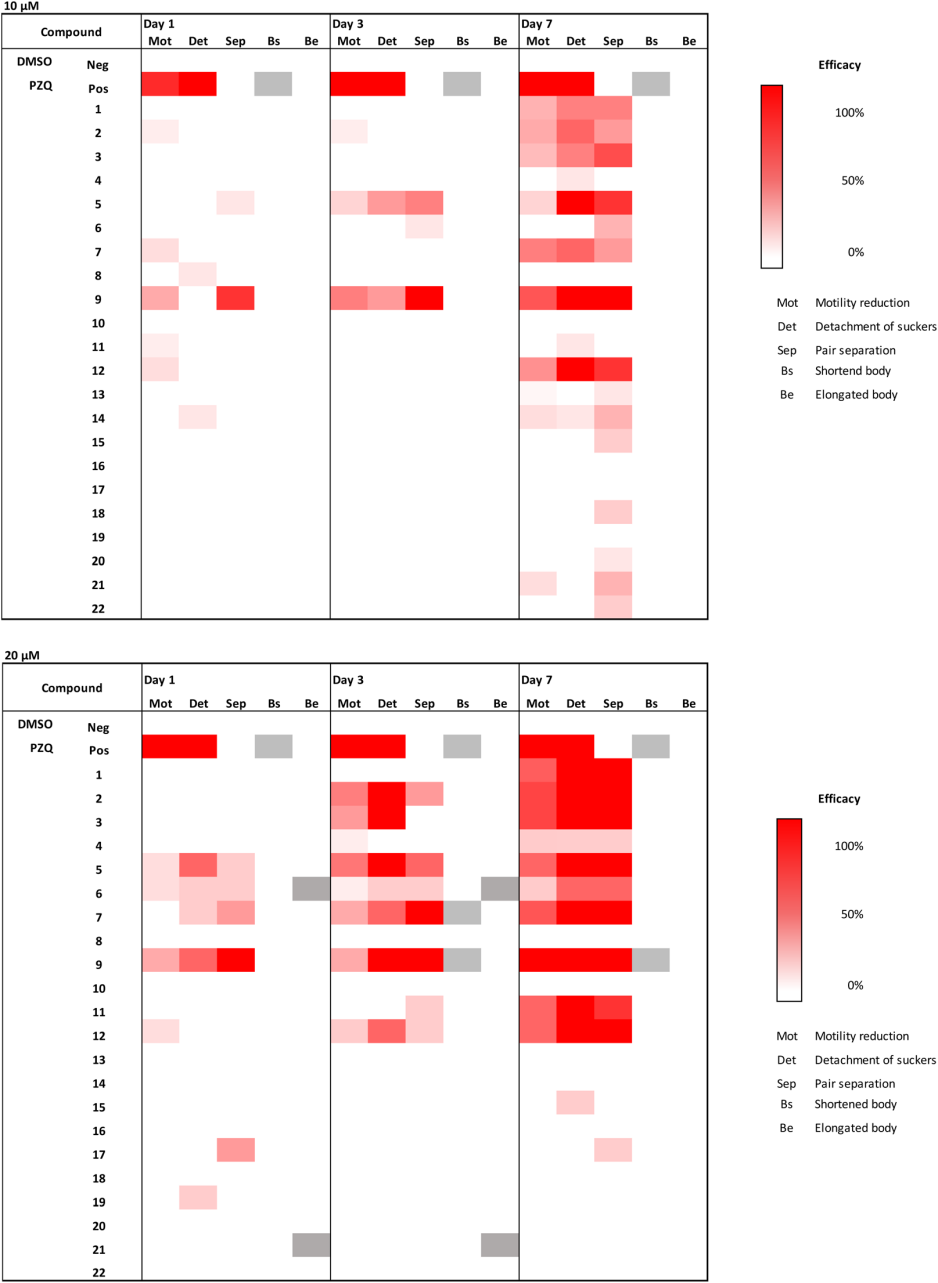
Activity of 3-chloropiperidine derivatives against *S. mansoni* at a concentration of 10 μM and 20 μM. Phenotypic readout (Mot: motility reduction, Det: detachment of suckers, Sep: pair separation, Bs: shortened body, Be: elongated body) was done after 1, 3 and 7 days. Efficacy is expressed weak (white) and strong (red). Positive control (Pos): *Praziquantel* PZQ. Hatched: not determined. Mean values of two independent experiments are shown.

Compound 9 combines the structural elements of 2 and 8, which both showed moderate MMICs in our initial tests. However, the combination of an ortho-substituted aromatic linker (like 8) substituted with an additional methoxy group (like 2) did exert comparable activity against N2 or DC19 (64 μg mL^−1^ compared to 16–8 and 8–4 μg mL^−1^ respectively). Overall, compounds 2–5, 7–8, 12–13 and 21 were observed to exhibit the strongest *C. elegans* mobility inhibitory activity of <20 μg mL^−1^. Of these, compounds 2, 13 and 21 did not exhibit cytotoxic properties. Subsequently, we tested whether compounds with nematocidal activity display a broader activity also against parasitic flatworms. To this end, *in vitro* tests against the blood fluke *S. mansoni* were conducted (Fig. 4). The worms were incubated in presence of 10 and 20 μM of compounds 1–22 and phenotypic vitality parameters (separation of worm pairs, detachment of suckers from the bottom of the well and weakening of body movements) were assessed as previously described.^31^ Compounds reaching 100% effect strength in one of the parameters within 7 days were considered active. Reduction of motility is considered most relevant for antischistosomal drug candidates.^32^ In addition, loss of sucker activity would make parasites drifting off from their host habitat (mesenteric veins) and with pair separation, the production of pathology-causing eggs eventually ceases.^33^ From the herein investigated 3-chloropiperidines, 56% of *C. elegans* active derivatives (MMIC ≤20 μg mL^−1^) also showed an effect on *S. mansoni* (compounds 2, 3, 5, 7, 12) whereas four compounds (4, 8, 13 and 21) were *C. elegans*- and three (1, 9, 11) *Schistosoma*-specific. For some molecular targets *e.g.* glutamate and GABA chloride channels (ivermecin, abamectin) and β-tubulin (albendazole, mebendazole) the *C. elegans* assays results were transferable to *Schistosoma*, while for others *e.g.* acetylcholin mimetic (levamisole) the *C. elegans* MMIC was not predicative (Fig. S1†).

Overall, none of the compounds caused 100% lethality at the tested concentrations, which means their antischistosomal activity can be considered rather weak. However, at 20 μM, nine compounds were found active while at 10 μM, activity remained only for three. At 10 μM, only 5 and 12 led to full sucker detachment of *Schistosoma* worm pairs, while the other parameters remained unaltered by the treatment (Fig. 4). Compound 9 led to the separation of all worm pairs after 3 days and to a lesser extent already at day 1 at 10 μM.

Looking at effects in more detail, we did not observe any changes in the vitality parameters of *Schistosoma* as a response to monofunctional 3-chloropiperidines exposure, which is in agreement with our *C. elegans* data. While the trifunctional derivative (21) exited mobility inhibitory activity in N2 and DC19, both did not affect *Schistosoma*.

Similarly, the bifunctional compounds 8 and 13, which showed the strongest mobility inhibition against *C. elegans* DC19 (8–4 μg mL^−1^*e.g.*, 17–8.5 μM and 12.5–6.3 μM respectively), did not inflict any phenotypic changes in *Schistosoma* at 20 μM in comparison to the untreated control.

Despite that, exposure to bifunctional 3-chloropiperidines with an aromatic linker strongly affected vitality parameters in *Schistosoma* at 20 μM. We observed that the efficacy of these compounds increased over time. In that sense, we observed detachment of suckers and pair separation of all worm pairs treated with compounds 1–3, 5, 7, 9, 11 and 12 at 20 μM (Fig. 4) after 7 days incubation. Most strikingly, exposure to compounds 2, 3 and 9 led to sucker detachment already after three days and lethal effects after seven days. This indicates that the activity of the 3-chloropiperidines against *S. mansoni* might be correlated with the electron density in the aromatic system, as compound 4 with a low electron density in the aromatic system exerted no activity against *S. mansoni*. On the other hand, the strong activity of nitro-substituted system 5 surprised us, as it can be considered active at 10 μM, even though the implemented aromatic system is very electron-poor. However, the CC_50_ for 5 (Table 2) was significantly higher than for 2, 9 and 11, which could indicate that they have a different mode of action. Overall, three compounds (1, 5, 12) stopped motility almost completely (motility score 1–1.2). Fast action of compounds is an important parameter for defining antischistosomal activity, considering a rather short exposure of parasites to initial drug concentrations within the mesenteric veins.^34^ After a short treatment period of one day, only methoxylated compound 9 was active, causing complete worm pair separation (as observed at 10 μM). This indicates that the position of the methoxy-group on the arylic system might be crucial for good activity. Therefore, it could prove valuable to investigate other arylic substitution patterns for their biological activity (such as the two possible 1,2,3-substitution patterns). As the three methoxylated 3-chloropiperidines 2, 9 and 11 were all active at 20 μM, a correlation between activity and the count of methoxy groups might also be likely.

The cytotoxicity assessment revealed that from the nine compounds, which were seen to reduce vitality parameters of *S. mansoni*, five (1, 3, 7, 9 and 12, see Table 1 and Fig. 4) were cytotoxic at 100 μM. However, due to the fast action of compound 9 at 10 μM, we additionally tested a triplicated dilution series of this compound against MDCK-II. Based on the dose–response curve (Fig. S3†) a CC_50_ value of 70 μM was determined, indicating a 7-fold stronger effect on *Schistosoma* pair separation as on cell viability reduction (Table 2 and Fig. S3†). Compounds 2 (lethal effect at 20 μM), 5 (sucker detachment at 10 μM), 11 (detachment of suckers and pair separation at 20 μM) showed even weaker cytotoxic effects (CC_50_ of 83 μM (2), >1000 μM (5) and 123 μM (11)), leaving them, in addition to compound 9, as potential starting points for anti-*Schistosoma* drug design.

## 3 Conclusions

This study highlighted the anthelmintic potential of bis-3-chloropiperidines. Herein, we demonstrated that the activity against *C. elegans* and *S. mansoni* is very dependent on the employed linker system. Generally, bifunctional compounds featuring an aromatic linker performed better. Further studies towards the molecular target(s) and mode of action in nematodes should be conducted to understand the true value of the presented group of compounds with regard to anthelmintic drug design. Despite that, we could show that the transferability from the *C. elegans* to *Schistosoma* assays is generally good (56%). However, we also observed a high degree of *Schistosoma* specificity (33%). Ultimately, we could find nine compounds that reduced one or more vitality parameters of the parasitic flatworm *S. mansoni* of which compounds 2, 5, 9 and 11 are balancing cytotoxicity and anti-parasitic activity the best.

## Author contributions

M. Kirchner performed the synthesis and analytics and prepared the draft. M. Marner performed the biological studies and prepared the draft. T. Kramer assisted with the synthesis and analytics. F. Mühlemeyer, J. Eichberg and M. Oberpaul assisted with the biological studies. S. Haeberlein assisted in preparing the draft and with the biological studies. R. Göttlich assisted in preparing the draft and administered the project.

## Conflicts of interest

The authors declare no conflict of interest.

## Supplementary Material

RA-015-D4RA05699J-s001
