# Supplementary material for: Bis-3-chloropiperidines: a novel motif for anthelmintic drug design
Source: RSC Adv. 2025 Jan 10;15(2):824–31. doi: 10.1039/d4ra05699j (PMC11718441; doi:10.1039/d4ra05699j)
Supplement: RA-015-D4RA05699J-s001 [file RA-015-D4RA05699J-s001.pdf]

Supplementary Information (SI) for RSC Advances.
This journal is © The Royal Society of Chemistry 2025

Supporting information

Bis-3-chloropiperidines: A novel motif for anthelmintic drug design

M. Kirchner[a,†], M. Marner[b,†], T. Kramer[a], F. Mühlemeyer[b], J. Eichberg[b,d], M. Oberpaul[b,d], S. Haeberlein[c] and R. Göttlich[*a]

[a]Institute for organic chemistry, Justus Liebig University, 35390 Giessen, Germany.

[b]Branch for bioresources of the Fraunhofer Institute for molecular biology and applied ecology, 35394 Giessen, Germany.

[c]Institute of Parasitology, Justus Liebig University, 35392 Giessen, Germany.

[d] BMBF Junior Research Group in Infection Research „ASCRIBE", Branch for Bioresources of the Fraunhofer Institute for Molecular Biology and Applied Ecology IME, Ohlebergsweg 12, 35392 Giessen, Germany.

† The authors contributed equally

Table of contents

1. General considerations....3
2. Microbial and parasitological studies....3
   2.1 Microbial studies....3
   2.2 *In vitro* tests against *Caenorhabditis Elegans*....4
   2.2 Parasitological studies....5
   2.2.1. Ethical statement....5
   2.2.2. Parasites....5
   2.2.3. *In vitro* tests against *Schistosoma mansoni*....5
   2.3. In vitro cytotoxicity assays....7
   2.3.1. Material and Methods....7
   2.3.2. Results....7
3. Experimental procedures....10
   3.1 General remarks....10
   3.2 Synthesis of compound 3....10
   3.3 Synthesis of compound 5....14

3.4 Synthesis of compound 7 17
3.5 Synthesis of compound 9 20
3.6 Synthesis of compound 15 24
3.7 Synthesis of control compound 22 25
4. Copies of spectra 26
Compound 3A 26
Compound 3B 27
Compound 3C 28
Compound 3D 29
Compound 3 30
Compound 5A 31
Compound 5B 32
Compound 5C 33
Compound 5D 34
Compound 5 35
Compound 7A 36
Compound 7B 37
Compound 7C 38
Compound 7 39
Compound 9A 40
Compound 9B 41
Compound 9C 42
Compound 9D 43
Compound 9E 44
Compound 9 45
Compound 15A 46
Compound 15 47
Compound 22 48
5. References 50
6. Author contribution 51

1. General considerations

Commercially available solvents were purified prior to use. For anhydrous solvents, AcroSeal™ bottles from ACROS Organics™ were used. Other commercially available chemicals were used as obtained from the supplying company unless otherwise noted. Synthetic procedures under anhydrous conditions were performed applying standard *Schlenk* technique. For purification by column chromatography, silica gel 60 (supplied by *Merck*) was used. ^{1}H and ^{13}C NMR spectra were recorded at the *Bruker Avance II 400* and *Bruker Avance III 400* spectrometers in deuterated solvents. High-resolution mass spectra were recorded in methanol with an *ESImicroTOF* spectrometer from *Bruker Daltonics* in positive ion mode. For electroorganic synthesis a *Sky Toppower PS1110* was used as a power supply. Electrodes were sonicated in acetone and abrased with sand paper (120 grid, then 180 grid) prior to usage.

2. Microbial and parasitological studies

2.1 Microbial studies

The minimum inhibitory concentration (MIC) of the synthesized compounds was determined against three microbial indicator strains (*E. coli* ATCC35218, *S. aureus* ATCC33592 and *S. tritici* MUCL45408) as previously described.[1,2] All samples were dissolved in DMSO and tested in triplicate. Briefly, a pre-culture of *E. coli* and *S. aureus* was prepared by incubating the strains in cation adjusted Mueller Hinton 2 broth (Becton Dickinson, Sparks, NV, USA) at 37°C and 180 rpm overnight. The cell density of adjusted to 5×10^5 cells/mL before assays were incubated at 37 °C, 180 rpm and 85% rH. Three distinct dilution series of standard antibiotics (gentamicin, rifampicin, and tetracycline) were used as positive controls on each assay plate. Inoculated medium without test compound or positive control was used as negative control. Medium background was averaged from 5 replicates. The turbidity of each well was measured with a microplate spectrophotometer at 600 nm (LUMIstar® Omega, BMG Labtech) and growth inhibition was calculated relative to the absorption of the controls. For *S. tritici* MUCL45408 inoculated at a density of 1×10^5 cells/mL in potato

dextrose medium. *Septoria* assay plates were incubated for 4 days at 25 °C, 180 rpm and 85% rH. Tebuconazole, amphotericin B, and nystatin were used as positive controls. Cell viability was evaluated via ATP quantification (BacTiter-Glo™, Promega GmbH, Walldorf, Germany) according to the manufacturer's instructions.

2.2 *In vitro* tests against *Caenorhabditis Elegans*

The minimal motility inhibitory concentration (MMIC) of compounds **1-22** against *C. elegans* was determined as described before.[3] Both strains, *C. elegans* N2 and DC19 were treated identically. Briefly, pre-cultures of *C.elegans* were kept on NGM agar plates infused with auxotrophic *E.coli* OP50 as food source.[4] After 4 days the plates contained sufficient gravid worms, respectively. Worms were washed from the plates with M9 buffer using wide bore pipette tips. Egg preparation was carried out by treating the harvested worms with an alkaline hypochlorite solution (5 M NaOH + 5 % NaOCl 1:2) in polypropylene tubes. Larvae and adult worms do not tolerate this treatment, while eggs survive. We carried out 3 washing steps (M9 buffer) to remove carry-over hypochlorite solution. Subsequently, the eggs were incubated overnight in NGM broth on a rotator to yield synchronized hatched L1/L2 larvae. The larvae solution was adjusted to 100 worms/mL and supplemented with cholesterol (5 µg/mL), carbenicillin (25 µg/mL) and *E.coli* OP50 (0.5%). This assay solution was distributed into 96 well plates and incubated with a dilution series of compounds **1-22** (64-0.5 µg/mL). An additional eight-point dilution series of ivermecin (40 – 0.3 ng/mL) was used as positive control on each assay plate. Solvent background was controlled by supplementation of pure DMSO without any compound. Assays were incubated for 2 days at room temperature. Finally, a cell imaging multimode reader (Cytation™ 5) was used to record short videos (1.5 seconds) of each individual test well. The videos were used to evaluate the worm motility.

2.2 Parasitological studies

2.2.1. Ethical statement

Animal experiments were performed in accordance with Directive 2010/63/EU on the protection of animals used for scientific purposes and the German Animal Welfare Act. The experiments were approved by the Regional Council (Regierungspraesidium) Giessen (V54 – 19c 20 15 h 02 Gi 18/10 Nr. A 26/2018).

2.2.2. Parasites

A Liberian strain of *S. mansoni* was maintained in the freshwater snail *Biomphalaria glabrata* as intermediate host and Syrian hamsters (*Mesocricetus auratus*) as final host (Janvier Labs, France).[5,6] Hamsters were infected at 8 weeks of age with 1,750 cercariae by using the paddling method.[7] Adult worms were collected at 46 days p.i. by hepatoportal perfusion and cultured in M199 medium (Sigma-Aldrich, Germany) supplemented with 10% Newborn Calf Serum (NCS, Sigma-Aldrich), 1% 1 M HEPES (ThermoFisher Scientific, USA) and 1% ABAM solution (10,000 units/ml penicillin, 10 mg/ml streptomycin and 25 mg/ml amphotericin B; Sigma-Aldrich, Germany) at 37 °C, 5% CO_2 and humidified atmosphere.

2.2.3. *In vitro* tests against *Schistosoma mansoni*

Test compounds were added at 10 µM or 20 µM in supplemented M199 medium to schistosomes (5 worm pairs per replicate) followed by a 7 d *in vitro* culture. Worms receiving the solvent DMSO at equal dilution served as negative control. The following vitality parameters were assessed every 24 h by bright field microscopy using a Leica DM IL inverted microscope and x2.5 objective lens: separation of worm pairs into male and female worms; detachment of suckers from the bottom; and weakening of body movements. Worm motility was scored as described previously, [8] with the scores 3 (normal motility), 2 (reduced motility), 1 (minimal and sporadic movements), 0 (no movements within 20 sec; dead worms). An average score of all worms per well was calculated to obtain a motility score. An average motility score of 0, separation of all pairs or detachment of all worms per well was considered as 100% effect strength.

		S.mansonii												*Celegans*	
		**Day 1**				**Day 3**				**Day 7**				MMIC [µg/mL]	
	**Compound**	**Mot**	**Det**	**Sep**	**Sho**	**Mot**	**Det**	**Sep**	**Sho**	**Mot**	**Det**	**Sep**	**Sho**	**N2**	**DC19**
Neg	DMSO														
Pos	Praziquantel													>10	>10
	Nitazoxanide													>10	>10
	Aldicarb													>10	>10
	Levamisole													0.3	0.3
	Abamectin													< 0.08	< 0.08
	PF-1022A													10	2.5
	Albendazole													0.16	< 0.08
	Mebendazole													1.25	0.3
	Ivermectin													0.005	0.005

Figure S 1. Activity common small molecule anthelminthics.

2.3. In vitro cytotoxicity assays

2.3.1. Material and Methods

MDCK II cells, kindly provided by Eva Böttcher-Friebertshäuser from the University of Marburg, were cultured in Dulbecco's Modified Eagle Medium (DMEM GlutaMAX) supplemented with 10% fetal calf serum and 1% penicillin/streptomycin (all materials from ThermoFisher Scientific, Waltham, MA, USA). The cells were maintained in a standard incubator at 37 °C in a 5% CO_2 atmosphere. The compounds of interest, along with ionomycin (obtained from Cayman Chemical, Ann Arbor, MI, USA) as a positive control for cytotoxicity, were dissolved in DMSO to create stocks of 10 mM and were stored at -20°C.

MDCK II cells were seeded into 96-well plates, allowed to reach full confluence, before treatment with the compounds, ionomycin (all at a final concentration of 100 µM per well), or DMSO alone as vehicle control. The final DMSO concentration per well was 1%. Following 48 h of incubation, cell viability was assessed using the CellTiter-Glo Luminescent Cell Viability assay (Promega, Walldorf, Germany) following the manufacturer's protocol. Luminescence readings were obtained using black 96-well plates and a Synergy H4 microplate reader (Biotek, Waldbronn, Germany). Relative light units (RLU) were normalized to the DMSO control, which was set at 100%. Triplicate measurements were performed, and the results are expressed as mean + standard deviation.

To determine the IC_{50} of compound 9, we performed a dilution series ranging from 200 to 3.12 µM and tested the compound using the CellTiter-Glo Assay as described above. The IC_{50} value represents the concentration of compound 9 required to inhibit cell viability of MDCK II cells by 50%.

2.3.2. Results

We conducted a cytotoxicity assay with compounds administered at a single high dose of 100 µM in triplicate. In our analysis, any compounds that resulted in a cell viability of less than 80% relative to the DMSO control were categorized as cytotoxic (indicated by the dotted line).

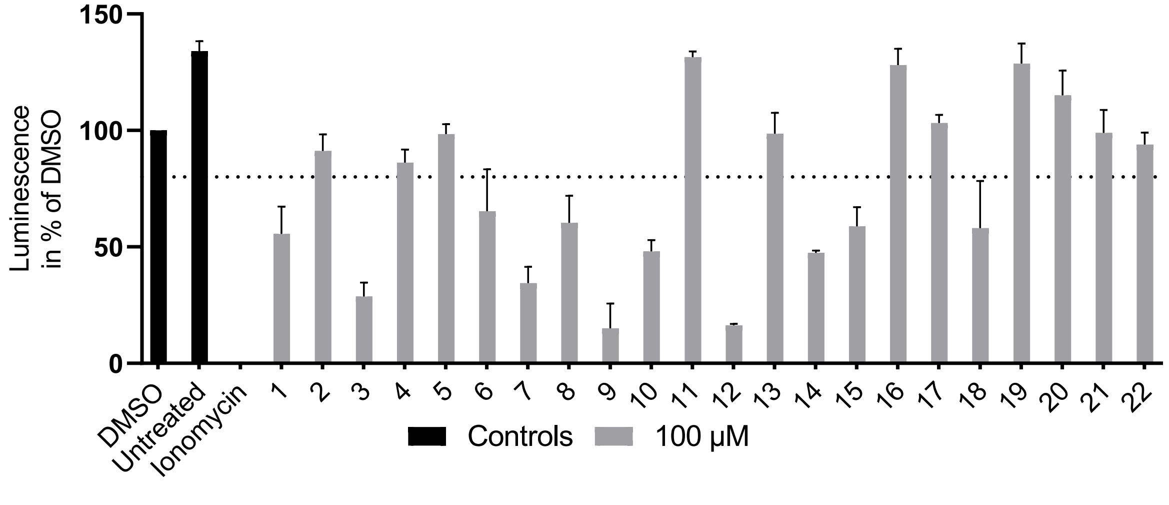

Figure S2: Assessment of cell viability after treatment with compounds. MDCK II cells were treated with 100 µM of compounds, ionomycin, vehicle control or left untreated for 48 h. Luminescence readings were normalized to vehicle control set to 100% viability. Results are presented as mean values with standard deviation (n=3). Data evaluation and visualisation was conducted using GraphPad Prism 9.1.2.

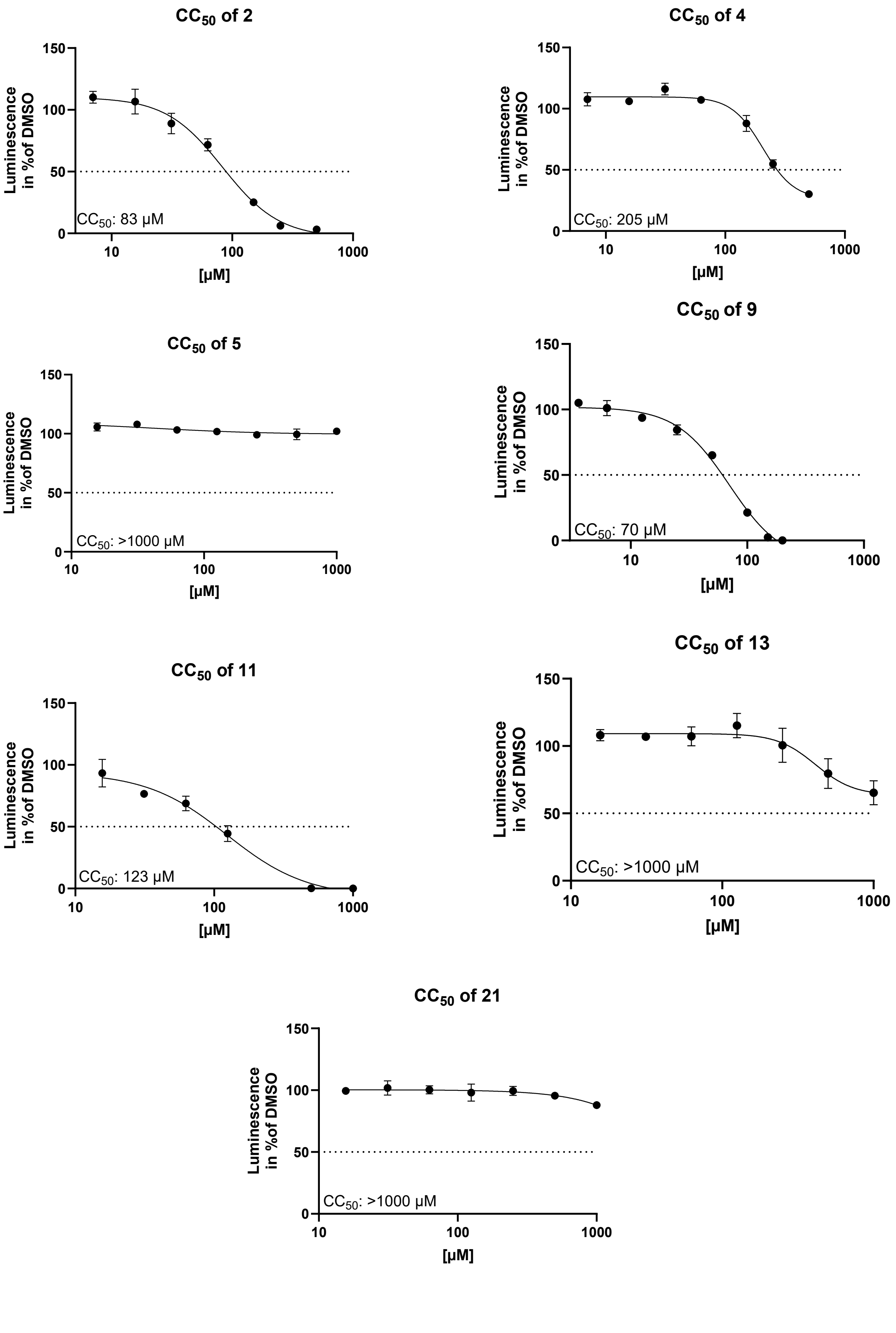

Figure S3: Dose-response curve depicting the inhibition of cell viability by compound 2, 4, 5, 9, 11, 13 and 21 measured by CellTiter-Glo Assay. Each data point represents the mean of 3 independent experiments with standard deviation. The curve was fitted using nonlinear

regression (four parameters. Data evaluation and visualisation was conducted using GraphPad Prism 9.1.2.

3. Experimental procedures

3.1 General remarks

The synthesis of compounds **1**, **2**, **4**, **6**, **8**, **10**-**14** and **16**-**21** has been described in previous publications.[9–14] For biological assays, the respective hydrochloric acid salts of 3-chloropiperidines were used to enhance solubility. For this, a 0.05M solution of the free base in *n*-pentane was prepared. Then, stoichiometric amounts of 2N HCl in Et_2O were added. The resulting suspension was filtered and the solid was dried in vacuo to afford the respective hydrochloric acid salts quantitatively.

3.2 Synthesis of compound 3

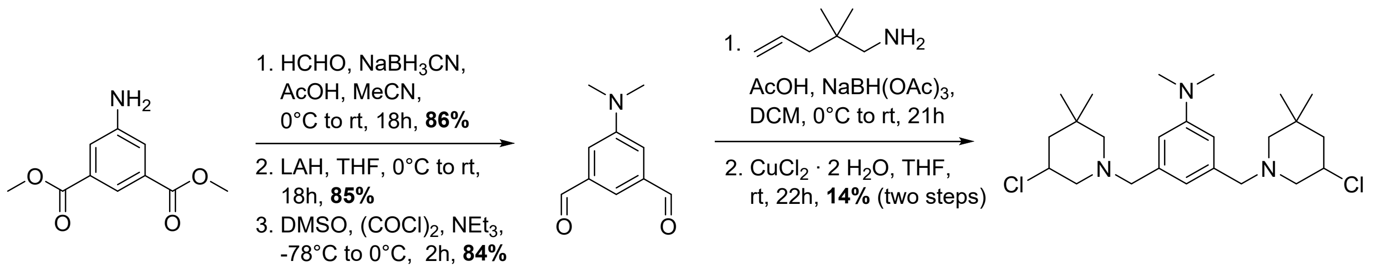

1,3-Dimethyl 5-(dimethylamino)-1,3-benzenedicarboxylate **(3A)**

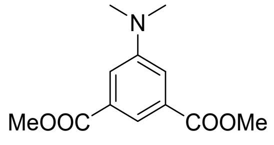

To a solution of 4.180 g (20.00 mmol) dimethyl-5-aminoisophthalate were added 8.93 mL (120 mmol, 6.0 eq.) of a 37% w/w aqueous formaldehyde solution at 0°C. Subsequently, 3.020 g (48.00 mmol, 2.4 eq.) sodium cyanoborohydride was added portionwise a 0°C. The pH was then adjusted to neutrality by the addition of glacial acetic acid. Stirring was continued at

0°C for another 30 minutes. Then, the cooling was discontinued and the mixture was stirred for another 18 hours at room temperature. The mixture was concentrated under reduced pressure and diluted with ethyl acetate and saturated sodium bicarbonate solution. The layers were separated and the organic layer was washed with demineralized water and brine. The organic layer was then dried over magnesium sulfate. After filtration, the filtrate was concentrated under reduced pressure. The crude product was then recrystallized from cyclohexane/EtOAc to yield 4.100 g (17.28 mmol, 86%) 1,3-dimethyl 5-(dimethylamino)-1,3-benzenedicarboxylate as off-white needles. ^{1}H NMR (400 MHz, $CDCl_3$): δ = 8.01 (t, *J* = 1.4 Hz, 1H), 7.57 (d, *J* = 1.4 Hz, 2H), 3.92 (s, 6H), 3.04 (s, 6H) ppm; ^{13}C NMR (101 MHz, $CDCl_3$): δ = 167.0, 150.2, 131.2, 118.6, 117.2, 52.3, 40.6; HRMS(ESI): m/z calculated for $C_{12}H_{15}NO_4Na$ {M+Na$^+$}: 260.0893. Found: 260.0894.

5-(Dimethylamino)-1,3-benzenedimethanol **(3B)**

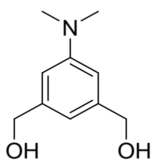

To a suspension of 1.800 g (47.43 mmol, 3.0 eq.) lithium aluminium hydride in 90 mL dry THF was added a solution of 3.750 g (15.81 mmol) **3A** in 40 mL dry THF dropwise at 0°C. After 30 minutes, the cooling was discontinued. After 18 hours of stirring at room temperature, the mixture was quenched by the subsequent addition of 2 mL demineralized water, 2 mL 10% w/w NaOH (aq.) and 6 mL demineralized water. After the addition of magnesium sulfate, the suspension was filtered. The filtrate was concentrated under reduced pressure. The residue was then purified *via* column chromatography (DCM/MeOH 20:1) to obtain 2.425 g (13.38 mmol, 85%) **3B** as a colourless solid. ^{1}H NMR (400 MHz, $CDCl_3$): δ = 6.73 – 6.62 (m, 3H), 4.60 (s, 4H), 2.95 (s, 6H), 2.57 (s, 2H) ppm; ^{13}C NMR (101 MHz, $CDCl_3$): δ = 150.9, 142.3, 114.2, 110.7, 65.6, 40.9 ppm; HRMS(ESI): m/z calculated for $C_{10}H_{16}NO_2$ {M+H$^+$}: 182.1176. Found: 182.1178.

5-(Dimethylamino)-1,3-benzenedicarboxaldehyde **(3C)**

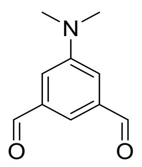

To a solution of 2.13 mL dry DMSO (30.0 mmol, 6.0 eq.) in 30 mL dry DCM was added a solution of 1.36 mL (15.0 mmol, 3.0 eq.) oxalyl chloride in 15 mL dry DCM over 15 minutes at -78°C. The solution was stirred for 30 minutes at the above temperature. Then, a solution of 0.906 g (5.00 mmol) **3B** in 30 mL dry DCM was added over 30 minutes. The resulting solution was then stirred for one hour. Subsequently, 5.58 mL (40.0 mmol, 8.0 eq.) dry triethylamine were added. The solution was then allowed to warm to 0°C over the course of 30 minutes and stirred for another 30 minutes at that temperature. The reaction was then quenched by the addition of 20 mL demineralized water. The layers were separated and the aqueous layer was extracted three times with DCM. The combined organic layers were washed with brine and dried over magnesium sulfate. After filtration, the filtrate was concentrated under reduced pressure. The crude product was then purified via column chromatography (cyclohexane/EtOAc 3:1) to obtain 0.745 g (4.20 mmol, 84%) **3C** as a yellow solid. ^{1}H NMR (400 MHz, $CDCl_3$): δ = 10.03 (s, 2H), 7.68 (t, *J* = 1.4 Hz, 1H), 7.44 (d, *J* = 1.4 Hz, 2H), 3.09 (s, 6H) ppm; ^{13}C NMR (101 MHz, $CDCl_3$): δ = 191.9, 150.8, 137.8, 120.4, 117.0, 40.6 ppm; HRMS(ESI): m/z calculated for $C_{10}H_{11}NO_2Na$ {M+Na$^+$}: 200.0682. Found: 200.0683.

5-Dimethylaminyl-benzene-*N,N*-bis(2,2-dimethyl-pent-4-enyl)-1,3-dimethaneamine **(3D)**

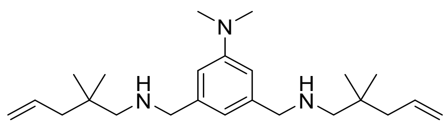

To a solution of 0.710 g (4.01 mmol) **3C** in 40 mL dry DCM were added 0.996 g (8.80 mmol, 2.2 eq.) 2,2-dimethylpent-4-enylamine. Then, 0.5 mL (8.74 mmol, 2.2 eq.) acetic acid and 2.550 g (12.03 mmol, 3.0 eq.) sodium triacetoxyborohydride were added at 0°C. The suspension was stirred for 30 minutes at 0°C and then at room temperature for another 20 hours. The mixture was quenched with a 20% w/w aqueous NaOH solution and extracted two times with DCM. The combined organic layers were washed with brine, water and again with brine. Then, the combined organic layers were dried over magnesium sulfate. After filtration, the filtrate was concentrated under reduced pressure. The crude amine **3D** (1.396 g) was obtained as a slightly

yellow oil and was used in the next step without further purification. ^{1}H NMR (400 MHz, $CDCl_3$): δ = 6.77 – 6.60 (m, 3H), 5.89 – 5.64 (m, 2H), 5.11 – 4.87 (m, 4H), 3.77 (s, 4H), 2.95 (s, 6H), 2.39 (s, 4H), 2.03 (dt, *J* = 7.4, 1.3 Hz, 4H), 0.90 (s, 12H) ppm; ^{13}C NMR (101 MHz, $CDCl_3$): δ = 149.9, 134.5, 115.8, 110.0, 53.9, 43.7, 39.8, 33.3, 24.5 ppm; HRMS(ESI): m/z calculated for $C_{24}H_{42}N_3$ {M+H+}: 372.3373. Found: 372.3373.

5-Dimethylaminyl-1,3-bis-[(3-Chloro-5,5-dimethylpiperidin-1-yl)methyl]benzene **(3)**

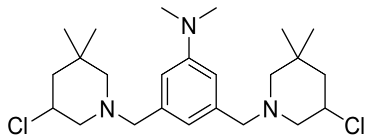

To a solution of 1.020 g (2.745 mmol) **3D** in 60 mL THF were added 0.738 g (5.49 mmol, 2.0 eq.) copper(II) chloride and 0.2 mL (11 mmol, 4 eq.) demineralized water. The solution was stirred for 20 hours until another 0.738 g copper(II) chloride and 0.2 mL water were added. After additional stirring for two hours, 30 mL water and 30 mL conc. ammonia were added. The resulting emulsion was then extracted three times with DCM. The combined organic layers were washed once with water and brine and then dried over magnesium sulfate. After filtration, the filtrate was concentrated under reduced pressure. The crude residue was then purified by column chromatography (pentane/TBME 10:1) to obtain 0.180 g (0.409 mmol, 15%) **3** as a colourless oil. ^{1}H NMR (400 MHz, $CDCl_3$): δ = 6.60 (s, 3H), 4.11 (m, 2H), 3.53 (dd, *J* = 13.4, 7.1 Hz, 2H), 3.38 (dd, *J* = 13.4, 8.4 Hz, 2H), 3.18 (s, 2H), 2.94 (s, 6H), 2.41 (d, *J* = 10.0 Hz, 2H), 2.01 (td, *J* = 10.4, 3.6 Hz, 2H), 1.97 – 1.88 (m, 2H), 1.80 – 1.64 (m, 2H), 1.35 (t, *J* = 12.3 Hz, 2H), 1.07 (d, *J* = 2.7 Hz, 6H), 0.95 – 0.84 (m, 6H) ppm; ^{13}C NMR (101 MHz, $CDCl_3$): δ = 138.3, 116.4, 110.5, 63.4, 63.3, 61.7, 61.2, 61.1, 53.3, 47.5, 39.7, 32.4, 28.3, 24.1 ppm; HRMS(ESI): m/z calculated for $C_{24}H_{39}N_3Cl_2$ {M+H+}: 440.2594. Found: 440.2597.

3.3 Synthesis of compound 5

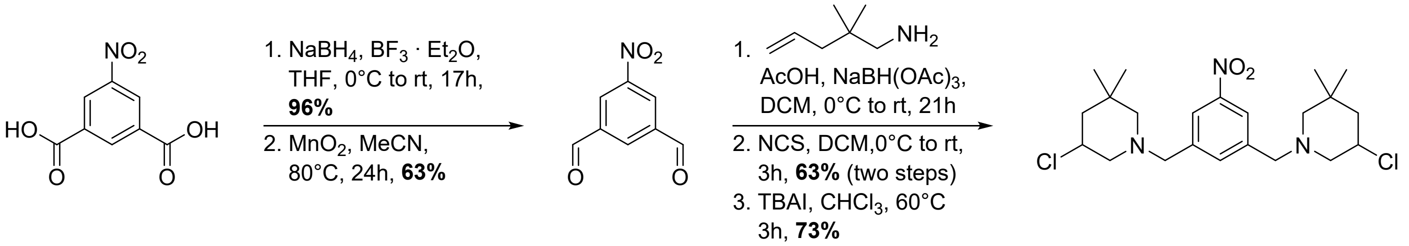

5-Nitro-1,3-benzenedimethanol **(5A)**

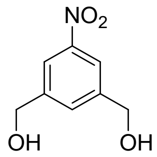

To a solution of 3.167 g (15.00 mmol) 5-nitroisophthalic acid in 60 mL dry THF were added 1.710 g (45.00 mmol, 3.0 eq.) sodium borohydride portion wise. Subsequently, 5.55 mL (45.00 mmol, 3.0 eq.) borontrifluoride etherate were added at 0°C over the course of 30 minutes. The resulting mixture was stirred for one hour at 0°C. The cooling was then ceased and the mixture was stirred for another 16 hours at room temperature. Then, the mixture was quenched by the addition of 20 mL 20% NaOH (aq.) at 0°C. The suspension was initially allowed to stir for 30 minutes at 0°C, until the ice bath was removed. After another 30 minutes of stirring, 100 mL ethyl acetate was added. The layers were separated and the aqueous layer was extracted three times with ethyl acetate. The combined organic layers were washed with brine and dried over magnesium sulfate. After filtration, the filtrate was concentrated under reduced pressure to obtain crude **5A** (2.628 g, 14.35 mmol, 96%), which was used in the following synthetic step without further purification. ^{1}H NMR (400 MHz, MeOD): δ = 8.27 – 8.04 (m, 2H), 7.75 – 7.63 (m, 1H), 4.71 (s, 4H) ppm; ^{13}C NMR (101 MHz, MeOD): δ = 148.6, 144.1, 130.3, 119.5, 62.6 ppm; HRMS(ESI): m/z calculated for $C_8H_9NO_4Na$ {$M+Na^+$}: 206.0424. Found: 206.0423.

5-Nitro-1,3-benzenedicarboxaldehyde **(5B)**

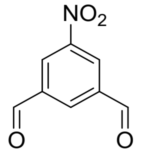

To a solution of 1.282 g (6.999 mmol) **5A** in 30 mL dry MeCN was added 6.086 g (70.00 mmol, 10 eq.) activated manganese(IV) oxide. The resulting suspension was stirred for 24 hours at 80°C. Then, silica was added and the solvent was removed under reduced pressure. The resulting dispersion was put on a column and purified by elution with cyclohexane/EtOAc (2:1) to obtain 0.780 g (4.35 mmol, 63%) **5B** as a colourless solid. ^{1}H NMR (400 MHz, $CDCl_3$): δ = 10.21 (s, 2H), 8.95 (d, *J* = 1.5 Hz, 2H), 8.72 (t, *J* = 1.5 Hz, 1H) ppm; ^{13}C NMR (101 MHz, $CDCl_3$): δ = 188.5, 149.5, 138.3, 134.6, 128.6 ppm.

5-Nitro-benzene-*N,N*-bis(2,2-dimethyl-pent-4-enyl)-1,3-dimethaneamine **(5C)**

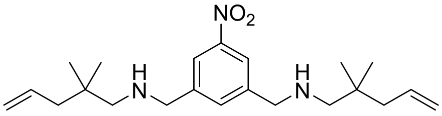

To a solution of 0.750 g (4.18 mmol) **5B** in 40 mL dry DCM were added 1.04 g (9.21 mmol, 2.2 eq.) 2,2-dimethylpentenylamine. Then, 0.53 mL (9.21 mmol, 2.2 eq.) acetic acid and 2.660 g (12.56 mmol, 3.0 eq.) sodium triacetoxyborohydride were added at 0°C. The suspension was stirred for 30 minutes at 0°C and then at room temperature for another 20 hours. The mixture was quenched with a 20% w/w aqueous NaOH solution and extracted two times with DCM. The combined organic layers were washed with brine, water and again with brine. Then, the combined organic layers were dried over magnesium sulfate. After filtration, the filtrate was concentrated under reduced pressure. The crude product (1.542 g) was used in the next step without further purification. ^{1}H NMR (400 MHz, $CDCl_3$): δ = 8.10 (d, *J* = 1.6 Hz, 2H), 7.77 – 7.71 (m, 1H), 5.91 – 5.66 (m, 2H), 5.11 – 4.93 (m, 4H), 3.89 (s, 4H), 2.37 (s, 4H), 2.04 (dt, *J* = 7.5, 1.2 Hz, 4H), 0.92 (s, 12H) ppm; ^{13}C NMR (101 MHz, $CDCl_3$): δ = 148.5, 135.2, 133.9, 121.6, 117.1, 59.4, 53.6, 44.6, 34.4, 25.5 ppm; HRMS(ESI): m/z calculated for $C_{22}H_{36}N_3O_2$ {M+H$^+$}: 374.2802. Found: 374.2800.

5-Nitro-benzene-*N,N*-dichloro-*N,N*-bis(2,2-dimethyl-pent-4-enyl)-1,3-dimethaneamine **(5D)**

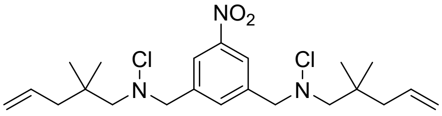

To a solution of 1.53 g (4.09 mmol) **5C** in 40 mL dry DCM were added 1.31 g (9.83 mmol) NCS portion wise at 0°C. The solution was stirred for 30 minutes at 0°C and then at room temperature for 2.5 hours. Silica gel was added and the suspension was concentrated in vacuo. The crude residue was then purified *via* column chromatography (pentane/TBME 10:1) to obtain **5D** (1.155 g, 2.611 mmol, 64%) as a slightly yellow oil. ^{1}H NMR (400 MHz, $CDCl_3$): δ = 8.16 (d, *J* = 1.5 Hz, 2H), 7.76 – 7.71 (m, 1H), 5.80 (ddt, *J* = 16.3, 10.8, 7.5 Hz, 2H), 5.15 – 4.97 (m, 4H), 4.19 (s, 4H), 2.99 (s, 4H), 2.11 (dt, *J* = 7.5, 1.2 Hz, 4H), 0.99 (s, 12H) ppm; ^{13}C NMR (101 MHz, $CDCl_3$): δ = 148.4, 139.7, 135.4, 135.0, 123.2, 117.6, 74.0, 69.3, 44.9, 44.9, 44.7, 35.8, 25.8 ppm; HRMS(ESI): m/z calculated for $C_{22}H_{34}N_3O_2Cl_2$\{M+H$^+$\}: 442.2023. Found: 442.2026.

5-Nitro-1,3-bis-[(3-Chloro-5,5-dimethylpiperidin-1-yl)methyl]benzene **(5)**

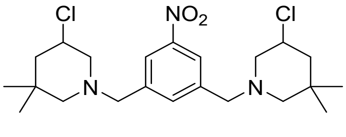

To a solution of 1.14 g (2.58 mmol) **5D** in 25 mL dry chloroform were added 0.095 g (0.26 mmol, 0.1 eq.) TBAI. The solution was then heated to 60°C for 2.5 hours. The solution was then dispersed on silica gel and purified by column chromatography (n-pentane/TBME 10:1) to afford 0.837 g (1.89 mmol, 73%) **5** as a slightly yellow oil. ^{1}H NMR (400 MHz, $CDCl_3$): δ = 8.07 (s, 2H), 7.68 – 7.50 (m, 1H), 4.18 – 4.04 (m, 2H), 3.69 – 3.45 (m, 4H), 3.18 – 3.08 (m, 2H), 2.35 (t, *J* = 9.6 Hz, 2H), 2.07 (td, *J* = 10.6, 3.9 Hz, 2H), 2.02 – 1.92 (m, 2H), 1.82 (dd, *J* = 11.2, 5.7 Hz, 2H), 1.37 (t, *J* = 12.3 Hz, 2H), 1.07 (s, 6H), 0.91 (s, 6H) ppm; ^{13}C NMR (101 MHz, $CDCl_3$): δ = 148.6, 140.9, 134.6, 122.2, 64.6, 61.9, 61.4, 53.7, 48.1, 33.4, 29.2, 25.1; HRMS(ESI): m/z calculated for $C_{22}H_{34}N_3O_2Cl_2$ \{M+H$^+$\}: 442.2023. Found: 442.2021.

3.4 Synthesis of compound 7

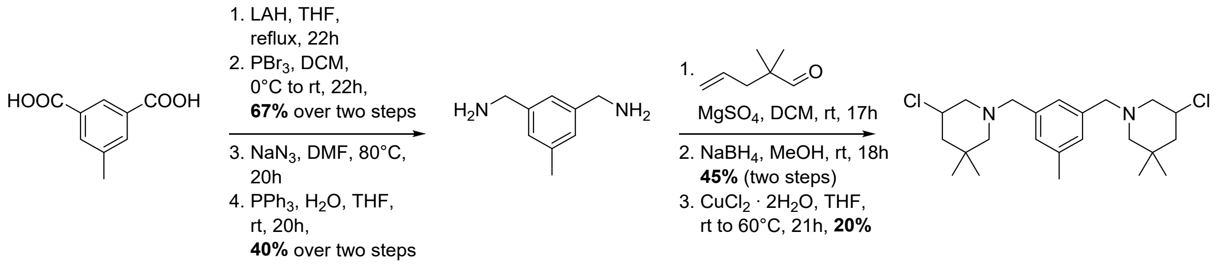

1,3-Bis(bromomethyl)-5-methylbenzene **(7A)**

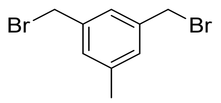

To a suspension of 0.931 g (24.53 mmol) lithium aluminium hydride in 50 mL THF was slowly added a solution of 2.000 g (11.10 mmol) 5-methyl-1,3-benzenedicarboxylic acid. The mixture was then heated under reflux. After 22 hours, the reaction was completed by the addition of 1 mL water at 0°C, followed by 1 mL 20% NaOH (aq.) and again 3 mL water. After the addition of magnesium sulfate, the suspension was filtered. The filtrate was concentrated under reduced pressure. The obtained residue was diluted with 50 mL DCM. Then, 0.53 mL (5.6 mmol) PBr_3 was added slowly at 0°C. The ice-bath was removed immediately after the addition. After 22 hours, the reaction was completed by the slow addition of 20 mL saturated sodium bicarbonate solution another 30 minutes of stirring. The mixture was then extracted three times with DCM. The combined organic layers were washed with brine and dried over magnesium sulfate. After filtration, the filtrate was concentrated under reduced pressure to obtain 2.077 g (7.47 mmol, 67% over two steps) **7A**, which was used in the next step without further purification. ^{1}H NMR (400 MHz, $CDCl_3$): δ = 7.22 (d, *J* = 1.7 Hz, 1H), 7.14 (d, *J* = 1.7 Hz, 2H), 4.45 (s, 4H), 2.39 – 2.31 (m, 3H) ppm; ^{13}C NMR (101 MHz, $CDCl_3$): δ = 139.3, 138.3, 129.9, 126.7, 33.0, 21.2 ppm.

5-Methyl-1,3-benzenedimethanamine **(7B)**

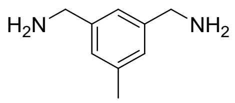

To a solution of 2.077 g (7.47 mmol) **7A** in 150 mL DMF were added 1.068 g (16.43 mmol) sodium azide. The suspension was then heated to 80°C for 20 hours. Then, 70 mL demineralized water was added. The solution was extracted three times with ethyl acetate. The combined organic layers were washed with brine and dried over magnesium sulfate. After filtration, the filtrate was concentrated under reduced pressure to a volume of approximately 20 mL. The obtained solution was diluted with 100 mL THF. Then, 5.878 g (22.411 mmol) triphenylphosphane were added. After one hour of stirring 5 mL water were added and the mixture was stirred for another 20 hours. The solution was then extracted three times with 2N HCl (aq.). The combined aqueous layers were washed once with ethyl acetate. Then the aqueous layer was basified to pH14 by the addition of potassium hydroxide pellets. The resulting emulsion was extracted three times with ethyl acetate. The combined organic layers were washed with brine and dried over magnesium sulfate. After filtration, the filtrate was concentrated under reduced pressure to obtain crude **7B**, which was purified *via* bulb-to-bulb distillation (1 mbar, 175°C) to obtain 0.448 g (2.98 mmol, 40% over two steps) **7B**. ^{1}H NMR (400 MHz, $CDCl_3$): δ = 7.06 (s, 1H), 7.01 (s, 2H), 3.83 (s, 4H), 2.34 (s, 3H), 1.46 (bs, 4H) ppm; ^{13}C NMR (101 MHz, $CDCl_3$): δ = 143.7, 138.5, 126.4, 122.9, 46.5, 21.3 ppm; HRMS(ESI): m/z calculated for $C_9H_{15}N_2O\{M+H^+\}$: 167.1179. Found: 167.1180.

5-Methyl-benzene-*N,N*-bis(2,2-dimethyl-pent-4-enyl)-1,3-dimethaneamine **(7C)**

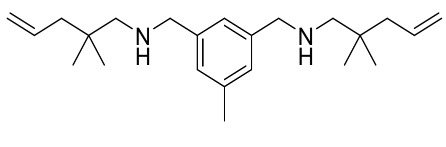

To a solution of 0.500 g (3.33 mmol) **7B** in 40 mL DCM were added 0.812 g (7.24 mmol, 2.2 eq.) 2,2-dimethylpent-4-enal and 5 g magnesium sulfate. The suspension was stirred for 17 hours at room temperature. After filtration, the filtrate was concentrated under reduced pressure. The residue was taken up in 40 mL methanol and 0.378 g (9.99 mmol, 3.0 eq.) sodium borohydride were added. After 18 hours, 20 mL 20% w/w aqueous NaOH were added. The

solution was then extracted three times with DCM. The combined organic layers were washed with brine and dried over magnesium sulfate. After filtration, the filtrate was concentrated under reduced pressure. The crude residue was then purified via column chromatography (DCM/MeOH 15:1+1% NEt_3) to afford 0.519 g (1.52 mmol, 45%) **7C** as a colourless oil. ^{1}H NMR (400 MHz, $CDCl_3$): δ = 7.15 – 7.02 (m, 3H), 5.87 – 5.69 (m, 2H), 5.08 – 4.94 (m, 4H), 3.77 (s, 4H), 2.38 (s, 4H), 2.34 (s, 3H), 2.03 (dt, *J* = 7.4, 1.2 Hz, 4H), 0.90 (s, 13H) ppm; ^{13}C NMR (101 MHz, $CDCl_3$): δ = 135.5, 127.6, 125.0, 116.9, 77.2, 59.4, 54.4, 44.6, 34.3, 25.5, 21.4, 1.0 ppm; HRMS(ESI): m/z calculated for $C_{23}H_{39}N_2\{M+H^+\}$: 343.3108. Found: 343.3106.

5-Methyl-1,3-bis-[(3-Chloro-5,5-dimethylpiperidin-1-yl)methyl]benzene **(7)**

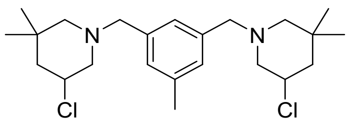

To a solution of 0.345 g (1.01 mmol) **7C** in 50 mL THF were added 0.542 g copper(II) chloride (4.03 mmol) and 0.14 mL (7.77 mmol) demineralized water. The solution was stirred for 18 hours at room temperature and subsequently heated to 60 °C for 3 hours. Then, 30 mL conc. ammonia were added and the resulting solution was extracted three times with DCM. The combined organic layers were washed two times with water and once with brine. Afterwards, they were dried over sodium sulfate. After filtration, the filtrate was concentrated under reduced pressure. Purification *via* column chromatography yielded 0.084 g (0.20 mmol, 20%) **7** as a slightly yellow oil. ^{1}H NMR (400 MHz, $CDCl_3$): δ = 7.13 – 6.90 (m, 3H), 4.10 (ddd, *J* = 15.0, 10.4, 4.4 Hz, 2H), 3.60 – 3.34 (m, 4H), 3.22 – 3.09 (m, 2H), 2.54 – 2.20 (m, 6H), 1.96 (qd, *J* = 11.8, 4.6 Hz, 4H), 1.74 (dd, *J* = 11.0, 6.4 Hz, 2H), 1.44 – 1.27 (m, 3H), 1.06 (s, 6H), 0.89 (s, 6H) ppm; ^{13}C NMR (101 MHz, $CDCl_3$): δ = 138.4, 128.2, 126.2, 64.6, 64.5, 62.3, 62.0, 61.9, 54.3, 48.4, 33.4, 29.3, 25.1, 21.4 ppm; HRMS(ESI): m/z calculated for $C_{23}H_{37}N_2Cl_2\{M+H^+\}$: 411.2329. Found: 411.2326.

3.5 Synthesis of compound 9

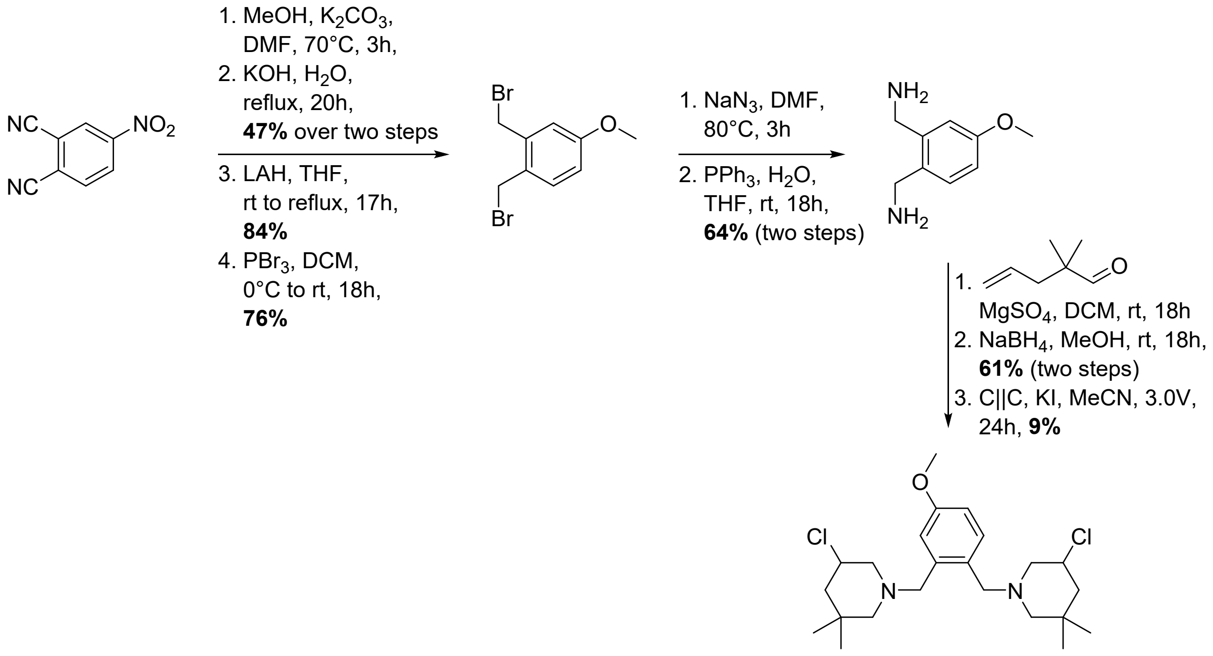

4-Methoxyphthalic acid **(9A)**

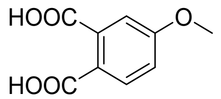

To a solution of 8.000 g (46.21 mmol) 4-nitrophthalonitrile in 300 mL DMF were added 24.000 g (173.66 mmol) and 20 mL MeOH. The suspension was heated at 70°C for three hours. Then, the mixture was poured on 500 mL ice-cold water. The precipitate was filtered off and washed thoroughly with water. The wet solid was suspended in 150 mL water and 15.556 g (277.24 mmol) potassium hydroxide were added. The suspension was refluxed for 20 hours and then acidified to pH 2 by the slow addition of concentrated hydrochloric acid at 0°C. The precipitated crystals were filtered off and recrystallized from $CHCl_3$/MeOH. After drying in vacuo at 60°C, 4.294 g (21.89 mmol, 47%) 4-methoxyphthalic acid **9A** was obtained as a slightly green solid. ^{1}H NMR (400 MHz, MeOH-d_4): δ = 7.73 (d, *J* = 8.5 Hz, 1H), 7.10 – 6.90 (m, 2H), 3.78 (s, 3H) ppm; ^{13}C NMR (101 MHz, MeOH-d_4): δ = 170.8, 168.4, 162.2, 136.7, 131.5, 122.4, 114.7, 113.4, 54.8 ppm; HRMS(ESI): m/z calculated for $C_9H_9O_5$ {M-H$^+$}: 195.0299. Found: 195.0300.

4-Methoxy-1,2-benzenedimethanol **(9B)**

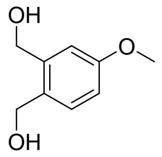

To a suspension of 0.929 g (24.5 mmol) lithium aluminium hydride in 50 mL dry THF was added a solution of 2.000 g (10.20 mmol) **9A** in 30 mL dry THF at room temperature. The suspension was left to stir at room temperature for one hour. The mixture was then heated to reflux for 16 hours. To complete the reaction, 1 mL water was added at 0°C, followed by 1 mL 20% NaOH (aq.) and again 3 mL water. After addition of magnesium sulfate, the suspension was filtered. The filtrate was concentrated under reduced pressure to obtain 1.443 g (8.579 mmol, 84%) **9B**, which was used in the next step without further purification. ^{1}H NMR (400 MHz, $CDCl_3$): δ = 7.19 – 7.13 (m, 1H), 6.85 (d, *J* = 2.7 Hz, 1H), 6.74 (dd, *J* = 8.3, 2.7 Hz, 1H), 4.66 – 4.56 (m, 4H), 3.74 (s, 3H) ppm; ^{13}C NMR (101 MHz, $CDCl_3$): δ = 159.7, 141.2, 131.5, 131.2, 115.6, 113.0, 64.3, 63.7, 55.4 ppm; HRMS(ESI): m/z calculated for $C_9H_{12}O_3Na$ {M+Na$^+$}: 191.0678. Found: 191.0678.

1,2-Bis(bromomethyl)-4-methoxybenzene **(9C)**

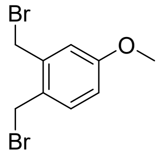

To a solution of 1.443 g (8.579 mmol) **9B** in 90 mL DCM was added 1.63 mL (17.2 mmol) PBr_3 at 0°C. The ice bath was then removed and the solution was stirred at room temperature for 18 hours. Then, 20 mL saturated sodium bicarbonate solution were added and the resulting emulsion was stirred for another 30 minutes. The layers were then separated and the aqueous layer was extracted three times with DCM. The combined organic layers were washed with brine and dried over magnesium sulfate. After filtration, the filtrate was concentrated under reduced pressure to obtain 1.929 g (6.562 mmol, 76%) **9C**. The crude product was sufficiently pure to be used in the next step without further purification. ^{1}H NMR (400 MHz, $CDCl_3$): δ = 7.29 (d, *J* = 8.4 Hz, 1H), 6.90 (d, *J* = 2.7 Hz, 1H), 6.83 (dd, *J* = 8.4, 2.7 Hz, 1H), 4.66 (s, 2H), 4.62 (s, 2H), 3.82 (s, 3H) ppm; ^{13}C NMR (101 MHz, $CDCl_3$): δ = 160.2, 138.1, 132.6, 128.6, 116.5,

114.7, 55.4, 30.6, 30.1 ppm; HRMS(ESI): m/z calculated for $C_{10}H_{13}O_2BrNa$ {M+Na$^+$}: 266.9991. Found: 266.9986 (degraded on ESI by mono-methoxylation).

4-Methoxy-1,2-benzenedimethanamine **(9D)**

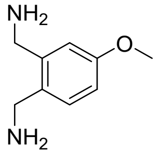

To a solution of 8.584 g (29.20 mmol) **9C** in 200 mL DMF were added 3.910 g (60.14 mmol) sodium azide. The mixture was heated to 80°C for three hours. Then, 100 mL demineralized water were added. The solution was extracted three times with ethyl acetate. The combined organic layers were washed with brine and dried over magnesium sulfate. After filtration, the filtrated was concentrated to 20% of the original volume (approximately 20 mL) under reduced pressure. The resulting solution was diluted with 200 mL THF and 17.927 g (68.350 mmol) triphenylphosphane were added. After one hour 10 mL water were added. After another 18 hours, the mixture was extracted three times with 2N HCl (aq.). The aqueous layer was then basified to pH14 with potassium hydroxide pellets. The emulsion was extracted three times with DCM. The combined organic layers were then washed with brine and dried over magnesium sulfate. After filtration, the filtrate was concentrated under reduced pressure to obtain 3.090 g (18.59 mmol, 64% over two steps) **9D**, which was used in the next step without further purification. ^{1}H NMR (400 MHz, $CDCl_3$): δ = 7.11 – 6.94 (m, 3H), 3.83 (s, 4H), 2.35 (s, 3H), 1.48 (s, 4H) ppm; ^{13}C NMR (101 MHz, $CDCl_3$): δ = 143.7, 138.5, 126.4, 122.9, 46.5, 21.3 ppm; HRMS(ESI): m/z calculated for $C_9H_{15}N_2O$ {M+H$^+$}: 151.1230. Found: 151.1232.

4-Methoxy-benzene-*N,N*-bis(2,2-dimethyl-pent-4-enyl)-1,2-dimethaneamine **(9E)**

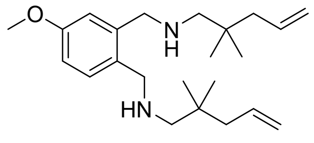

To a solution of 1.641 g (6.562 mmol) **9D** in 100 mL DCM were added 1.619 g (14.43 mmol) 2,2-dimethylpent-4-enal and 5 g magnesium sulfate. After 18h, the suspension was filtered and the filtrate was concentrated under reduced pressure. The residue was taken up in 50 mL MeOH and 0.744 g sodium borohydride were added at 0°C. The mixture was then allowed to warm to room temperature. After 23h, 20 mL 20% w/w aqueous NaOH solution was added and the resulting solution was extracted three times with DCM. The combined organic layers were washed with brine and dried over magnesium sulfate. After filtration, the filtrate was concentrated under reduced pressure to obtain 1.428 g (3.98 mmol, 61%) **9E** as a colourless oil. The hydrochloride salt of the product was then precipitated with ethereal HCl (2N) for the use in the next step. ^{1}H NMR (400 MHz, $CDCl_3$): δ = 7.15 (d, *J* = 8.3 Hz, 1H), 6.85 (d, *J* = 2.7 Hz, 1H), 6.70 (dd, *J* = 8.3, 2.7 Hz, 1H), 5.78 – 5.64 (m, 2H), 4.98 – 4.89 (m, 4H), 3.80 – 3.69 (m, 7H), 2.44 – 2.29 (m, 4H), 1.94 (dd, *J* = 7.5, 3.8 Hz, 4H), 0.83 (s, 6H), 0.83 (s, 6H) ppm; ^{13}C NMR (101 MHz, $CDCl_3$): δ = 158.9, 135.3, 131.3, 117.1, 111.9, 60.0, 59.6, 55.3, 52.8, 52.2, 44.7, 34.4, 34.3, 25.5, 24.7 ppm; HRMS(ESI): m/z calculated for $C_{23}H_{39}N_2O$ {M+H$^+$}: 359.3057. Found: 359.3061.

4-Methoxy-1,2-bis-[(3-Chloro-5,5-dimethylpiperidin-1-yl)methyl]benzene **(9)**

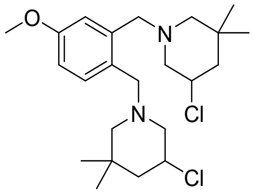

A 20 mL cylindric beaker-type cell was charged with 0.045 g (0.27 mmol) potassium iodide, 0.806 g (1.87 mmol) **9E · 2 HCl** and 20 mL acetonitrile. The cell was then equipped with two graphite electrodes (110 mm long, 8mm diameter, 8mm distance). The suspension was stirred for 24 hours under room temperature and a cell potential of 3.0 V. The mixture was then concentrated under reduced pressure and the product (0.067 g, 0.16 mmol, 9%) was obtained via column chromatography (pentane/TBME 5:1) as a slightly yellow oil. ^{1}H NMR (400 MHz, $CDCl_3$): δ = 7.14 (d, *J* = 8.4 Hz, 1H), 7.00 – 6.92 (m, 1H), 6.74 (dd, *J* = 8.4, 2.7 Hz, 1H), 4.17 – 3.94 (m, 2H), 3.81 (s, 3H), 3.77 – 3.30 (m, 4H), 3.19 – 2.98 (m, 2H), 2.47 – 2.33 (m, 2H), 2.08 – 1.85 (m, 4H), 1.85 – 1.65 (m, 2H), 1.37 (td, *J* = 12.3, 5.5 Hz, 2H), 1.15 – 0.97 (m, 6H), 0.91 (s, 6H) ppm; ^{13}C NMR (101 MHz, $CDCl_3$): δ = 158.7, 139.1, 131.5, 131.4, 129.1, 129.1, 115.6, 115.5,

111.5, 111.5, 65.3, 64.9, 64.9, 62.2, 61.8, 61.4, 59.6, 59.6, 59.5, 55.2, 54.3, 54.2, 48.4, 48.4, 33.5, 33.5, 33.4, 29.3, 25.4, 25.4 ppm; HRMS(ESI): m/z calculated for $C_{23}H_{37}N_2OCl_2$ {M+H$^+$}: 427.2278. Found: 427.2278.

3.6 Synthesis of compound 15

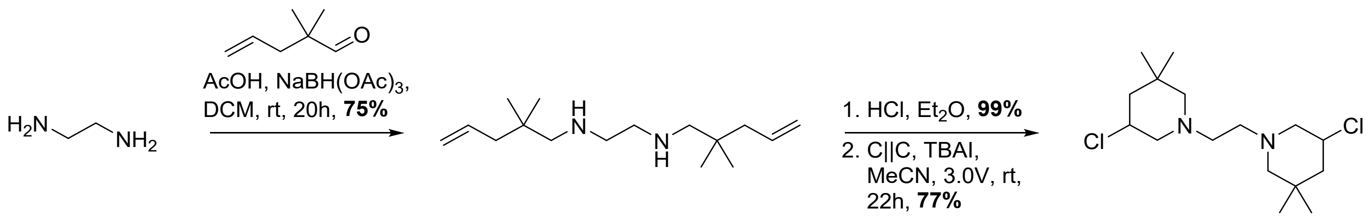

N,N-bis(2,2-dimethyl-pent-4-enyl)-ethylendiamine **(15A)**

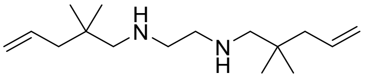

To a solution of 4.106 g (36.60 mmol) 2,2-dimethylpent-4-enal in 150 mL DCM were added 1.11 mL (16.6 mmol) 1,2-ethylendiamine, 1.91 mL (33.4 mmol) glacial acetic acid and 8.821 g (41.60 mmol) sodium triacetoxyborohydride. After 20 hours, 40 mL 20% w/w aqueous NaOH solution was added und the resulting solution was extracted three times with DCM. The combined organic layers were washed with brine and dried over magnesium sulfate. After filtration, the filtrate was concentrated under reduced pressure to obtain the product (4.010 g, 12.48 mmol, 75%) as a colourless oil. The hydrochloride salt of the product was then precipitated with ethereal HCl (2N) for the use in the next step. ^{1}H NMR (400 MHz, $CDCl_3$): δ = 5.95 – 5.69 (m, 2H), 5.10 – 4.93 (m, 4H), 2.81 – 2.69 (m, 4H), 2.38 (s, 4H), 2.01 (dt, *J* = 7.5, 1.2 Hz, 4H), 0.90 (s, 12H) ppm; ^{13}C NMR (101 MHz, $CDCl_3$): δ = 135.3, 117.0, 60.0, 49.4, 44.8, 34.3, 25.5 ppm; HRMS(ESI): m/z calculated for $C_{16}H_{33}N_2$ {M+H$^+$}: 253.2638. Found: 253.2638.

1,2-bis-[(3-Chloro-5,5-dimethylpiperidin-1-yl)ethylendiamine **(15)**

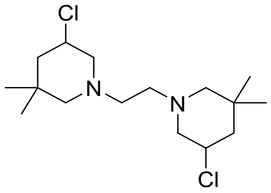

A 20 mL cylindric beaker-type cell was charged with 0.200 g (0.541 mmol) TBAI, 0.651 g (2.00 mmol) of the hydrochloric acid salt of **15A** and 20 mL acetonitrile (0.5% water). The cell was then equipped with two graphite electrodes (110 mm long, 8mm diameter, 8mm distance). The suspension was stirred for 22 hours under room temperature and 3.0 V. The mixture was then concentrated under reduced pressure and the product (0.498 g, 1.55 mmol, 77%) was obtained after column chromatography (pentane/TBME 7:1) as a slightly yellow oil. ^{1}H NMR (400 MHz, $CDCl_3$): δ = 4.12 – 3.96 (m, 2H), 3.18 (d, J = 10.4 Hz, 2H), 2.57 – 2.33 (m, 6H), 2.08 – 1.87 (m, 4H), 1.77 (dd, J = 11.5, 3.2 Hz, 2H), 1.43 – 1.23 (m, 2H), 1.01 (s, 6H), 0.91 (s, 6H) ppm; ^{13}C NMR (101 MHz, $CDCl_3$): δ = 65.2, 65.1, 62.4, 55.5, 55.4, 54.2, 48.3, 33.3, 29.4, 25.2 ppm; HRMS(ESI): m/z calculated for $C_{16}H_{31}Cl_2N_2$ {M+H$^+$}: 321.1859. Found: 321.1857.

3.7 Synthesis of control compound 22

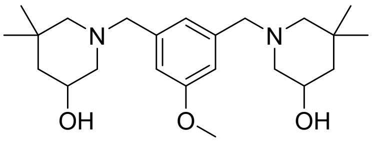

To a solution of 0.214 g (0.501 mmol) **2** in 10 mL dioxane was added 2 mL (2 mmol, 4 eq.) 1M aqueous sodium hydroxide solution. The solution was then stirred at 70°C. After 22 hours, water was added and the resulting solution was extracted three times with DCM. The combined organic layers were dried over magnesium sulfate. After filtration, the filtrate was concentrated under reduced pressure. The crude residue was then subjected to gradient column chromatography (DCM/MeOH 20:1 to 15:1 to 10:1) to obtain 0.074 g (0.19 mmol, 38%) **22** as a colorless solid. ^{1}H NMR (600 MHz, $CDCl_3$): δ =6.87 (s, 1H), 6.80 (s, 2H), 4.02 – 3.89 (m, 2H), 3.79 (s, 3H), 3.56 – 3.44 (m, 4H), 2.97 – 2.84 (m, 2H), 2.34 – 2.24 (m, 2H), 1.93 – 1.77 (m, 4H), 1.73 – 1.63 (m, 2H), 1.17 – 1.06 (m, 2H), 1.02 (s, 6H), 0.94 (s, 6H) ppm; ^{13}C NMR (151 MHz, $CDCl_3$): δ = 159.7, 121.6, 112.9, 65.9, 65.0, 62.5, 62.4, 61.2, 55.2, 46.4,

31.9, 29.4, 29.4, 26.6; HRMS(ESI): m/z calculated for $C_{23}H_{39}N_2O_3$ {M+H+}: 391.2955. Found: 391.2959.

4. Copies of spectra

Compound 3A

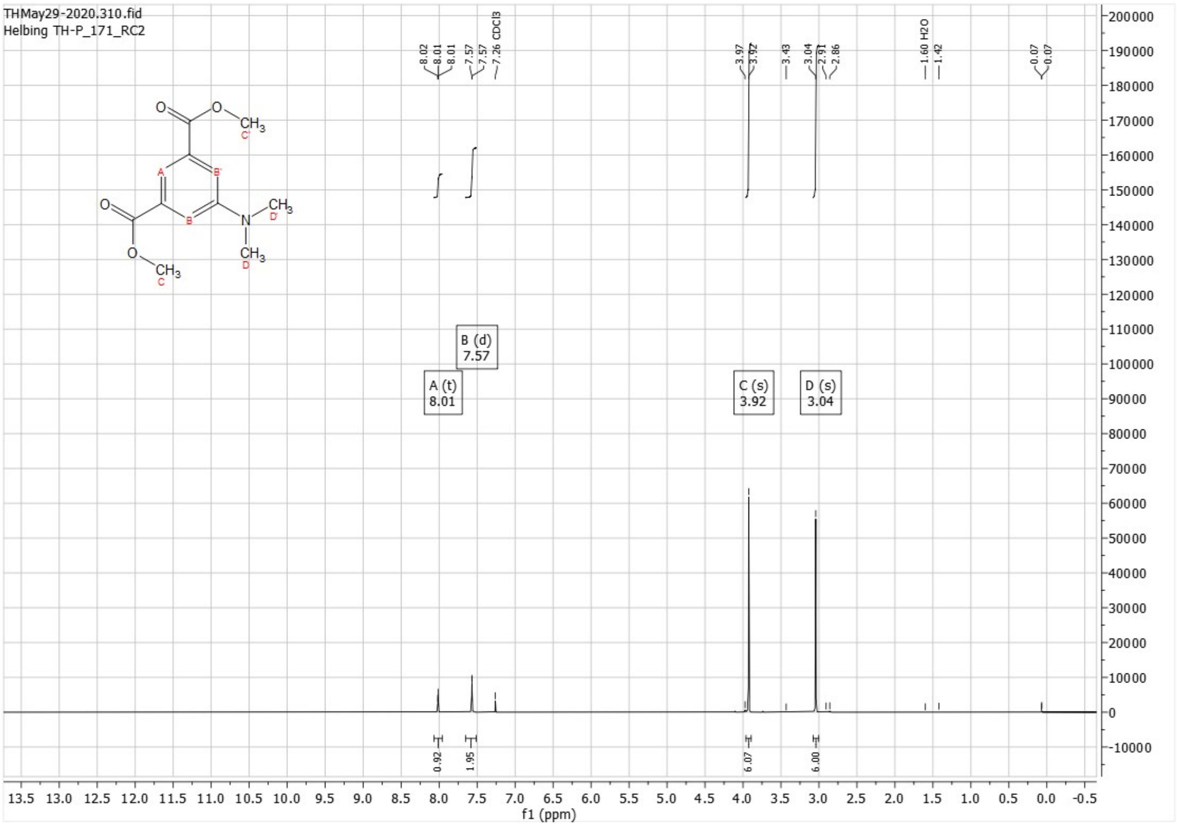

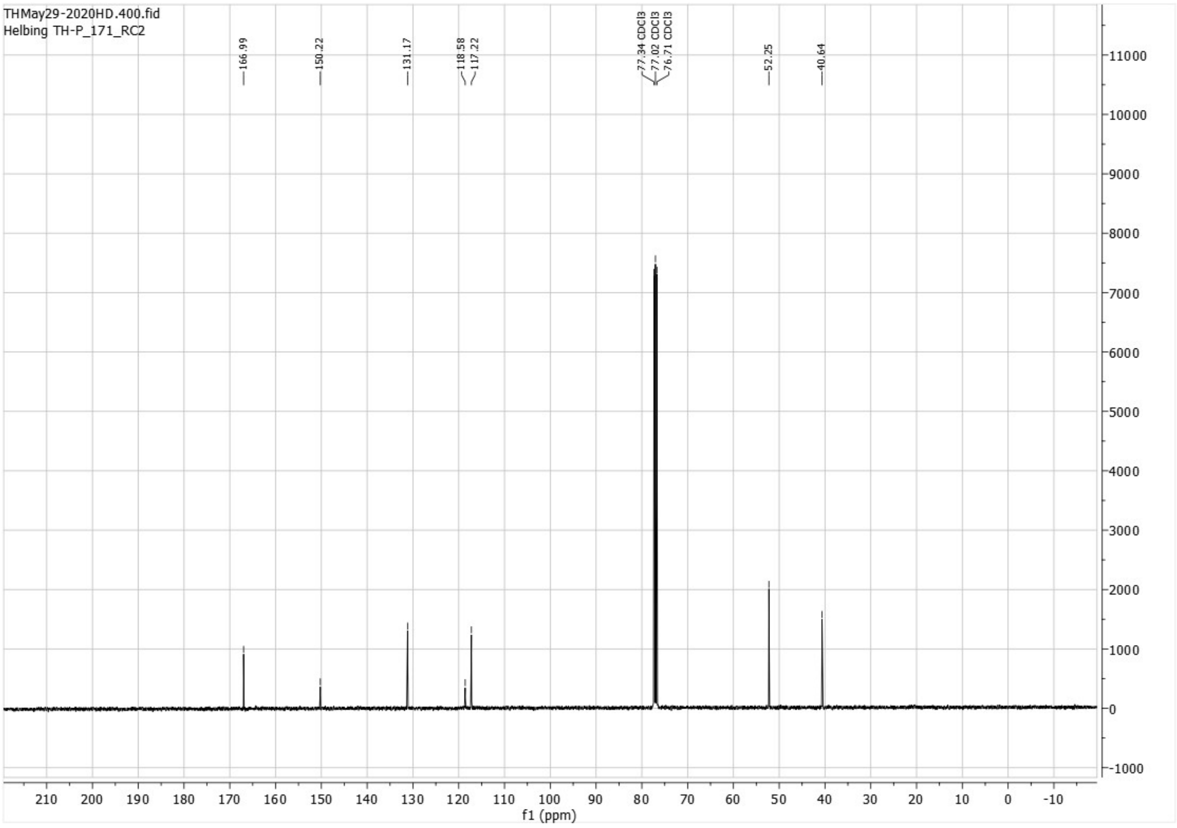
THMay29-2020HD.400.fid
Helbing TH-P_171_RC2
f1 (ppm)

Compound 3B

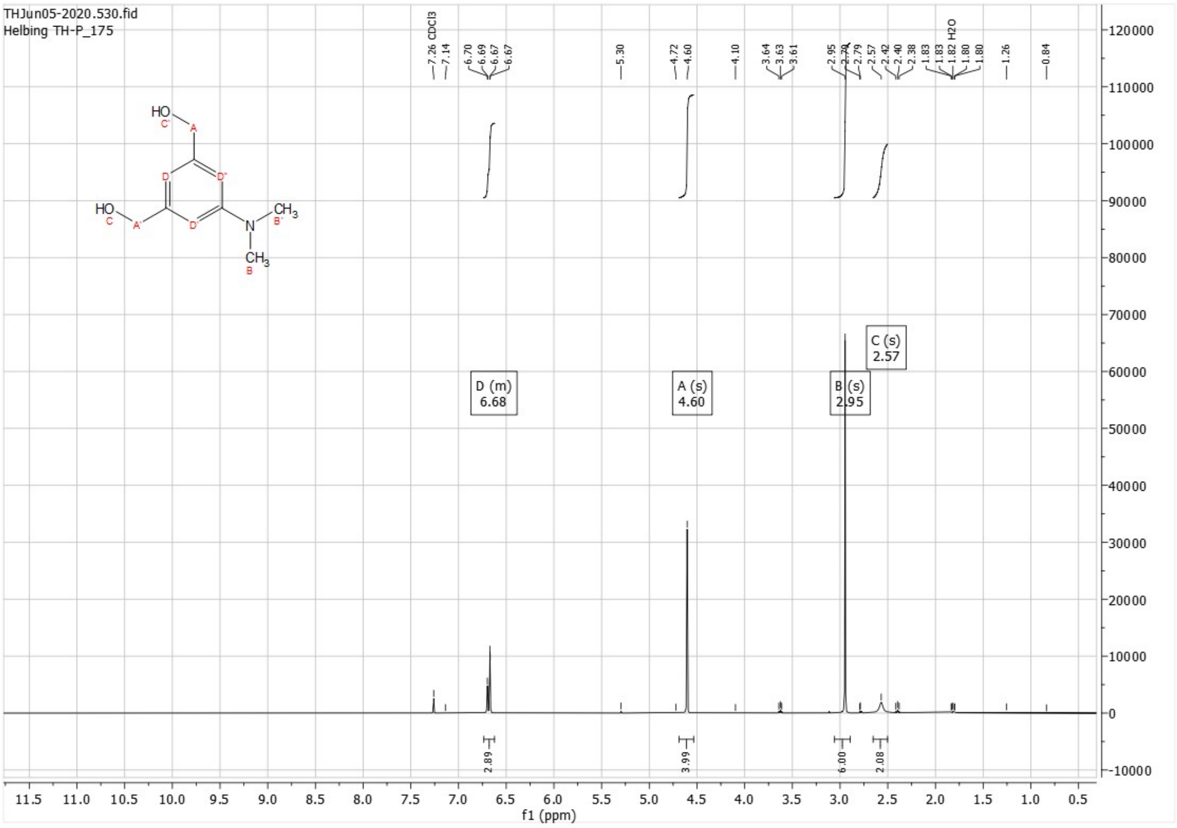
THJun05-2020.530.fid
Helbing TH-P_175
D (m)
6.68
A (s)
4.60
B (s)
2.95
C (s)
2.57
f1 (ppm)

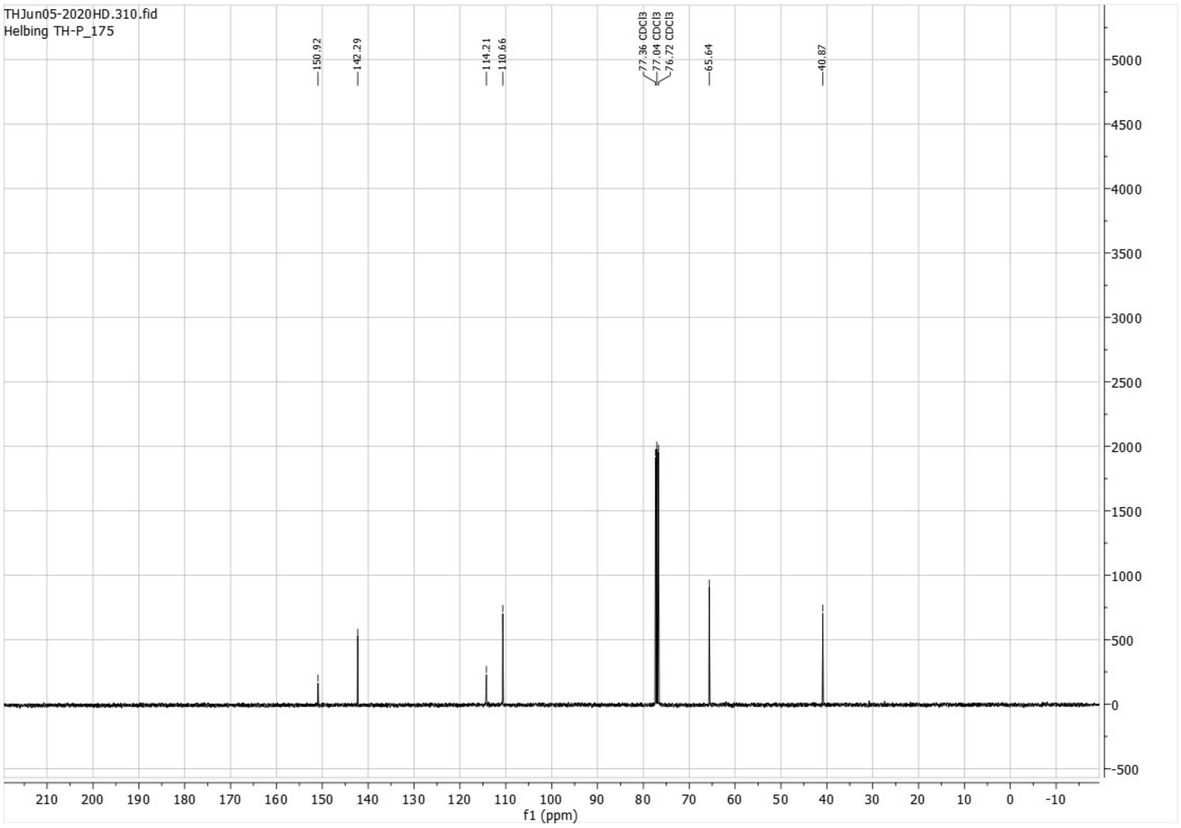
THJun05-2020HD.310.fid
Helbing TH-P_175
150.92
142.29
114.21
110.66
77.36 CDCl3
77.04 CDCl3
76.72 CDCl3
65.64
40.87
f1 (ppm)

Compound 3C

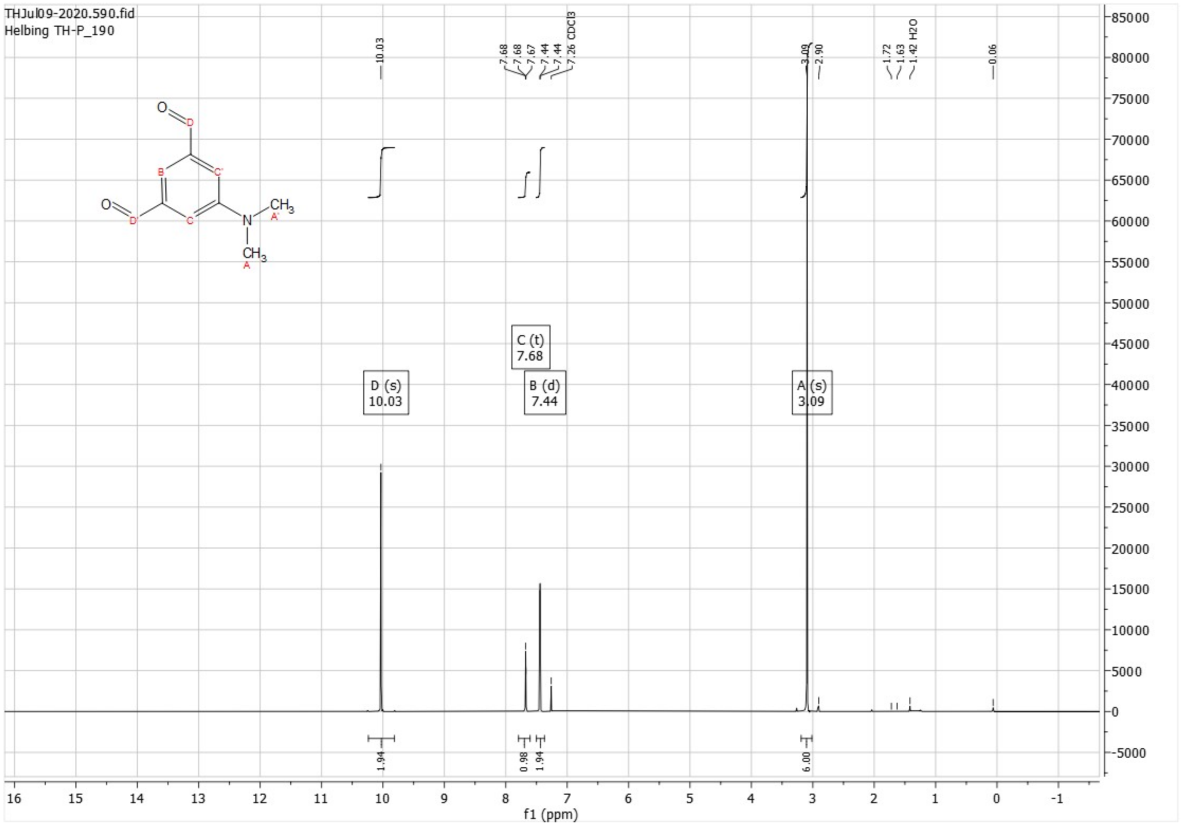
THJul09-2020.590.fid
Helbing TH-P_190
10.03
7.68
7.68
7.67
7.44
7.44
7.26 CDCl3
3.09
2.90
1.72
1.63
1.42 H2O
0.06
D (s)
10.03
C (t)
7.68
B (d)
7.44
A (s)
3.09
1.94
0.98
1.94
6.00
f1 (ppm)

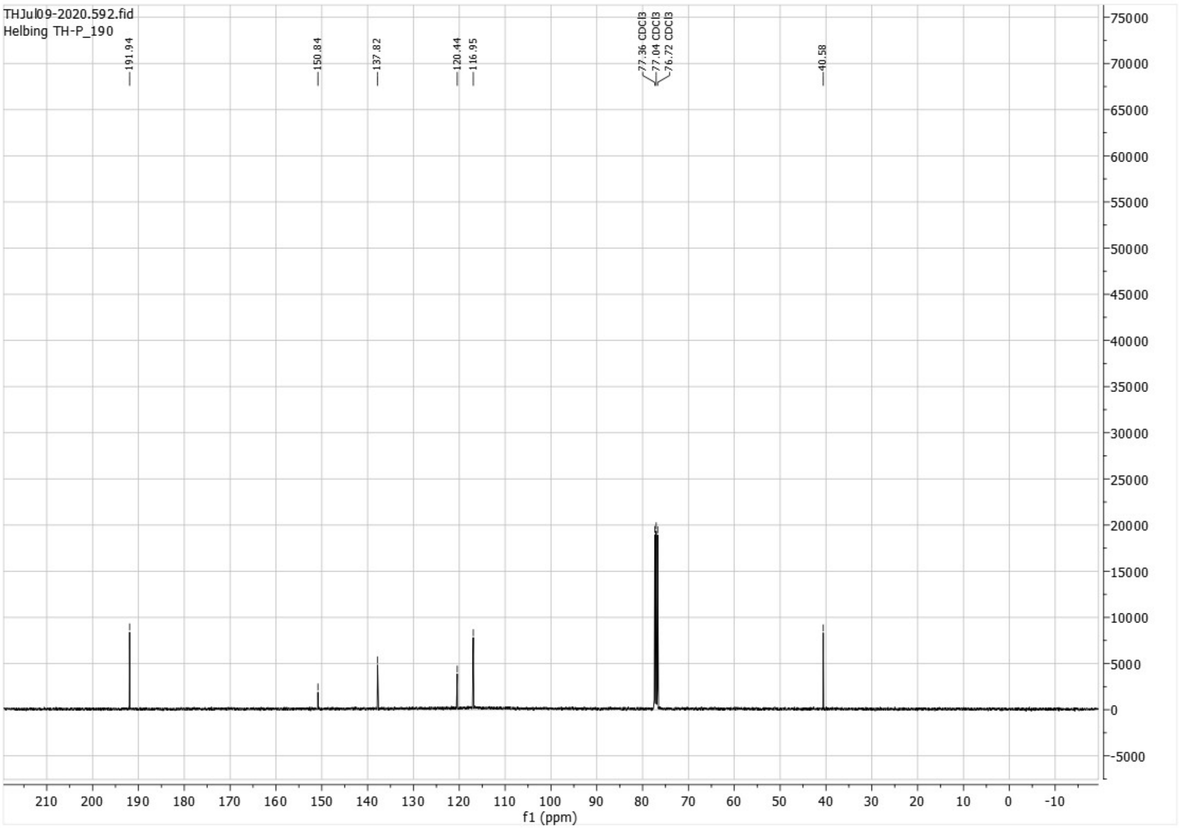
THJul09-2020.592.fid
Helbing TH-P_190
191.94
150.84
137.82
120.44
116.95
77.36 CDCl3
77.04 CDCl3
76.72 CDCl3
40.58
f1 (ppm)

Compound 3D

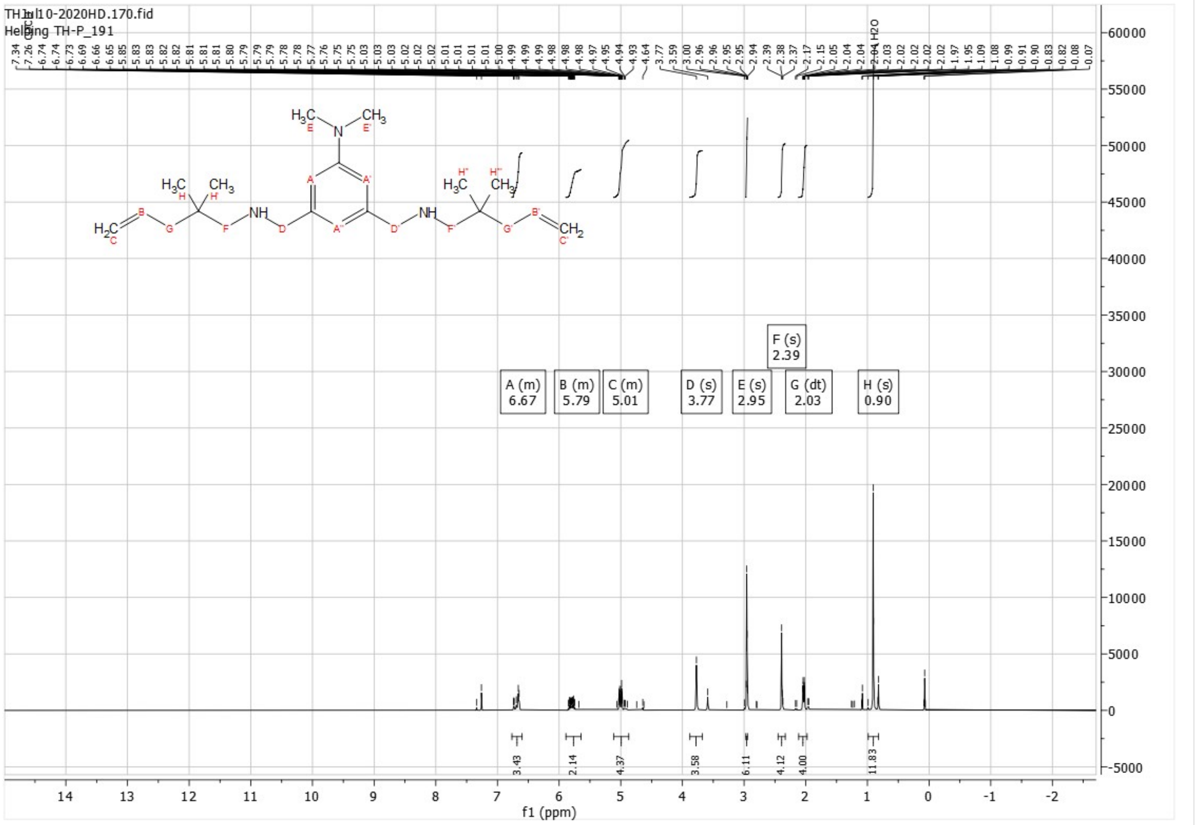
THJul10-2020HD.170.fid
Helbing TH-P_191
A (m)
6.67
B (m)
5.79
C (m)
5.01
D (s)
3.77
E (s)
2.95
F (s)
2.39
G (dt)
2.03
H (s)
0.90
H2O
3.43
2.14
4.37
3.58
6.11
4.12
4.00
11.83
f1 (ppm)

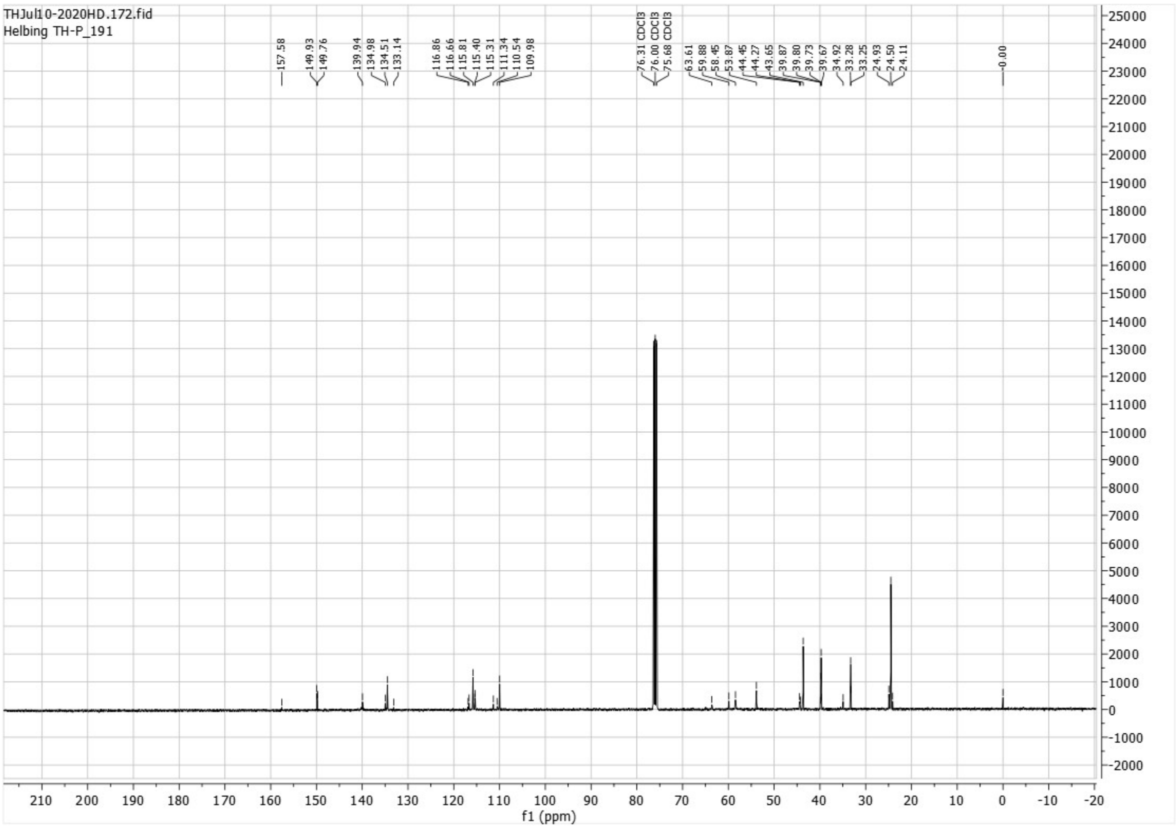
THJul10-2020HD.172.fid
Helbing TH-P_191
f1 (ppm)

Compound 3

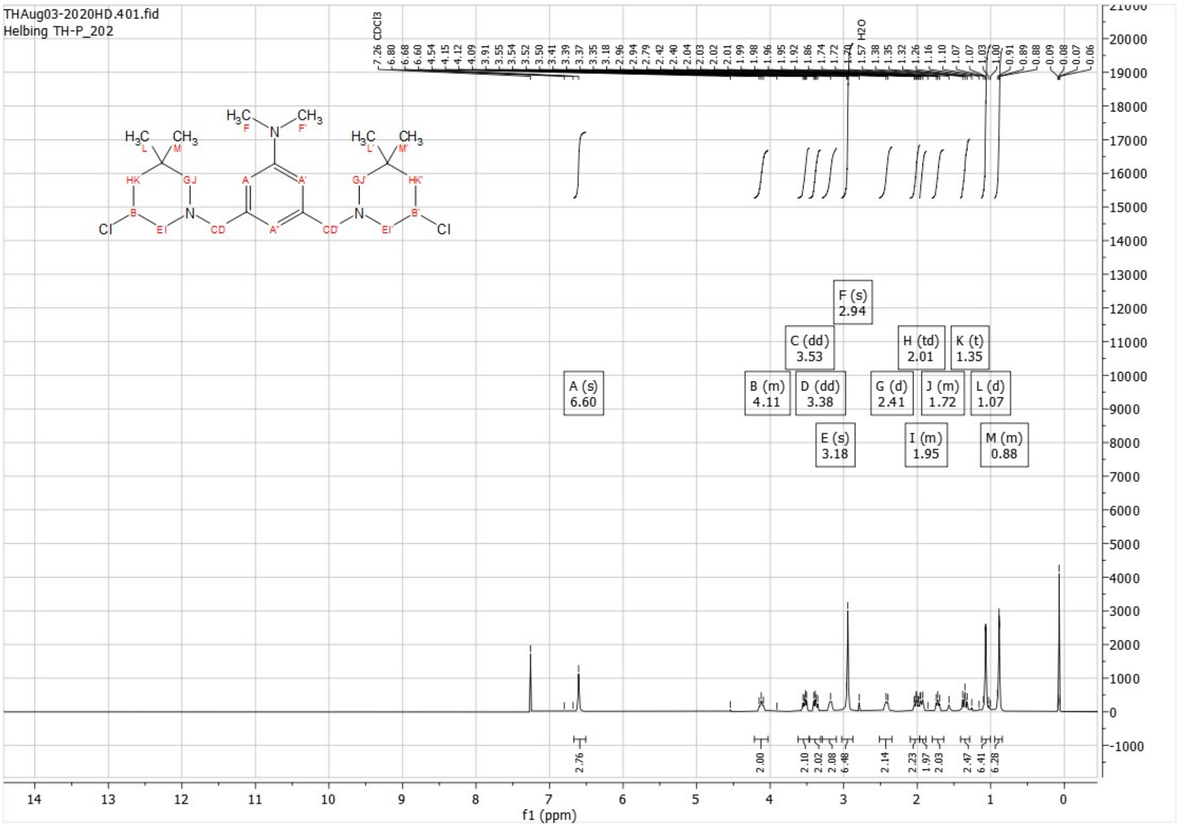
THAug03-2020HD.401.fid
Helbing TH-P_202
A (s)
6.60
B (m)
4.11
C (dd)
3.53
D (dd)
3.38
E (s)
3.18
F (s)
2.94
G (d)
2.41
H (td)
2.01
I (m)
1.95
J (m)
1.72
K (t)
1.35
L (d)
1.07
M (m)
0.88
f1 (ppm)

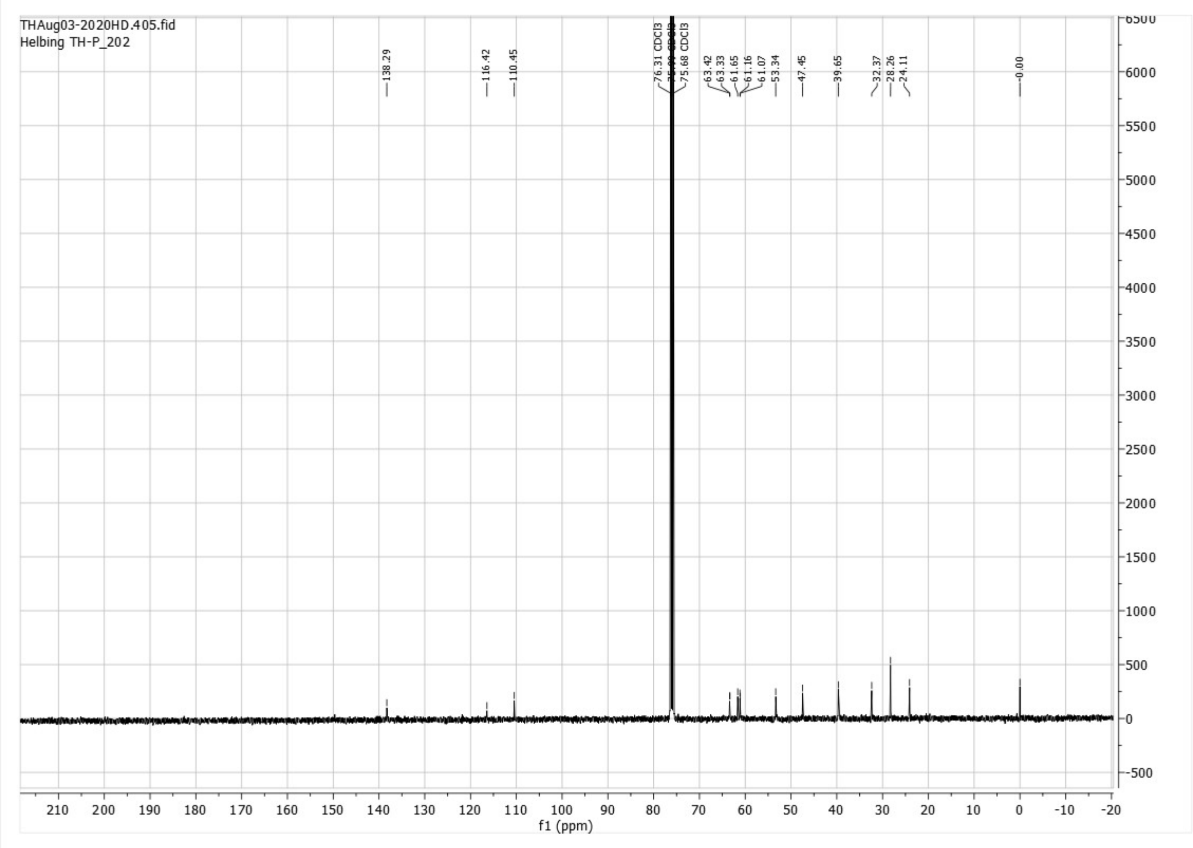

THAug03-2020HD.405.fid
Helbing TH-P_202
138.29
116.42
110.45
76.31 CDCl3
75.68 CDCl3
63.42
63.33
61.65
61.16
61.07
53.34
47.45
39.65
32.37
28.26
24.11
0.00
f1 (ppm)

Compound 5A

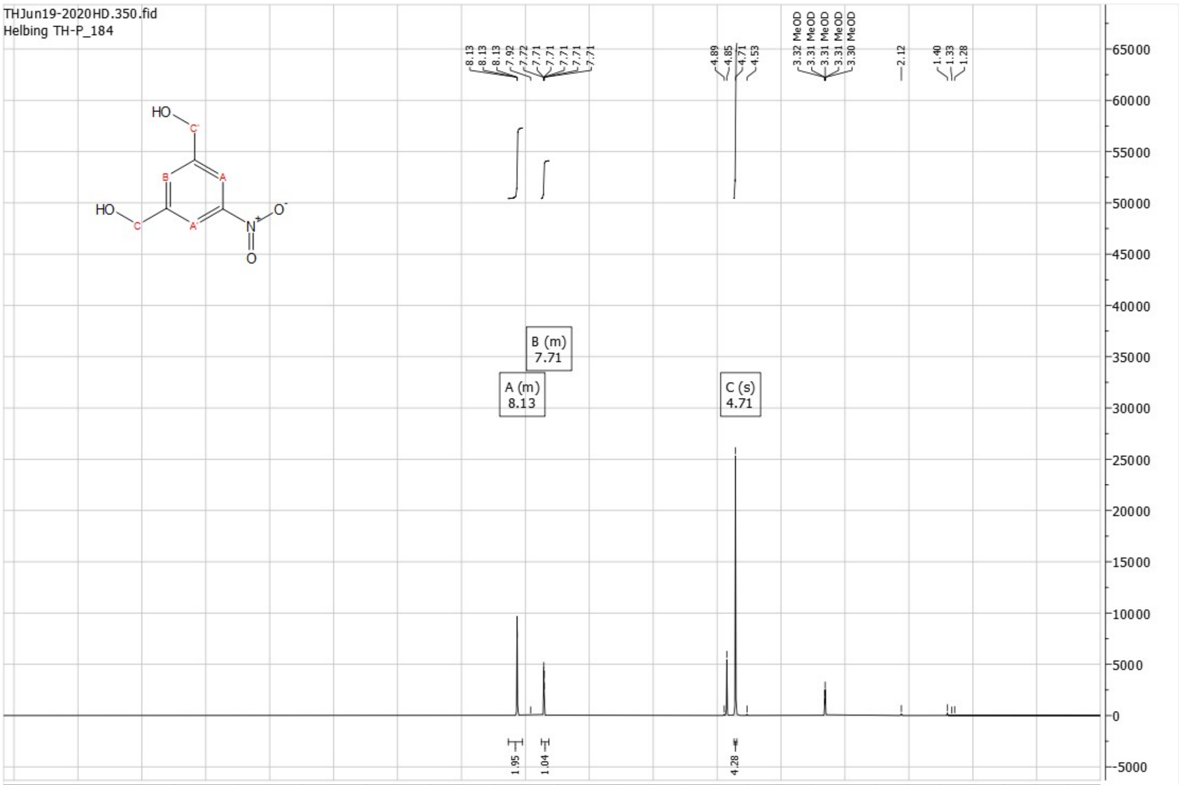

THJun19-2020HD.350.fid
Helbing TH-P_184
8.13
8.13
8.13
7.92
7.72
7.71
7.71
7.71
7.71
7.71
4.89
4.85
4.71
4.53
3.32 MeOD
3.31 MeOD
3.31 MeOD
3.31 MeOD
3.30 MeOD
2.12
1.40
1.33
1.28
B (m)
7.71
A (m)
8.13
C (s)
4.71
1.95
1.04
4.28
f1 (ppm)

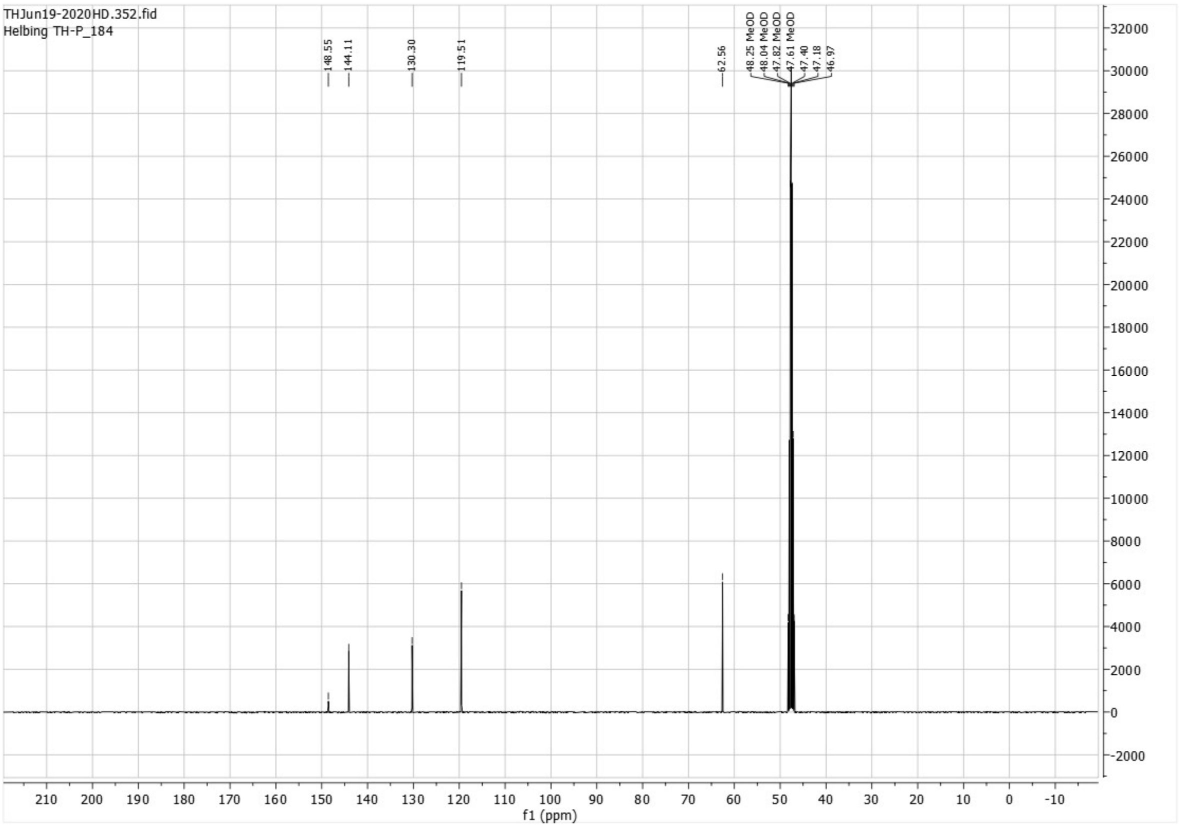
THJun19-2020HD.352.fid
Helbing TH-P_184
148.55
144.11
130.30
119.51
62.56
48.25 MeOD
48.04 MeOD
47.82 MeOD
47.61 MeOD
47.40
47.18
46.97
f1 (ppm)

Compound 5B

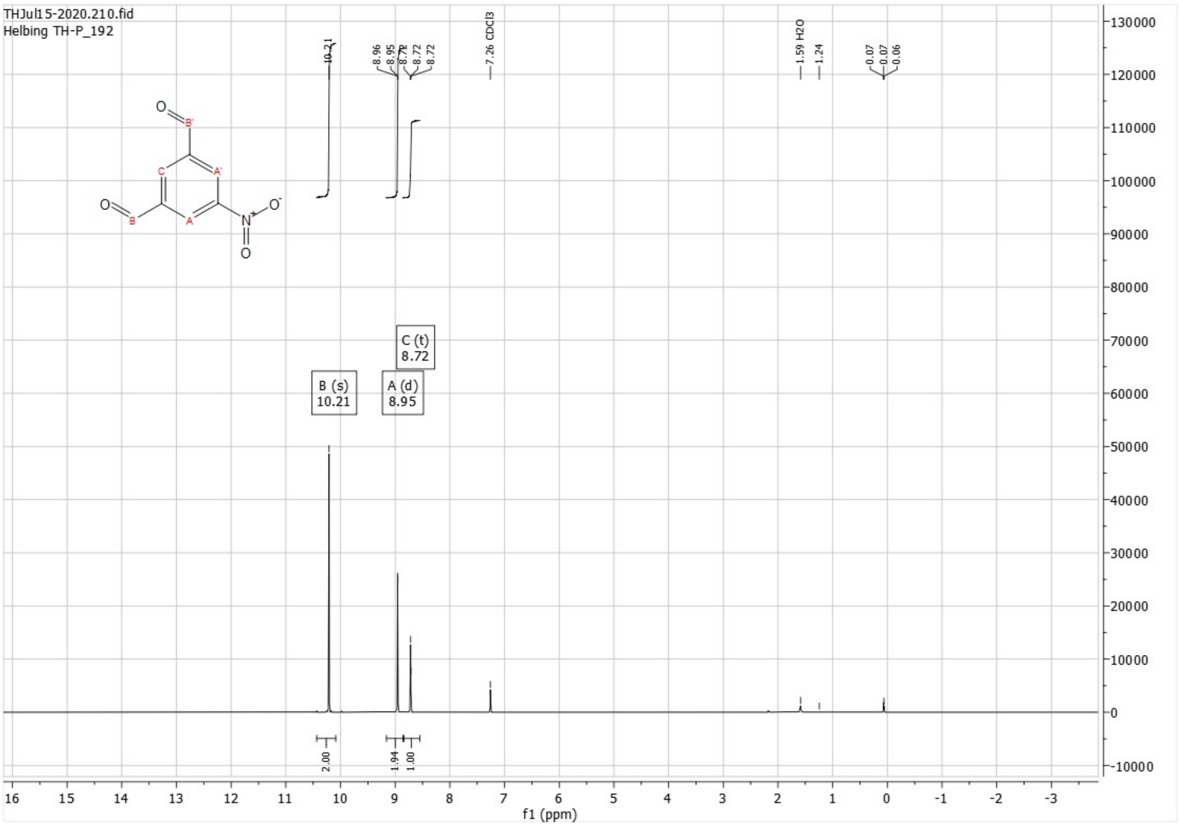
THJul15-2020.210.fid
Helbing TH-P_192
10.21
8.96
8.95
8.72
8.72
8.72
7.26 CDCl3
1.59 H2O
1.24
0.07
0.07
0.06
C (t)
8.72
B (s)
10.21
A (d)
8.95
2.00
1.94
1.00
f1 (ppm)

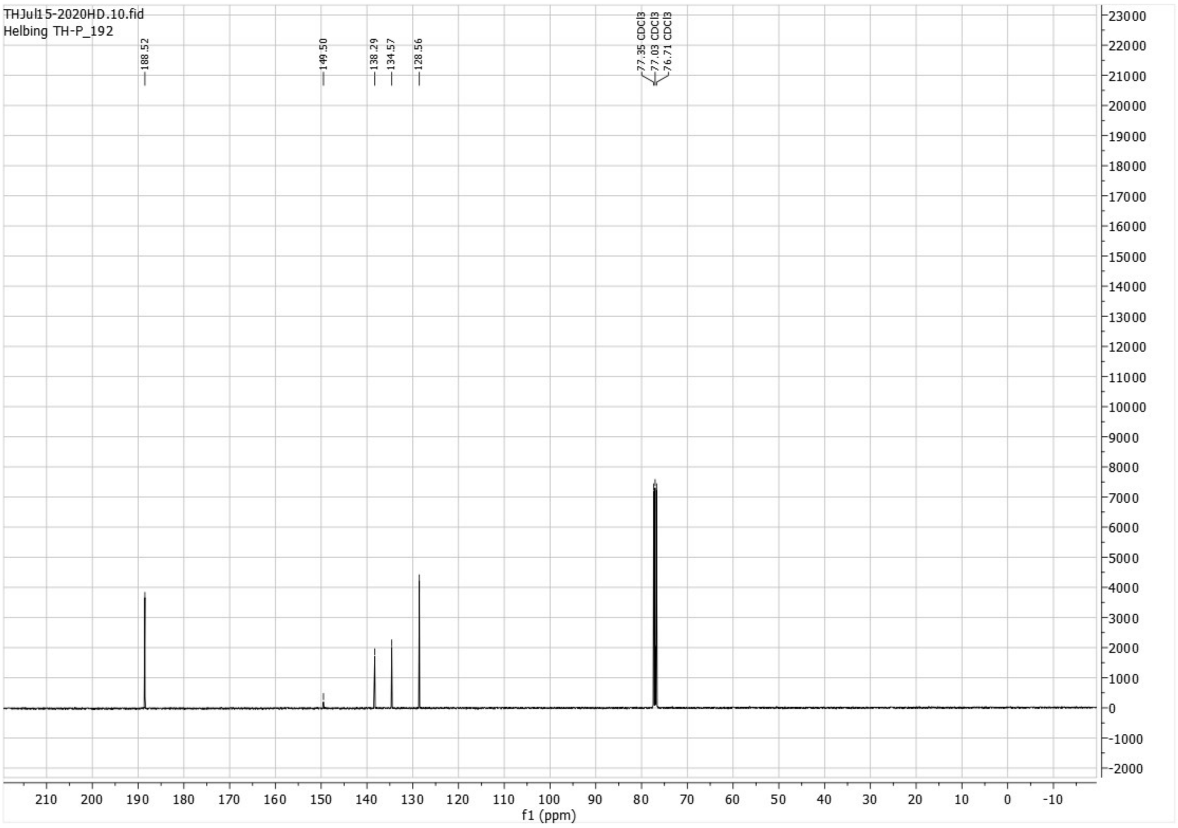
THJul15-2020HD.10.fid
Helbing TH-P_192
188.52
149.50
138.29
134.57
128.56
77.35 CDCl3
77.03 CDCl3
76.71 CDCl3
f1 (ppm)

Compound 5C

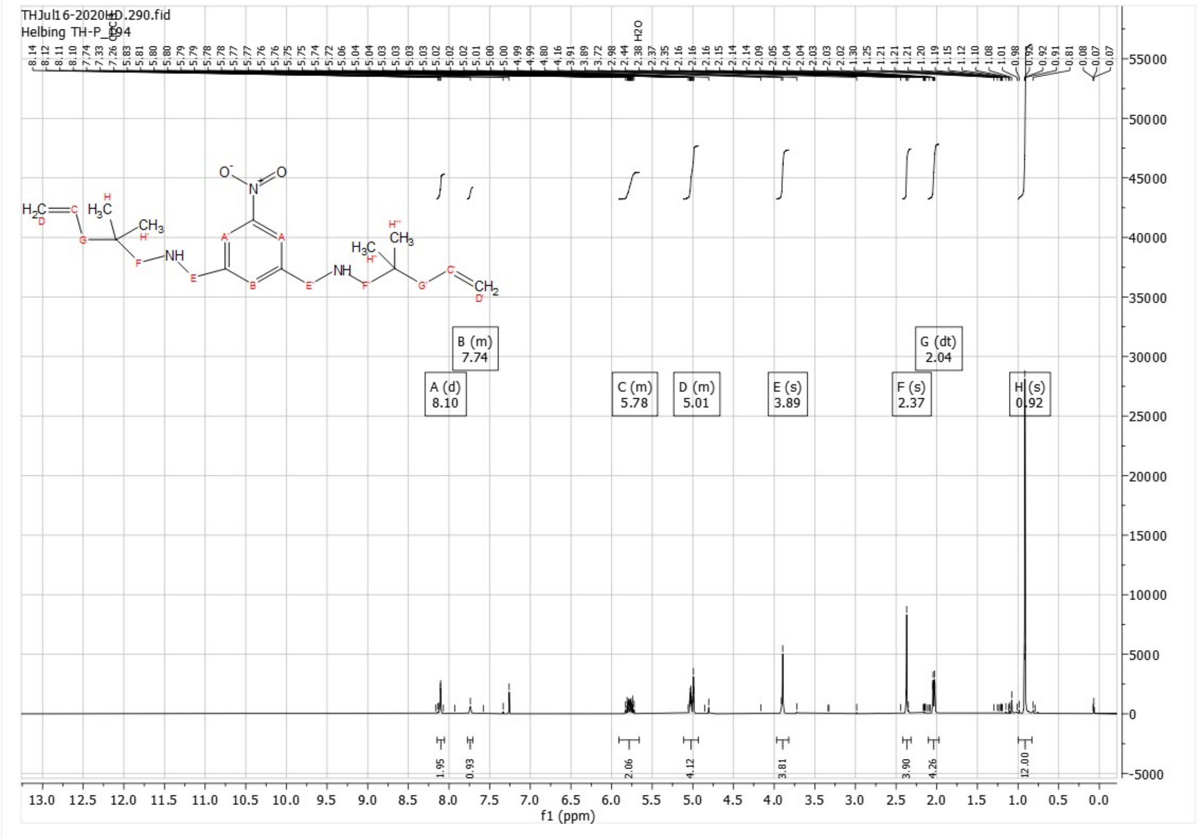
THJul16-2020HD.290.fid
Helbing TH-P_194
H2O
A (d)
8.10
B (m)
7.74
C (m)
5.78
D (m)
5.01
E (s)
3.89
F (s)
2.37
G (dt)
2.04
H (s)
0.92
1.95
0.93
2.06
4.12
3.81
3.90
4.26
12.00
f1 (ppm)

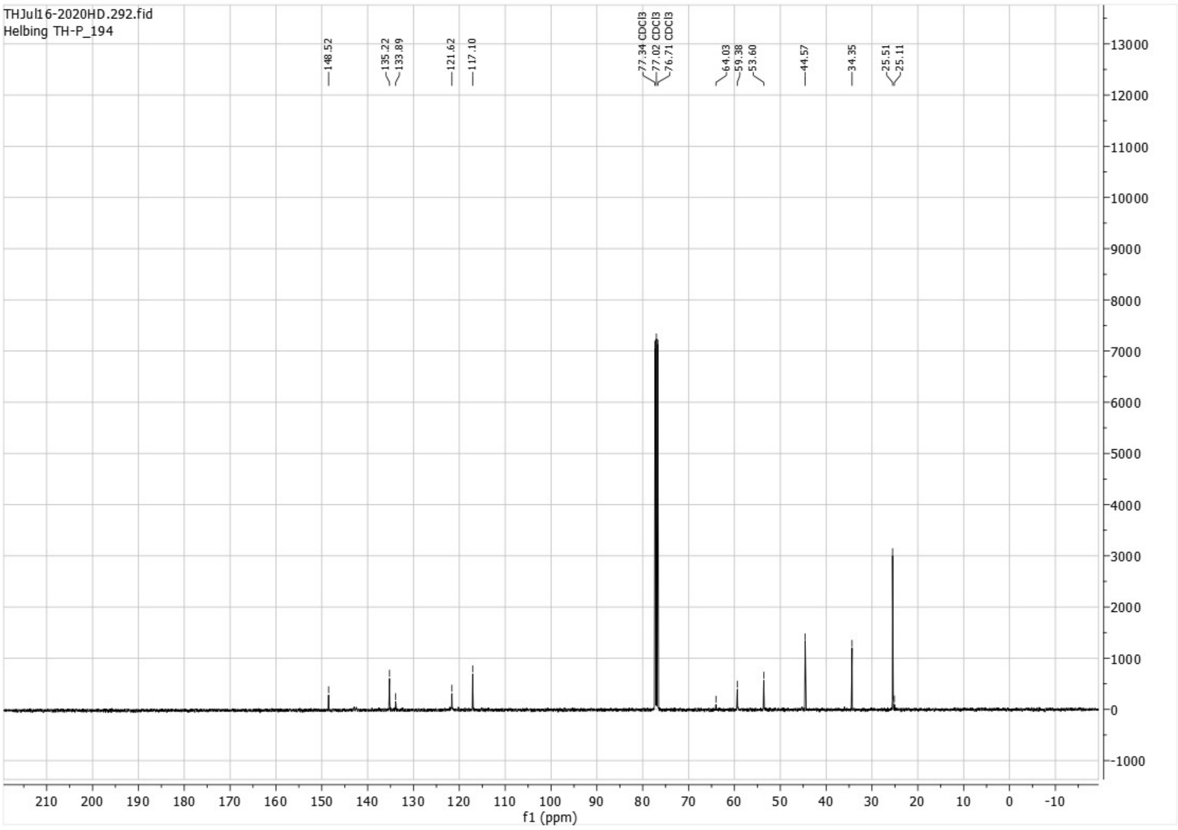

Compound 5D

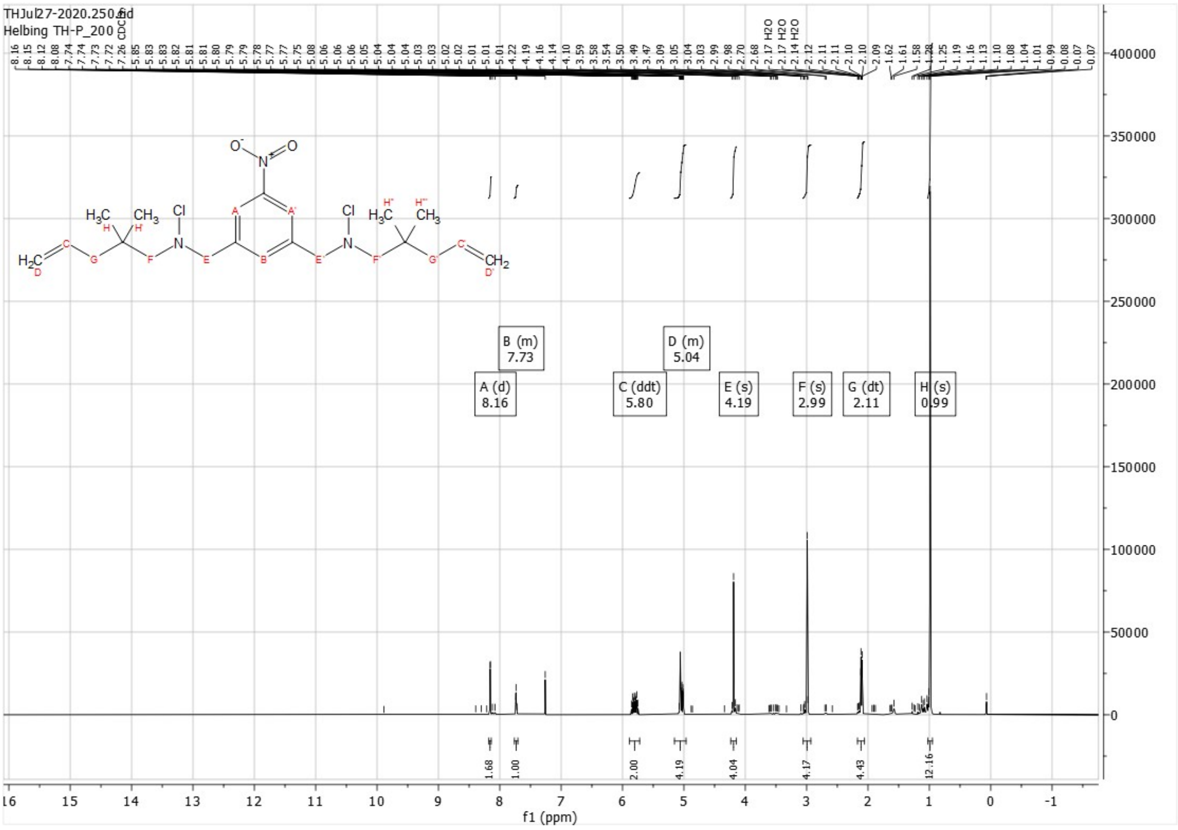

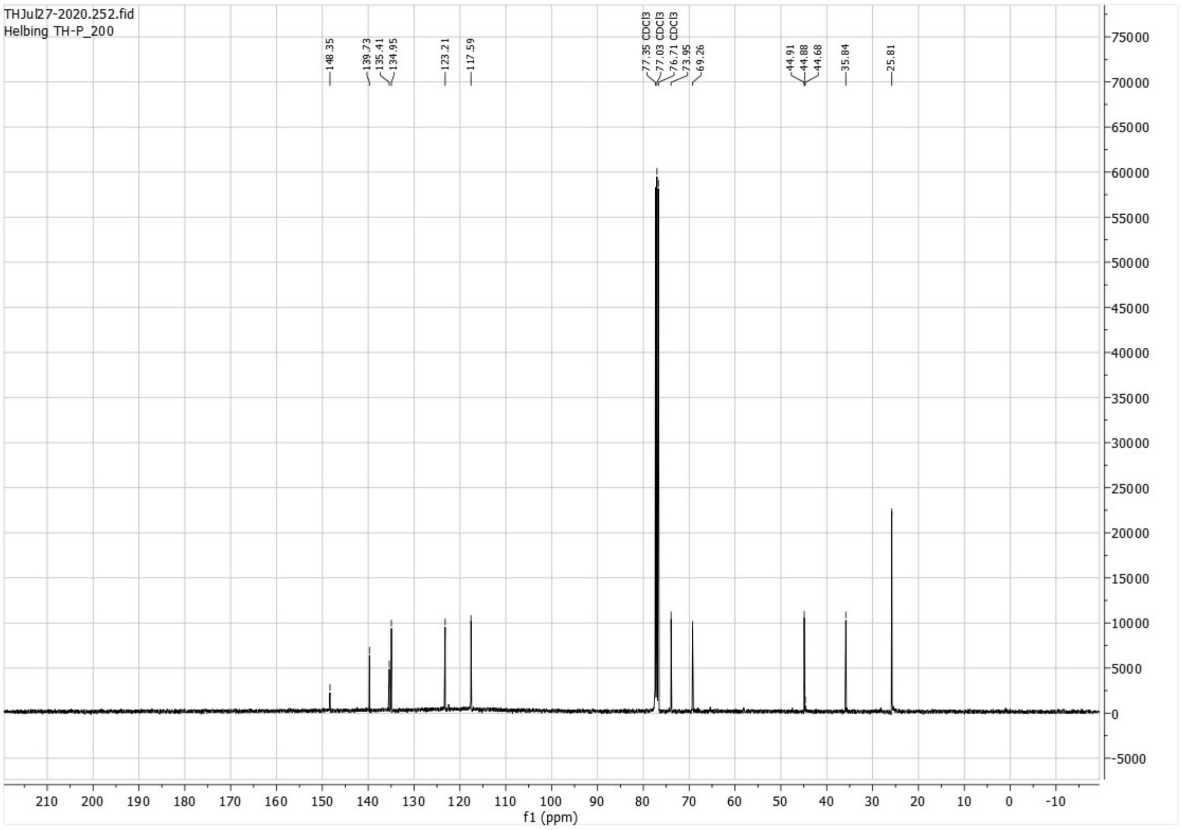

THJul27-2020.252.fid
Helbing TH-P_200
148.35
139.73
135.41
134.95
123.21
117.59
77.35 CDCl3
77.03 CDCl3
76.71 CDCl3
73.95
69.26
44.91
44.88
44.68
35.84
25.81
f1 (ppm)

Compound 5

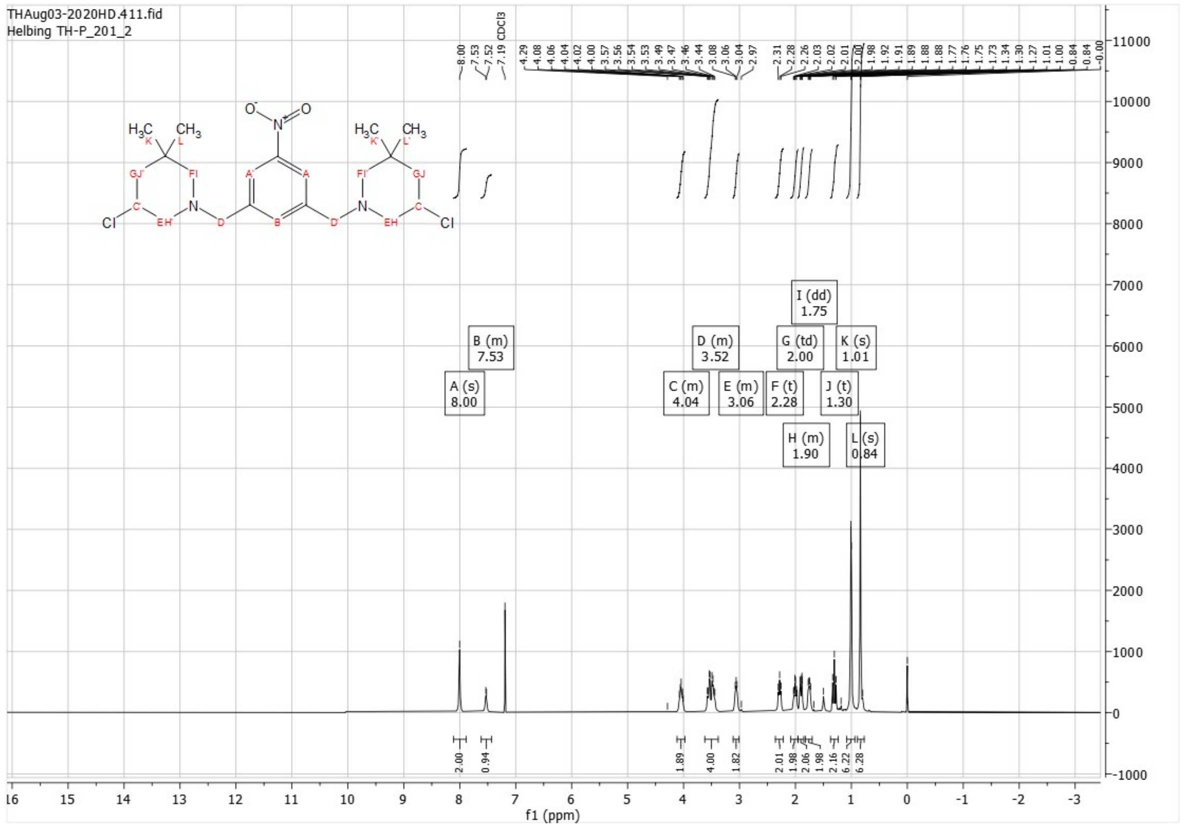

THAug03-2020HD.411.fid
Helbing TH-P_201_2
A (s)
8.00
B (m)
7.53
C (m)
4.04
D (m)
3.52
E (m)
3.06
F (t)
2.28
G (td)
2.00
H (m)
1.90
I (dd)
1.75
J (t)
1.30
K (s)
1.01
L (s)
0.84
f1 (ppm)

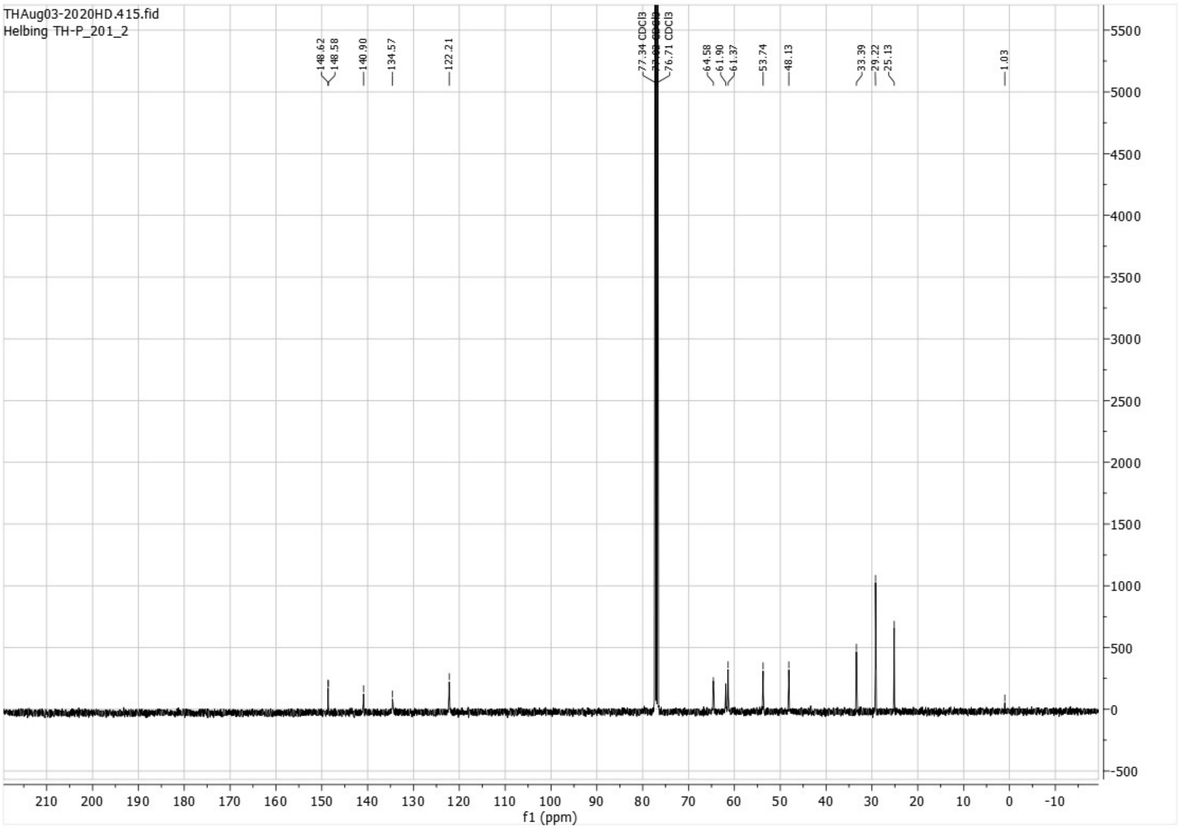
THAug03-2020HD.415.fid
Helbing TH-P_201_2
148.62
148.58
140.90
134.57
122.21
77.34 CDCl3
76.71 CDCl3
64.58
61.90
61.37
53.74
48.13
33.39
29.22
25.13
1.03
f1 (ppm)

Compound 7A

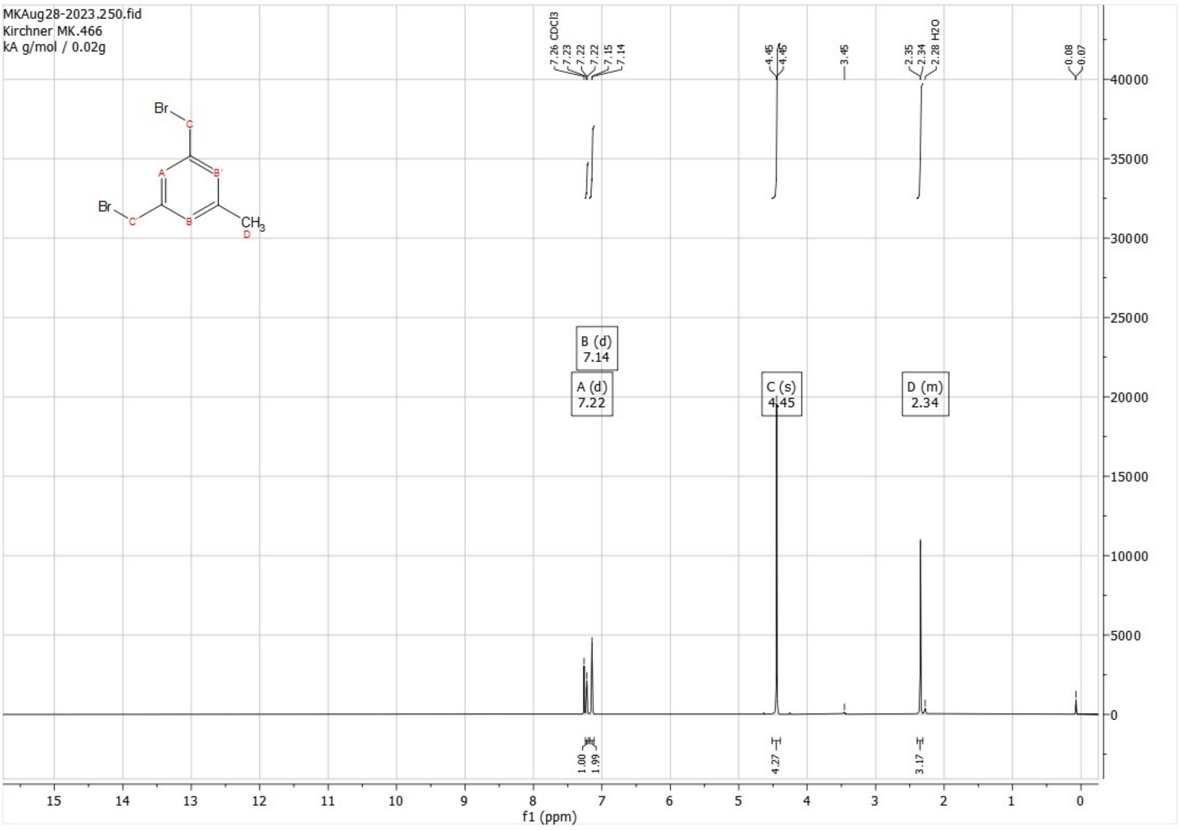
MKAug28-2023.250.fid
Kirchner MK.466
kA g/mol / 0.02g
7.26 CDCl3
7.23
7.22
7.22
7.15
7.14
4.45
4.45
3.45
2.35
2.34
2.28 H2O
0.08
0.07
B (d)
7.14
A (d)
7.22
C (s)
4.45
D (m)
2.34
1.00
1.99
4.27
3.17
f1 (ppm)

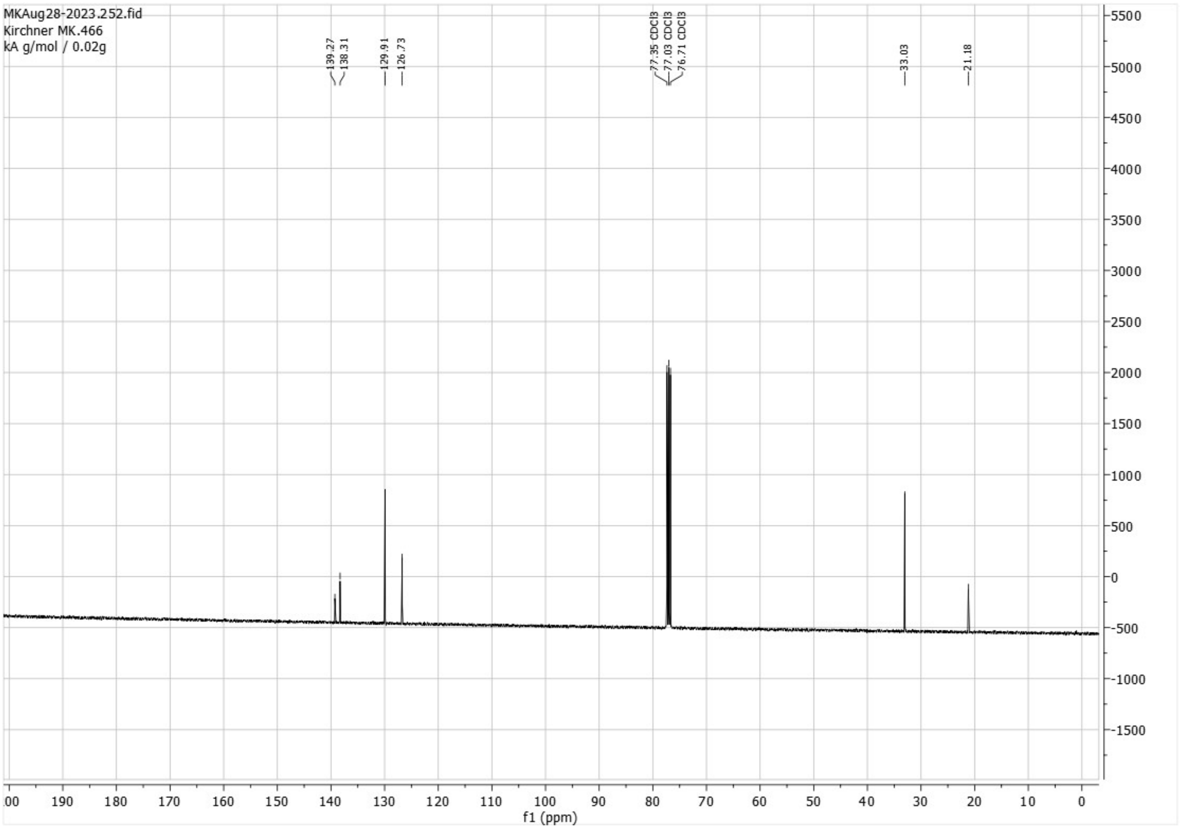
MKAug28-2023.252.fid
Kirchner MK.466
kA g/mol / 0.02g
139.27
138.31
129.91
126.73
77.35 CDCl3
77.03 CDCl3
76.71 CDCl3
33.03
21.18
f1 (ppm)

Compound 7B

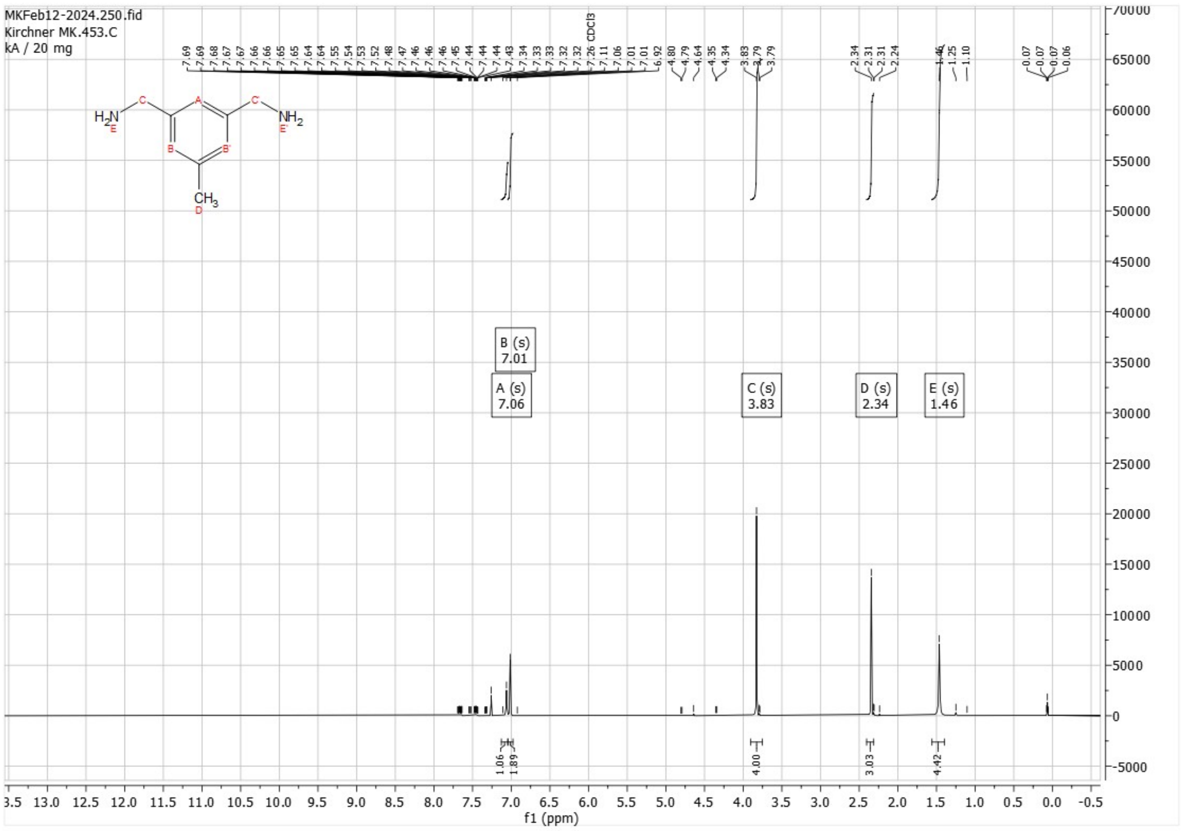
MKFeb12-2024.250.fid
Kirchner MK.453.C
kA / 20 mg
B (s)
7.01
A (s)
7.06
C (s)
3.83
D (s)
2.34
E (s)
1.46
1.06
1.89
4.00
3.03
4.42
f1 (ppm)

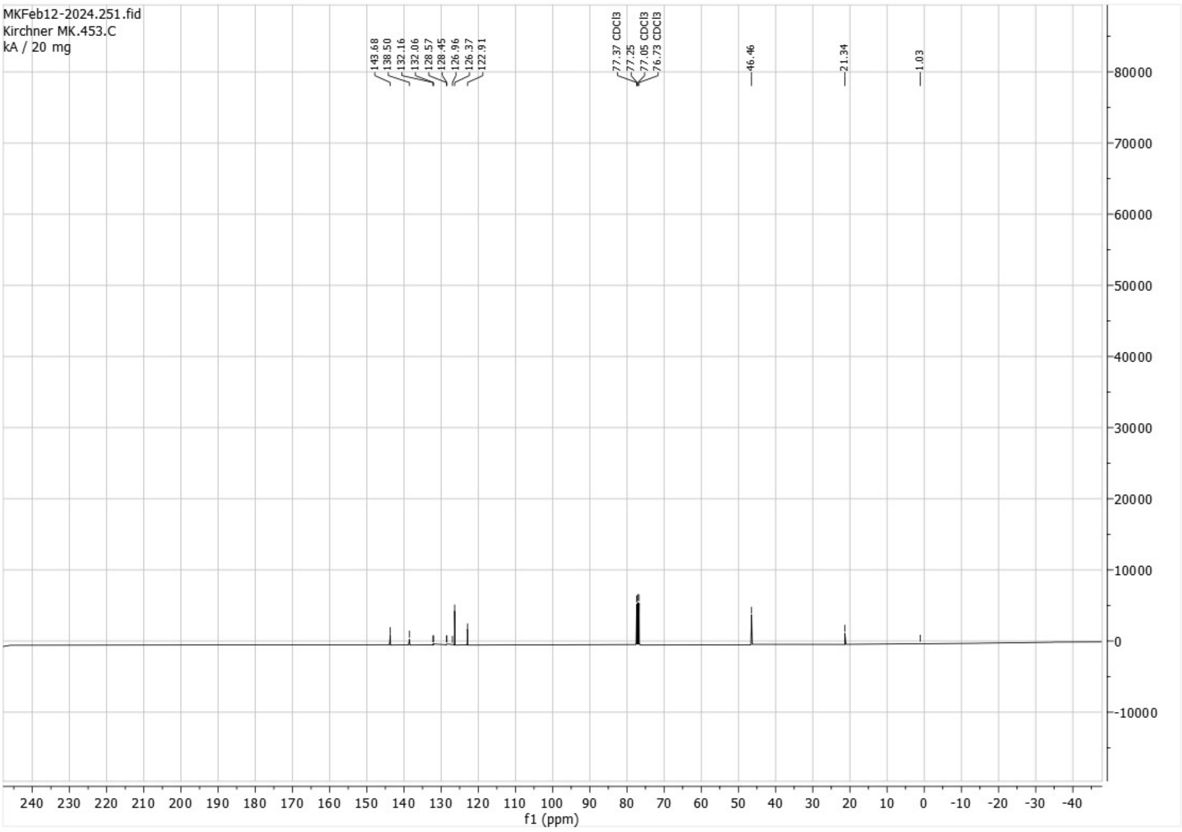

MKFeb12-2024.251.fid
Kirchner MK.453.C
kA / 20 mg
143.68
138.50
132.16
132.06
128.57
128.45
126.96
126.37
122.91
77.37 CDCl3
77.25
77.05 CDCl3
76.73 CDCl3
46.46
21.34
1.03
f1 (ppm)

Compound 7C

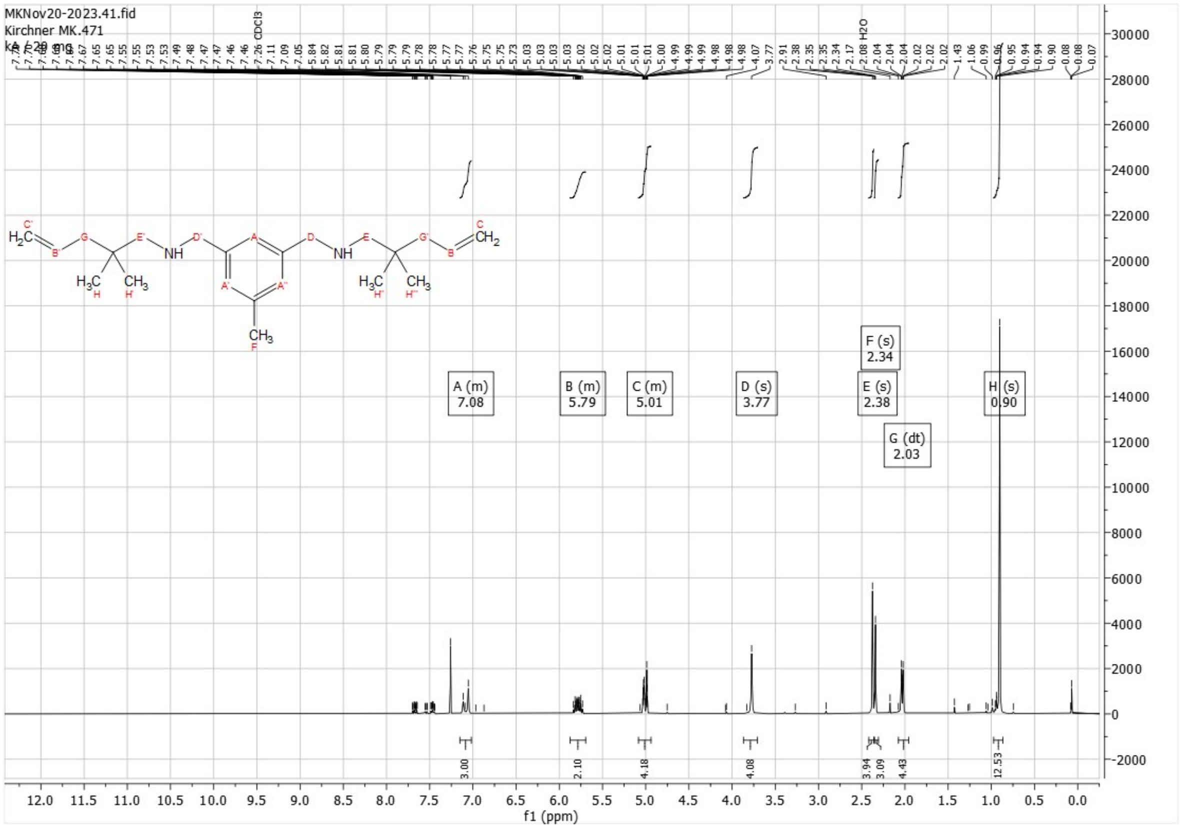

MKNov20-2023.41.fid
Kirchner MK.471
kA / 20 mg
A (m)
7.08
B (m)
5.79
C (m)
5.01
D (s)
3.77
E (s)
2.38
F (s)
2.34
G (dt)
2.03
H (s)
0.90
3.00
2.10
4.18
4.08
3.94
3.09
4.43
12.53
f1 (ppm)

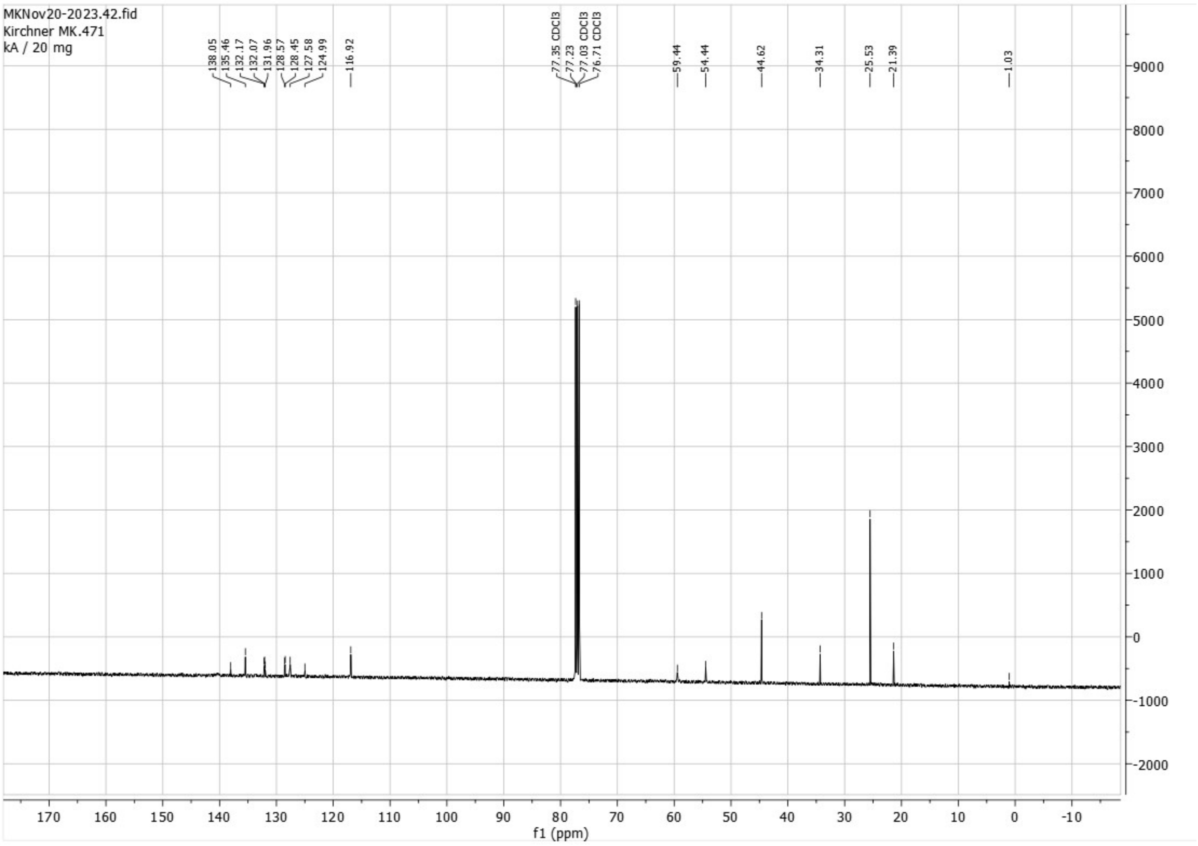
MKNov20-2023.42.fid
Kirchner MK.471
kA / 20 mg
138.05
135.46
132.17
132.07
131.96
128.57
128.45
127.58
124.99
116.92
77.35 CDCl3
77.23
77.03 CDCl3
76.71 CDCl3
59.44
54.44
44.62
34.31
25.53
21.39
1.03
f1 (ppm)

Compound 7

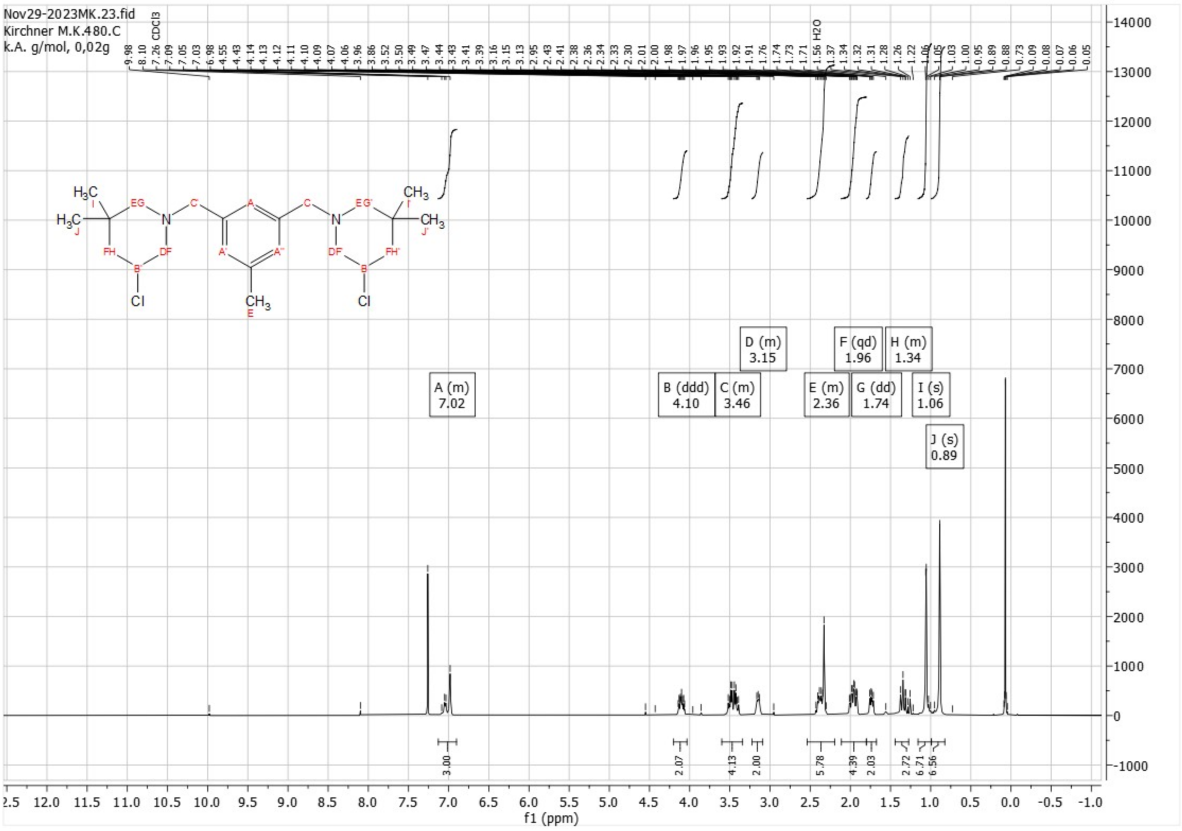
Nov29-2023MK.23.fid
Kirchner M.K.480.C
k.A. g/mol, 0,02g
A (m)
7.02
B (ddd)
4.10
C (m)
3.46
D (m)
3.15
E (m)
2.36
F (qd)
1.96
G (dd)
1.74
H (m)
1.34
I (s)
1.06
J (s)
0.89
3.00
2.07
4.13
2.00
5.78
4.39
2.03
2.72
6.71
6.56
f1 (ppm)

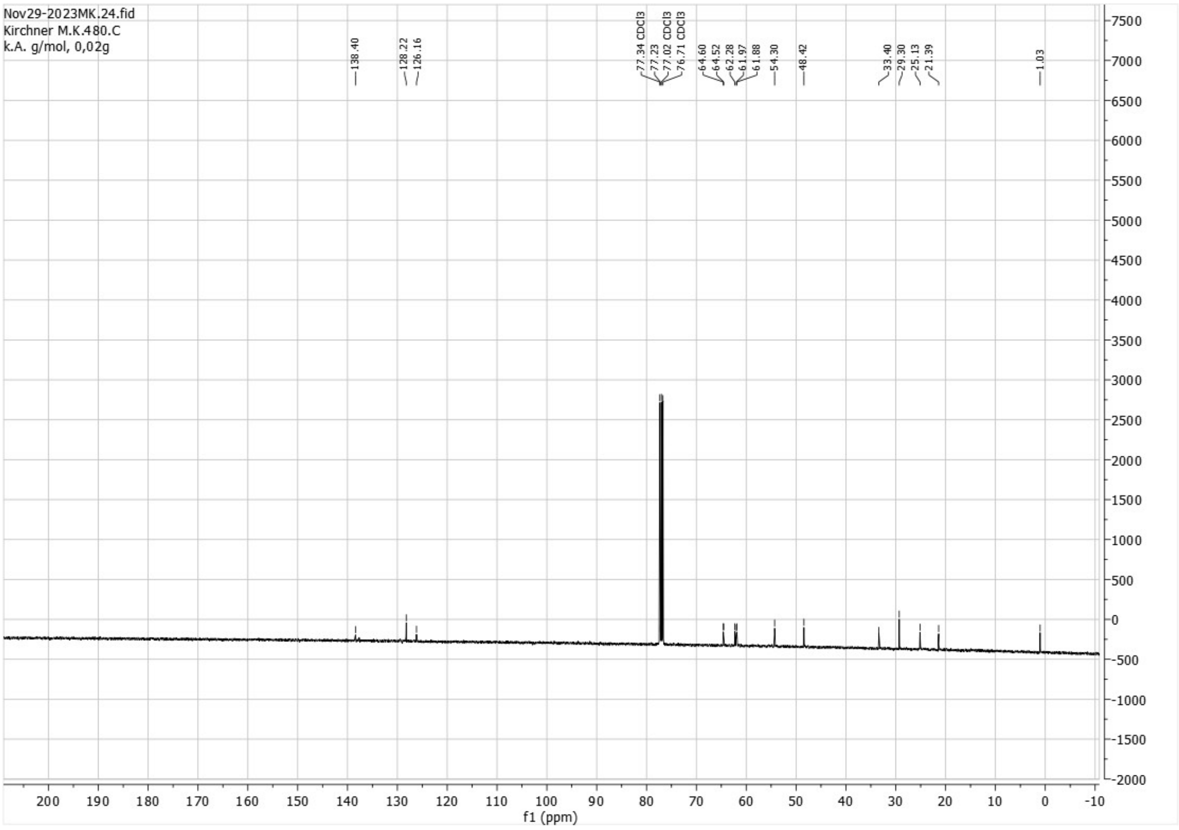
Nov29-2023MK.24.fid
Kirchner M.K.480.C
k.A. g/mol, 0,02g
f1 (ppm)

Compound 9A

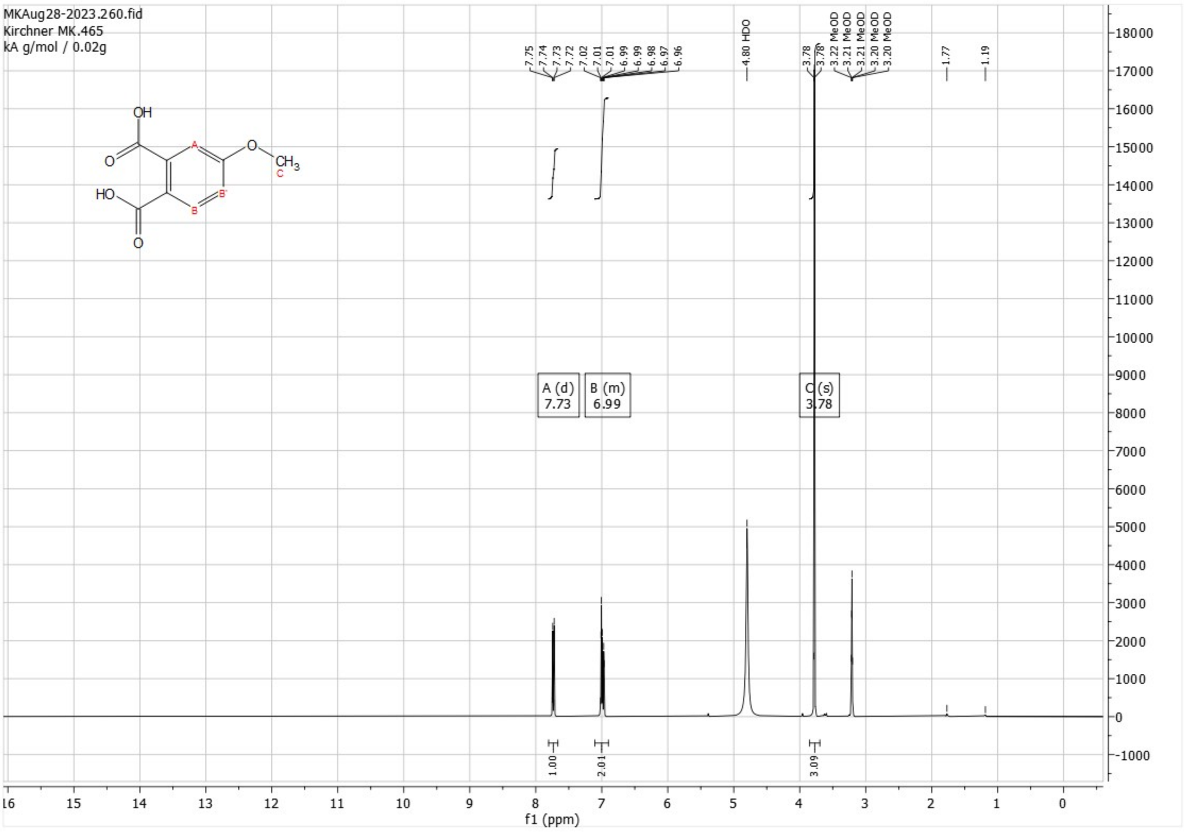
MKAug28-2023.260.fid
Kirchner MK.465
kA g/mol / 0.02g
A (d) 7.73
B (m) 6.99
C (s) 3.78
f1 (ppm)

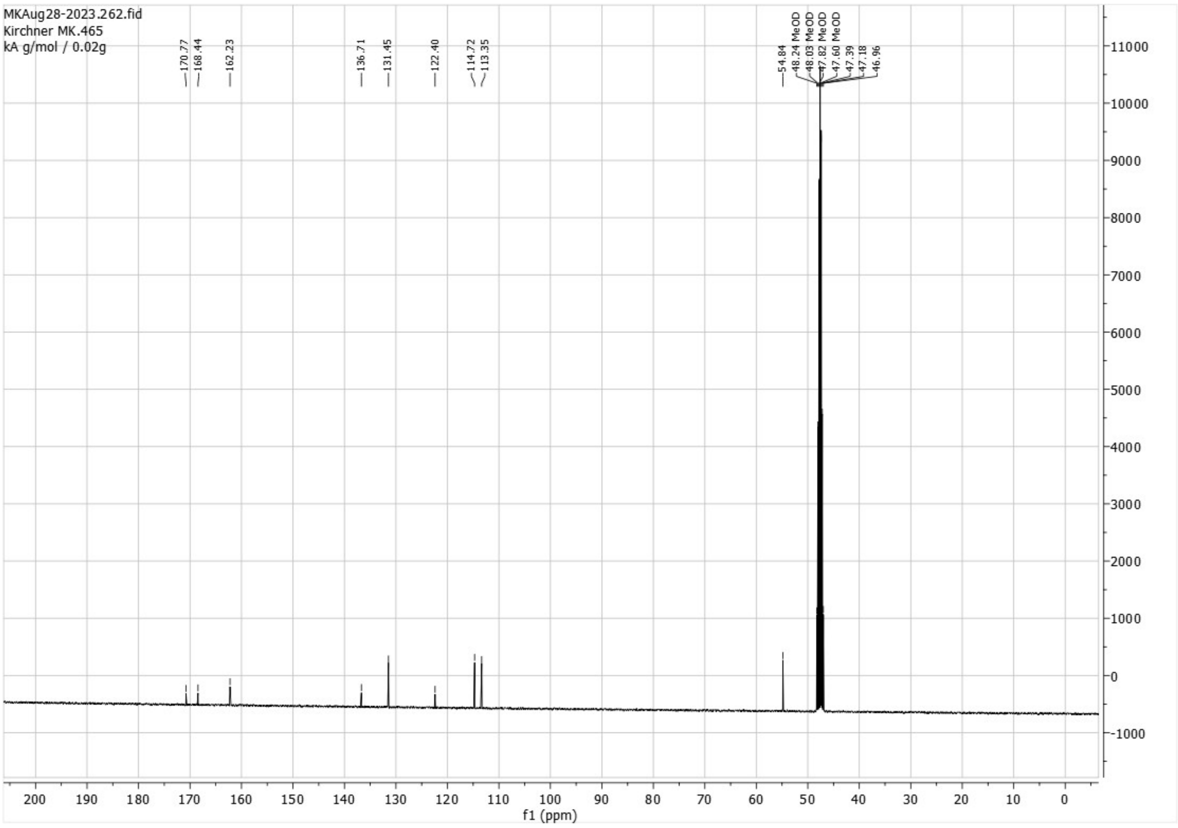
MKAug28-2023.262.fid
Kirchner MK.465
kA g/mol / 0.02g
f1 (ppm)

Compound 9B

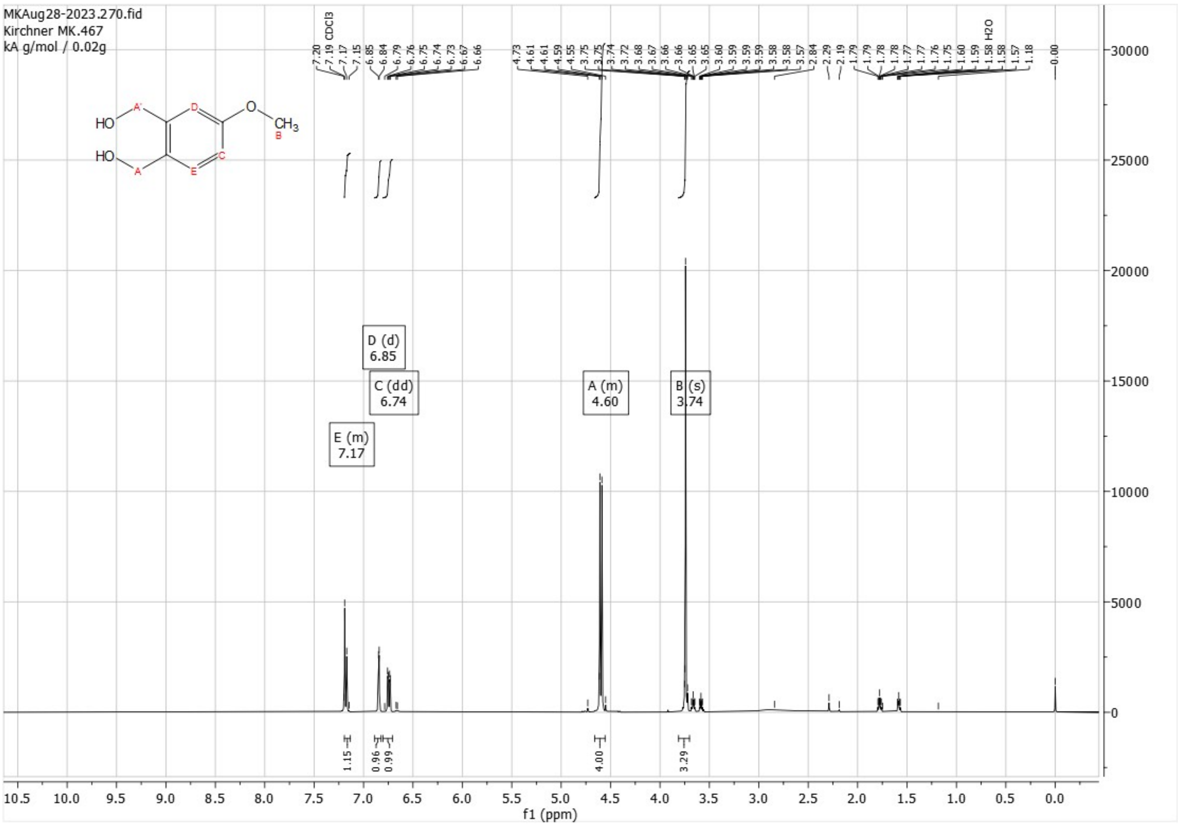
MKAug28-2023.270.fid
Kirchner MK.467
kA g/mol / 0.02g
D (d) 6.85
C (dd) 6.74
E (m) 7.17
A (m) 4.60
B (s) 3.74
f1 (ppm)

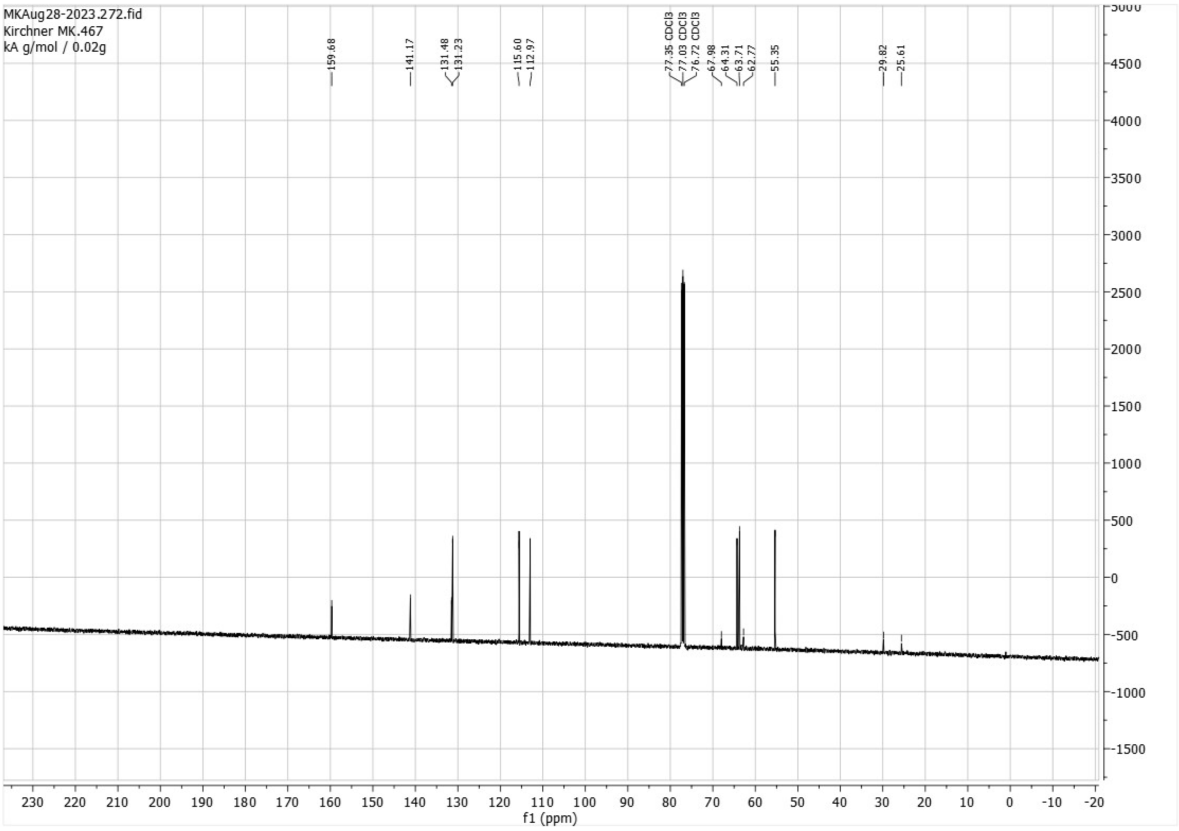
MKAug28-2023.272.fid
Kirchner MK.467
kA g/mol / 0.02g
159.68
141.17
131.48
131.23
115.60
112.97
77.35 CDCl3
77.03 CDCl3
76.72 CDCl3
67.98
64.31
63.71
62.77
55.35
29.82
25.61
f1 (ppm)

Compound 9C

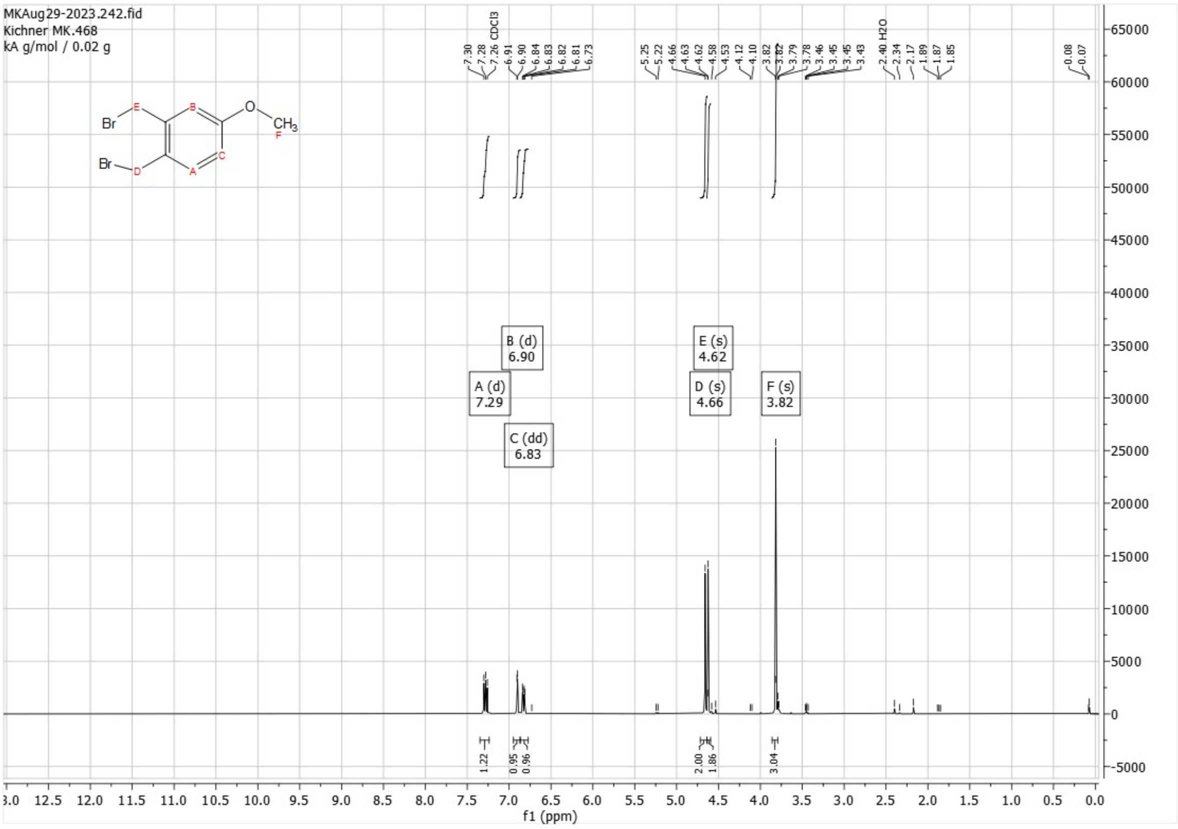
MKAug29-2023.242.fid
Kichner MK.468
kA g/mol / 0.02 g
Br
Br
O
CH3
A (d)
7.29
B (d)
6.90
C (dd)
6.83
D (s)
4.66
E (s)
4.62
F (s)
3.82
1.22
0.95
0.96
2.00
1.86
3.04
f1 (ppm)

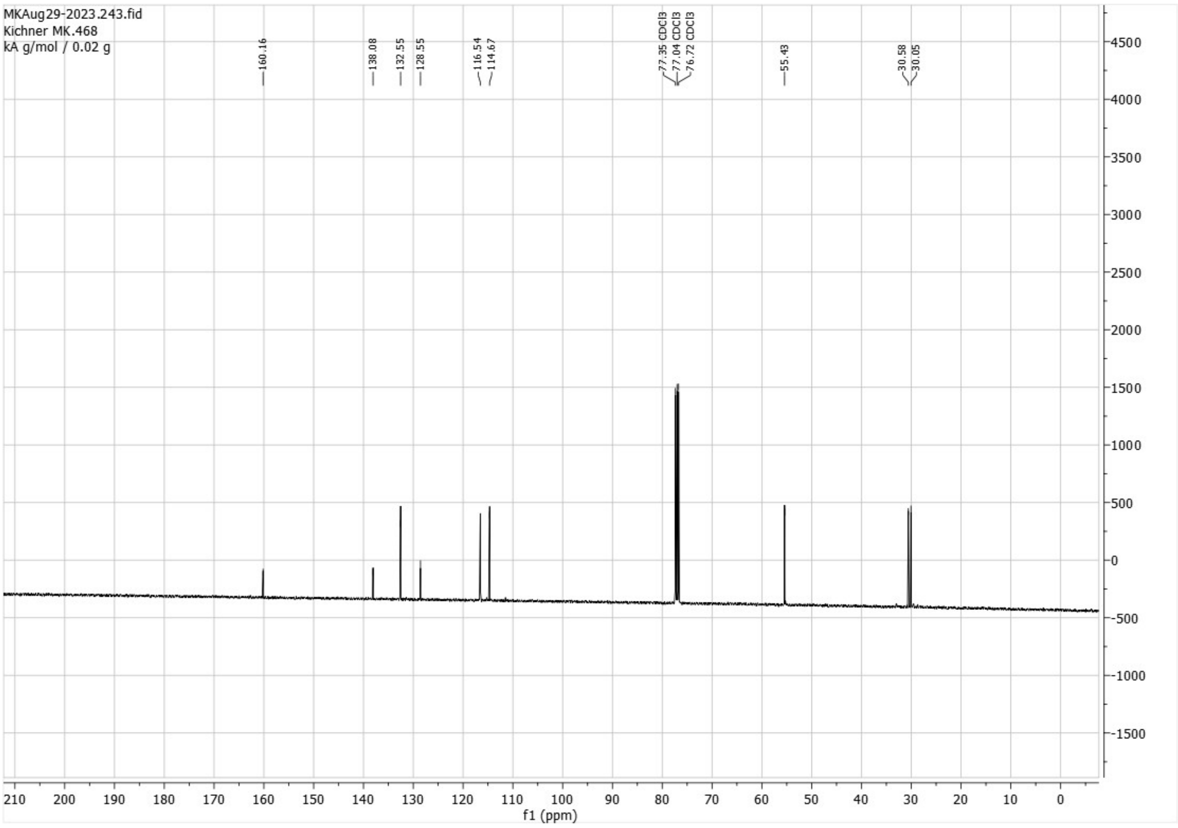
MKAug29-2023.243.fid
Kichner MK.468
kA g/mol / 0.02 g
160.16
138.08
132.55
128.55
116.54
114.67
77.35 CDCl3
77.04 CDCl3
76.72 CDCl3
55.43
30.58
30.05
f1 (ppm)

Compound 9D

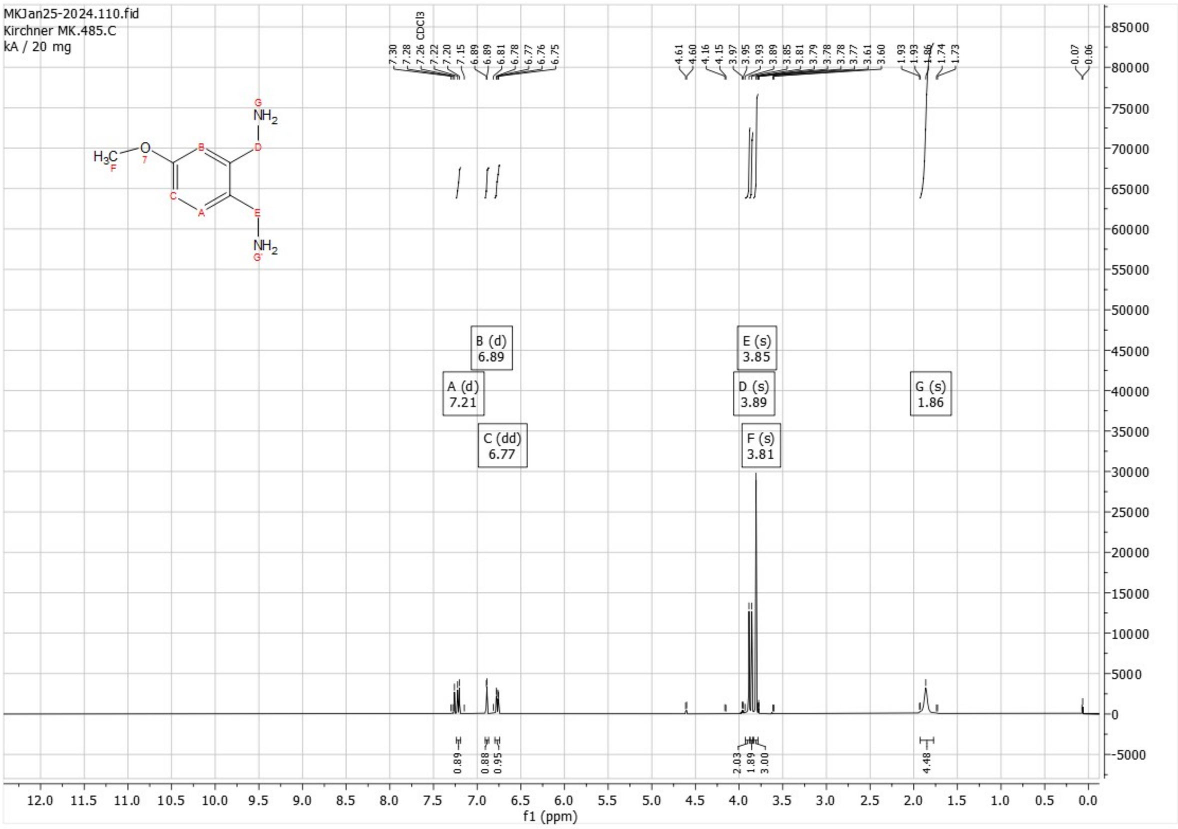
MKJan25-2024.110.fid
Kirchner MK.485.C
kA / 20 mg
A (d)
7.21
B (d)
6.89
C (dd)
6.77
E (s)
3.85
D (s)
3.89
F (s)
3.81
G (s)
1.86
f1 (ppm)

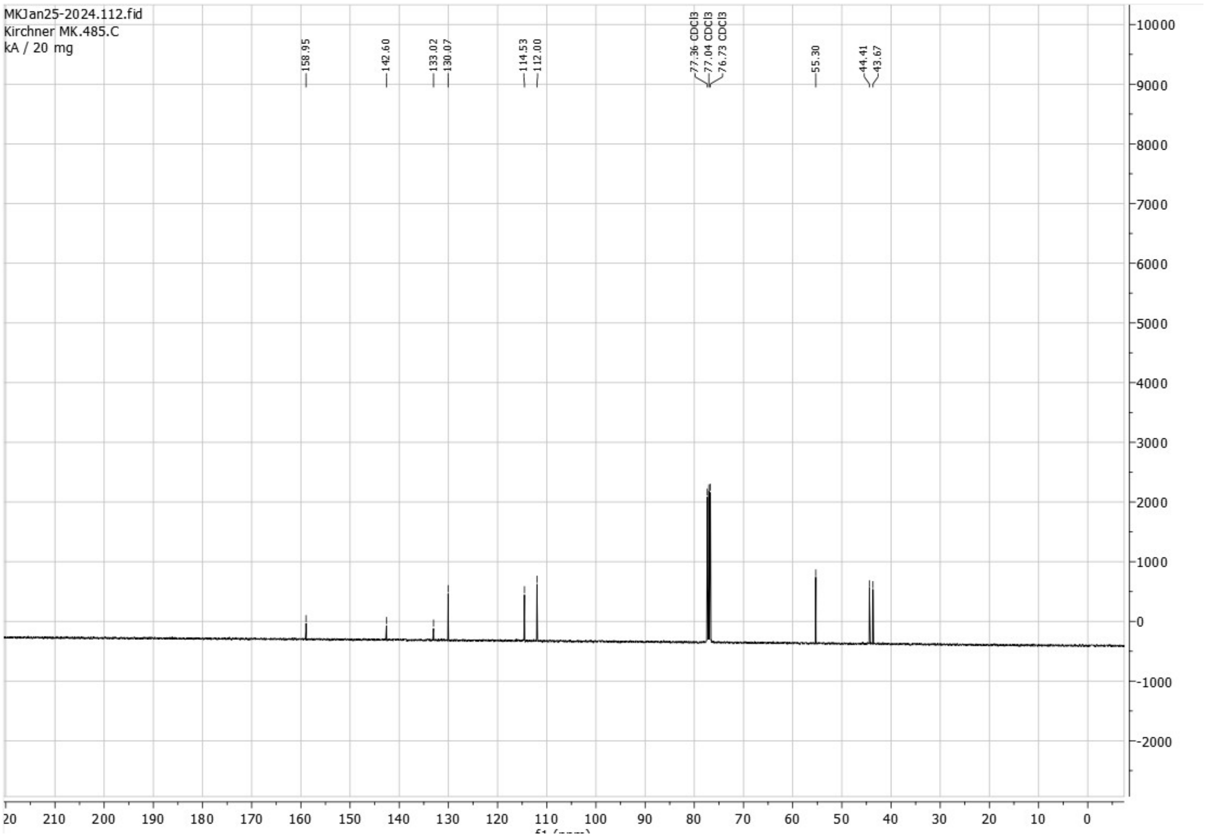
MKJan25-2024.112.fid
Kirchner MK.485.C
kA / 20 mg
158.95
142.60
133.02
130.07
114.53
112.00
77.36 CDCl3
77.04 CDCl3
76.73 CDCl3
55.30
44.41
43.67

Compound 9E

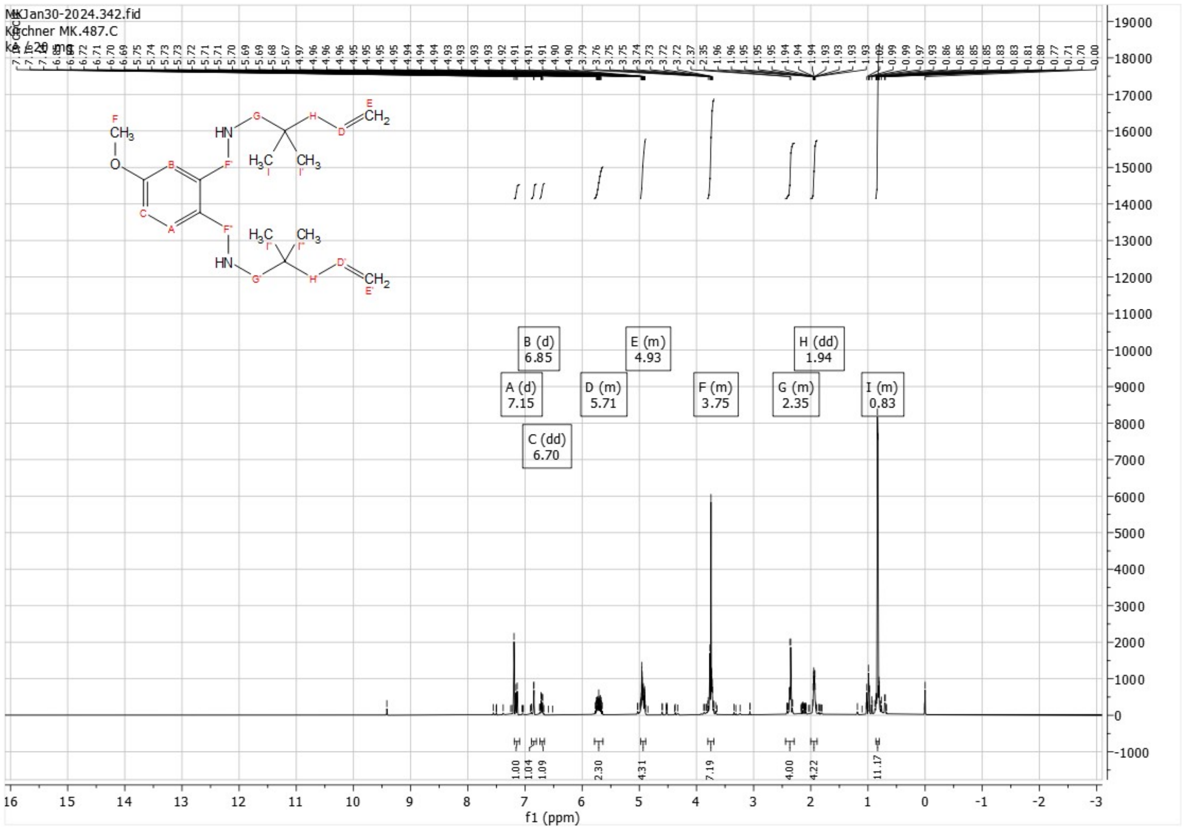
MKJan30-2024.342.fid
Kirchner MK.487.C
A (d) 7.15
B (d) 6.85
C (dd) 6.70
D (m) 5.71
E (m) 4.93
F (m) 3.75
G (m) 2.35
H (dd) 1.94
I (m) 0.83
1.00
1.04
1.09
2.30
4.31
7.19
4.00
4.22
11.17
f1 (ppm)

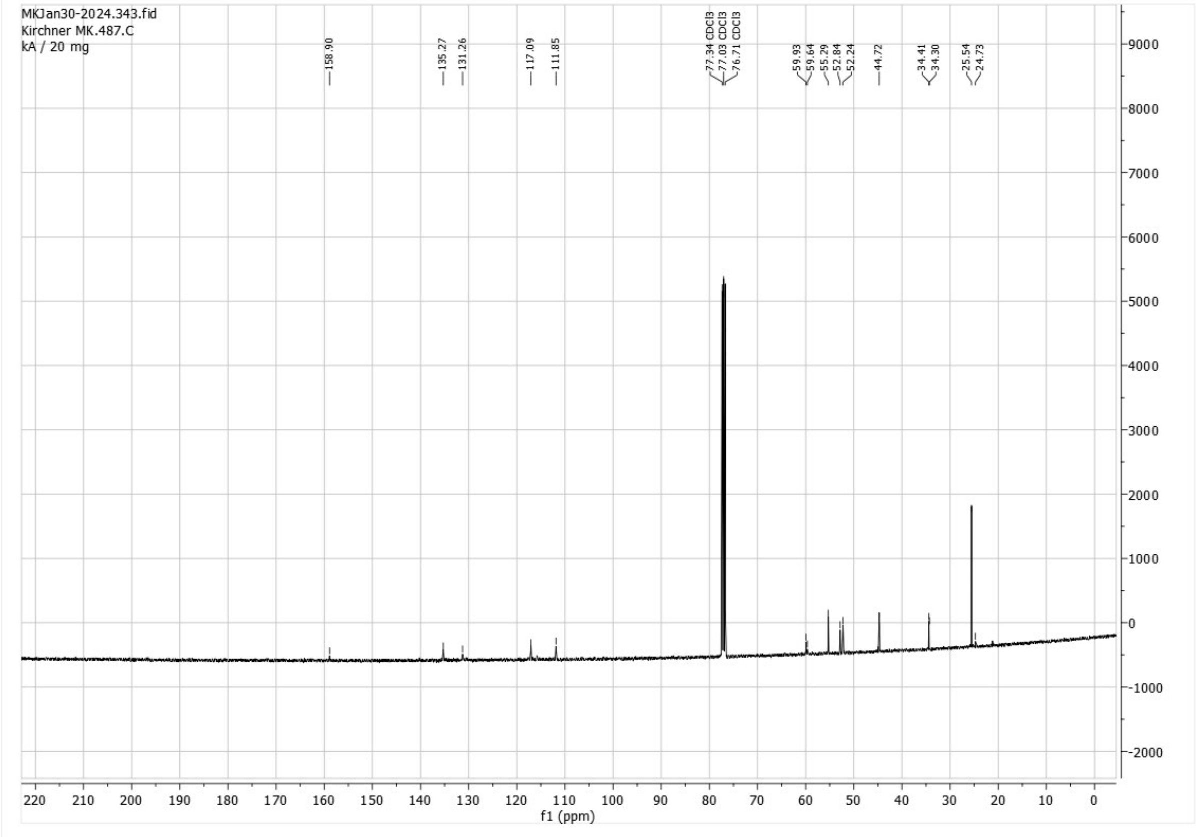
MKJan30-2024.343.fid
Kirchner MK.487.C
kA / 20 mg
f1 (ppm)

Compound 9

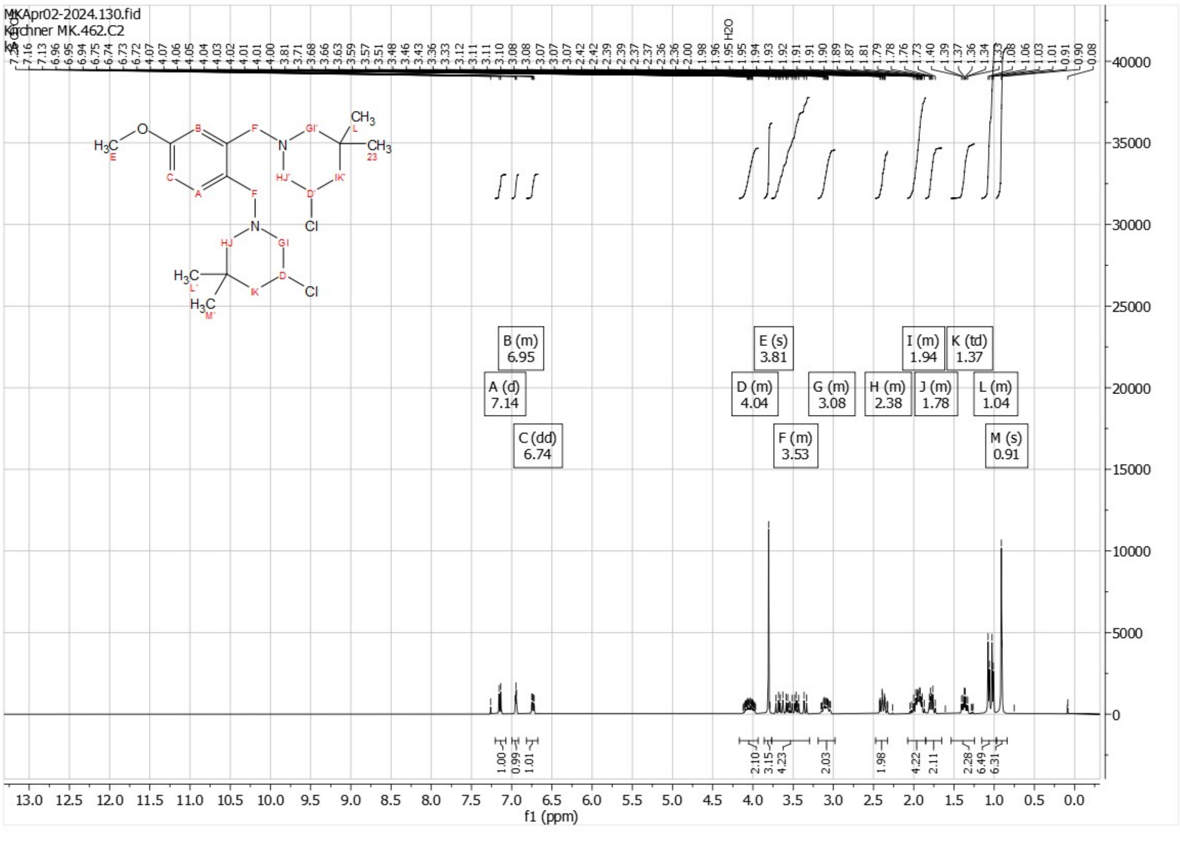
MKApr02-2024.130.fid
Kirchner MK.462.C2
kA
B (m) 6.95
A (d) 7.14
C (dd) 6.74
E (s) 3.81
D (m) 4.04
F (m) 3.53
G (m) 3.08
H (m) 2.38
I (m) 1.94
J (m) 1.78
K (td) 1.37
L (m) 1.04
M (s) 0.91
f1 (ppm)

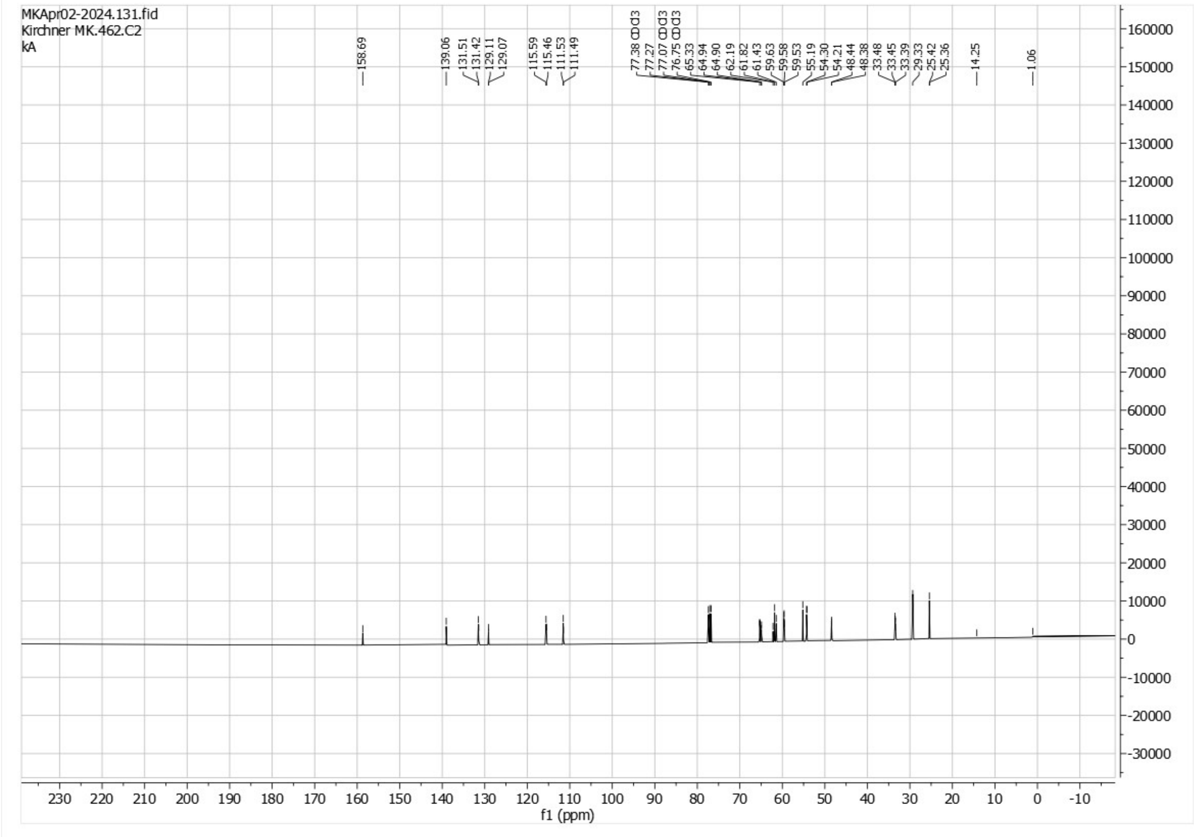

Compound 15A

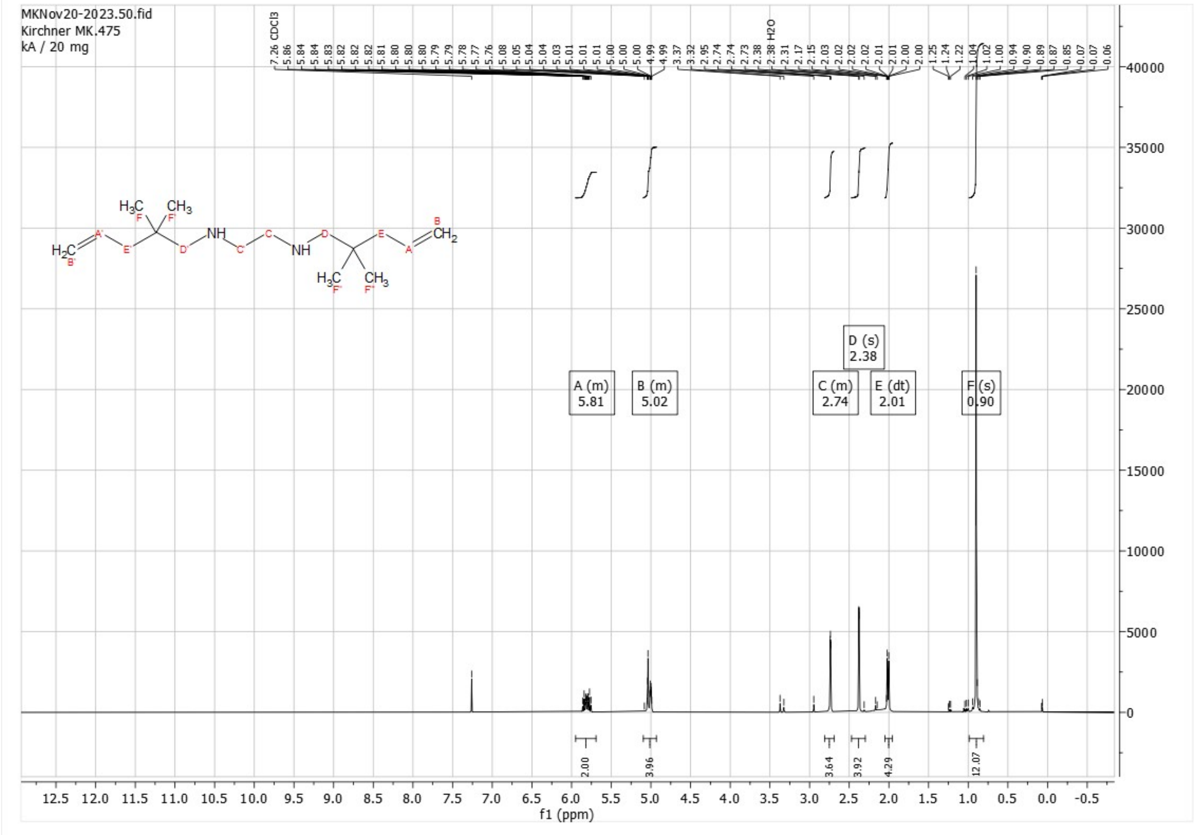

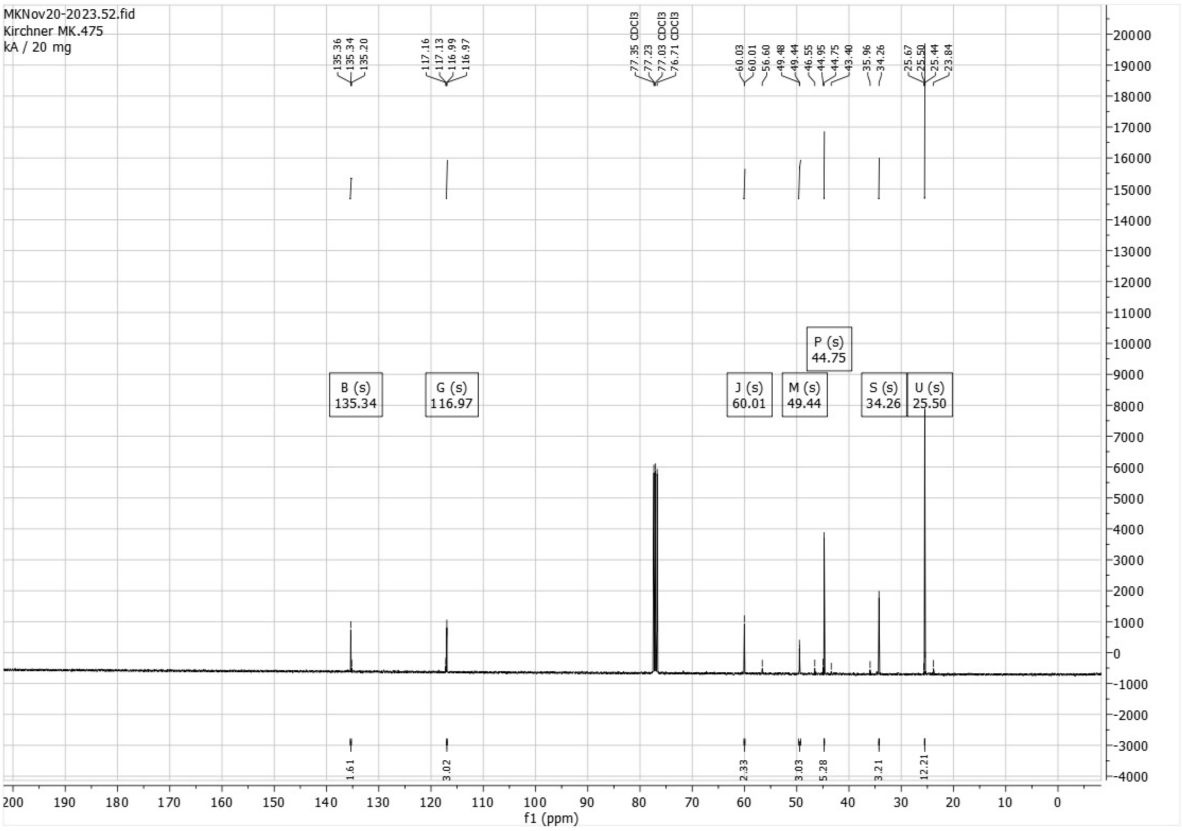
MKNov20-2023.52.fid
Kirchner MK.475
kA / 20 mg
B (s) 135.34
G (s) 116.97
J (s) 60.01
M (s) 49.44
P (s) 44.75
S (s) 34.26
U (s) 25.50
f1 (ppm)

Compound 15

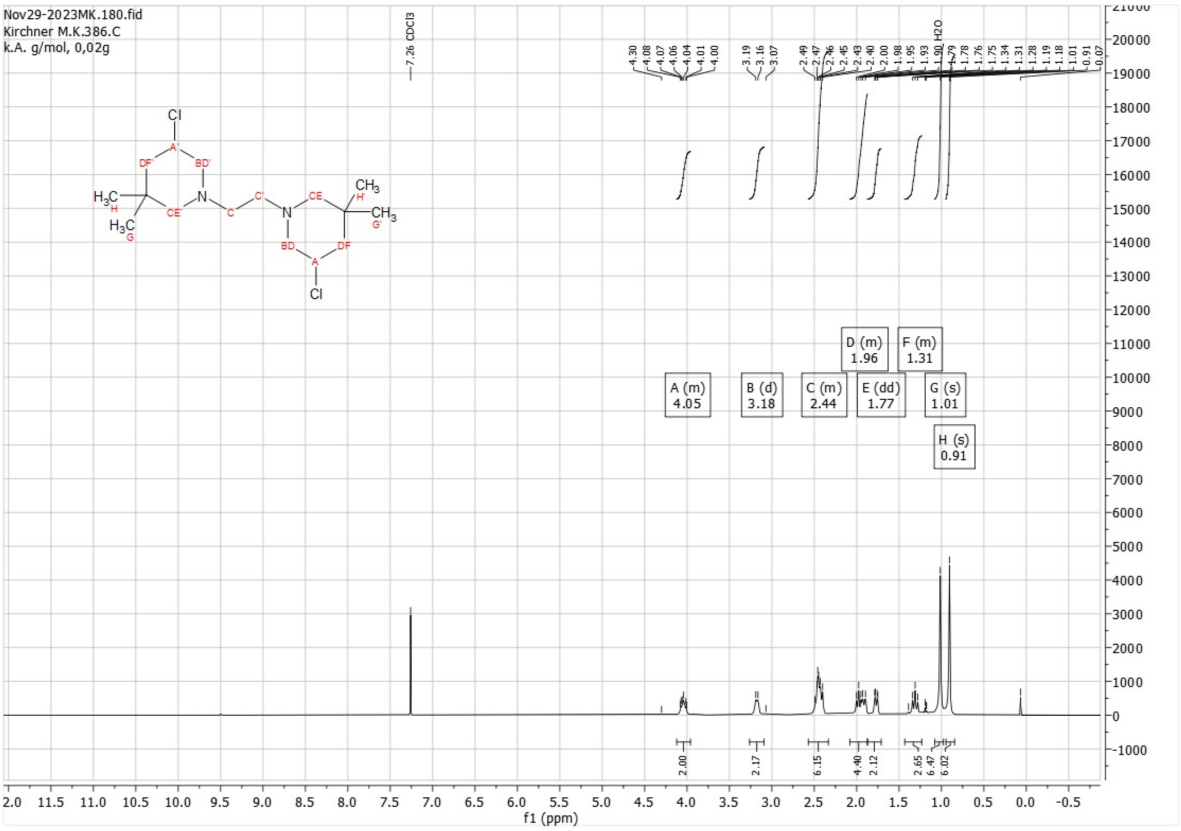
Nov29-2023MK.180.fid
Kirchner M.K.386.C
k.A. g/mol, 0,02g
A (m) 4.05
B (d) 3.18
C (m) 2.44
D (m) 1.96
E (dd) 1.77
F (m) 1.31
G (s) 1.01
H (s) 0.91
f1 (ppm)

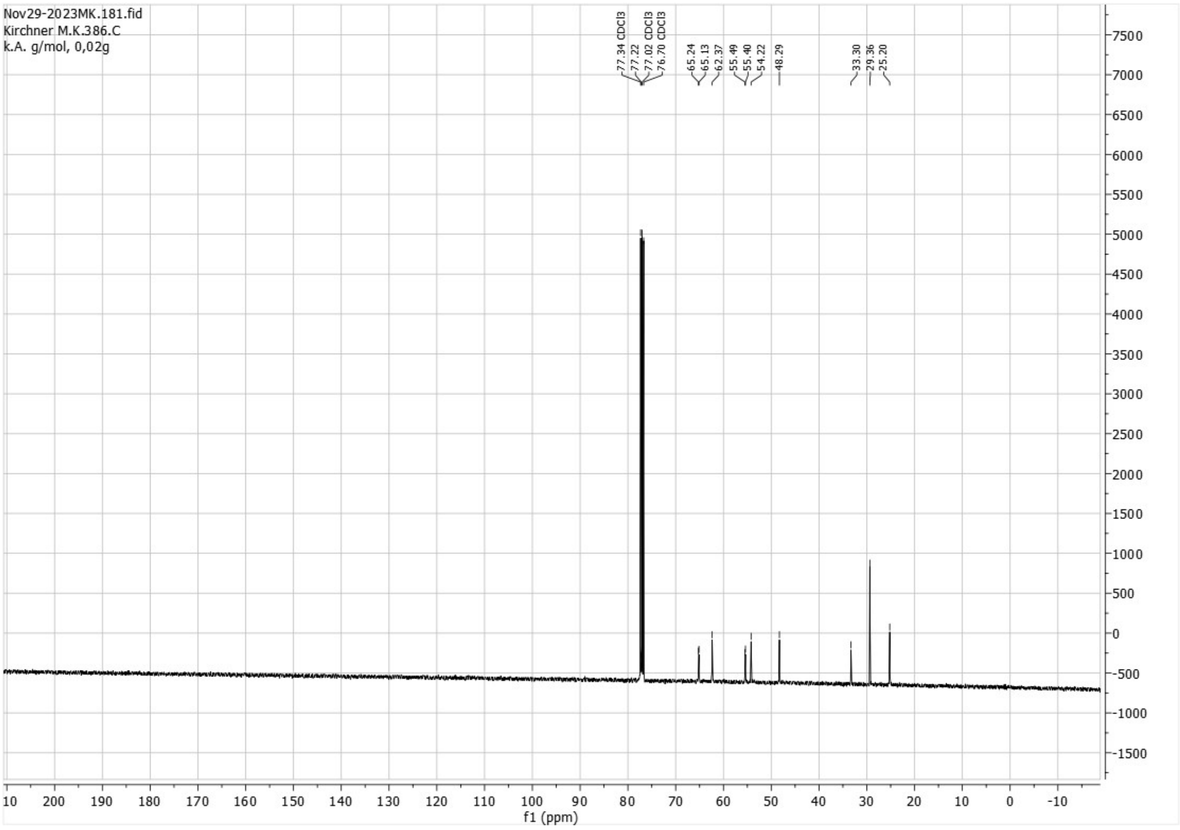
Nov29-2023MK.181.fid
Kirchner M.K.386.C
k.A. g/mol, 0,02g
77.34 CDCl3
77.22 CDCl3
77.02 CDCl3
76.70 CDCl3
65.24
65.13
62.37
55.49
55.40
54.22
48.29
33.30
29.36
25.20
f1 (ppm)

Compound 22

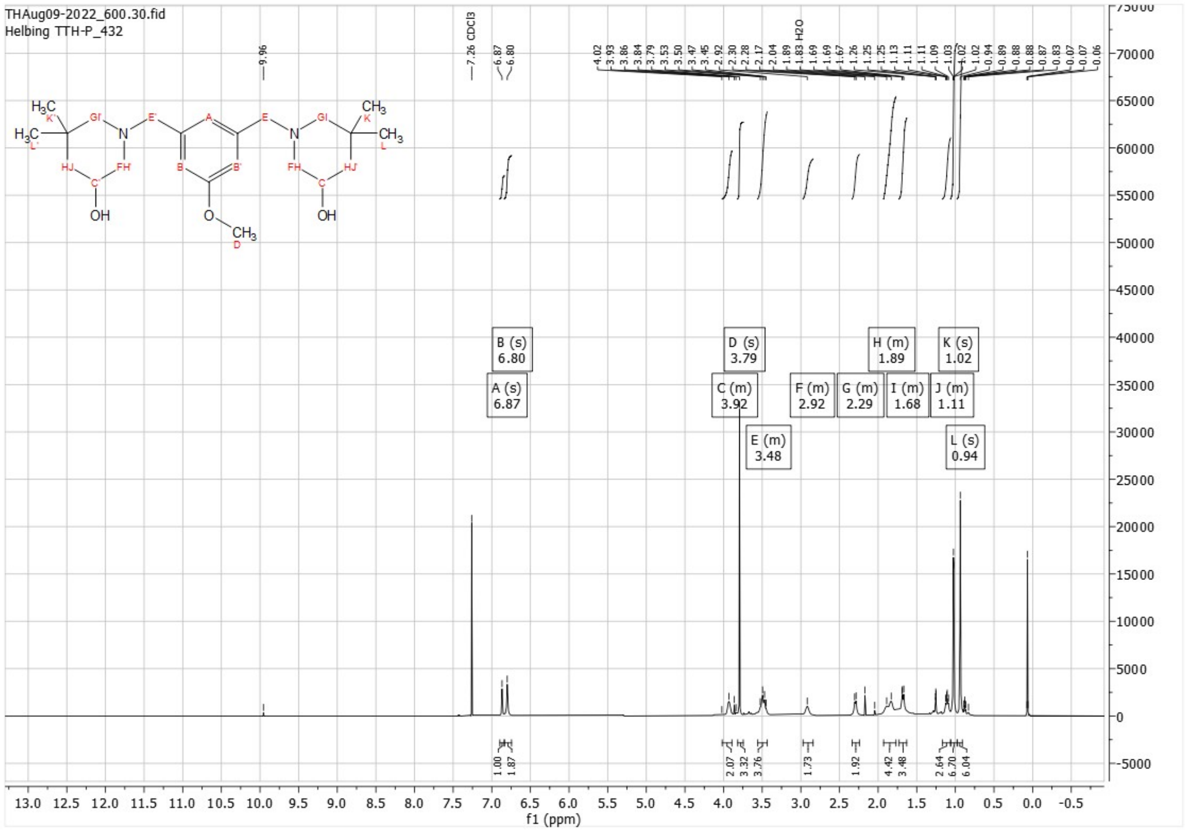
THAug09-2022_600.30.fid
Helbing TTH-P_432
9.96
7.26 CDCl3
6.87
6.80
1.83 H2O
B (s) 6.80
A (s) 6.87
D (s) 3.79
C (m) 3.92
E (m) 3.48
F (m) 2.92
G (m) 2.29
H (m) 1.89
I (m) 1.68
J (m) 1.11
K (s) 1.02
L (s) 0.94
OH
OH
f1 (ppm)

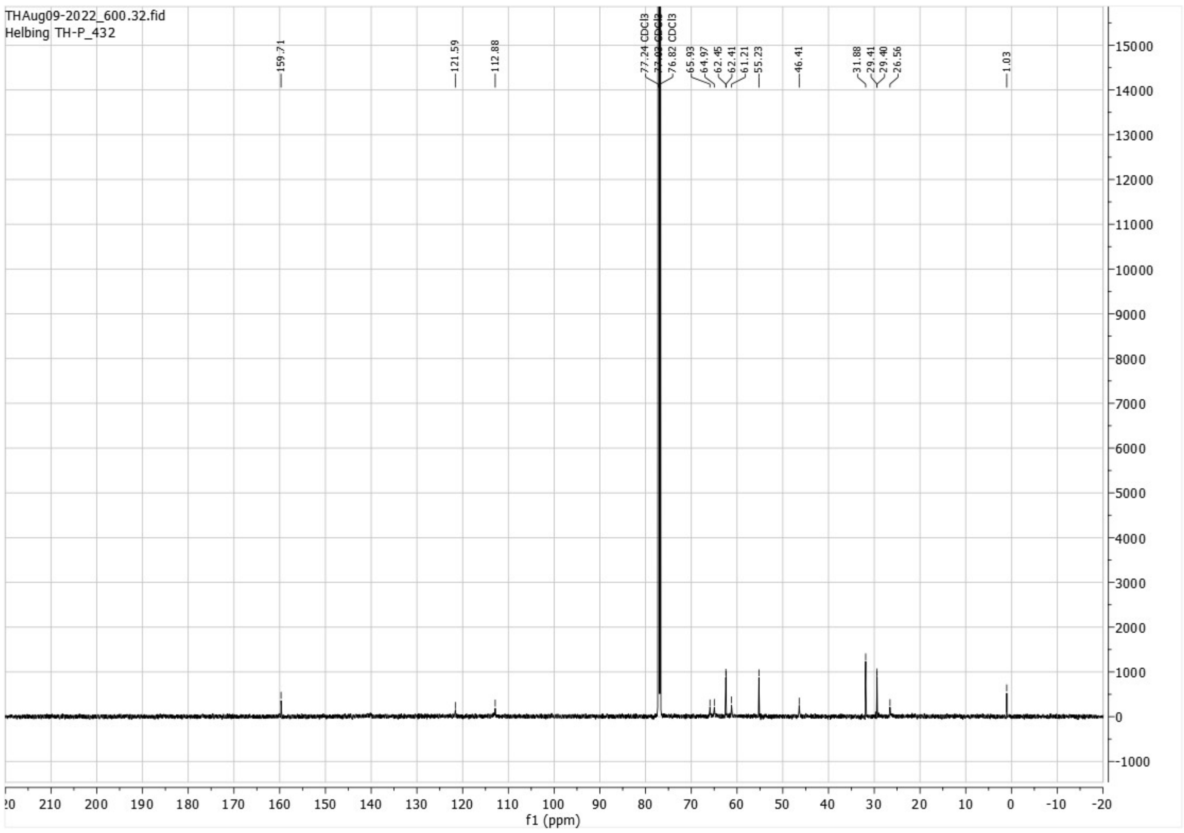
THAug09-2022_600.32.fid
Helbing TH-P_432
159.71
121.59
112.88
77.24 CDCl3
77.03 CDCl3
76.82 CDCl3
65.93
64.97
62.45
62.41
61.21
55.23
46.41
31.88
29.41
29.40
26.56
1.03
f1 (ppm)

5. References

1 I. D. M. Kresna, Z. G. Wuisan, J.-M. Pohl, U. Mettal, V. L. Otoya, M. Gand, M. Marner, L. L. Otoya, N. Böhringer, A. Vilcinskas and T. F. Schäberle, Genome-Mining-Guided Discovery and Characterization of the PKS-NRPS-Hybrid Polyoxyperuin Produced by a Marine-Derived Streptomycete, *Journal of natural products*, 2022, **85**, 888–898.

2 M. Marner, M. A. Patras, M. Kurz, F. Zubeil, F. Förster, S. Schuler, A. Bauer, P. Hammann, A. Vilcinskas, T. F. Schäberle and J. Glaeser, Molecular Networking-Guided Discovery and Characterization of Stechlisins, a Group of Cyclic Lipopeptides from a Pseudomonas sp, *Journal of natural products*, 2020, **83**, 2607–2617.

3 L. Wang, V. Linares-Otoya, Y. Liu, U. Mettal, M. Marner, L. Armas-Mantilla, S. Willbold, T. Kurtán, L. Linares-Otoya and T. F. Schäberle, Discovery and Biosynthesis of Antimicrobial Phenethylamine Alkaloids from the Marine Flavobacterium Tenacibaculum discolor sv11, *Journal of natural products*, 2022, **85**, 1039–1051.

4 T. Stiernagle, Maintenance of C. elegans, *WormBook : the online review of C. elegans biology*, 2006, 1–11.

5 C. G. Grevelding, The female-specific W1 sequence of the Puerto Rican strain of Schistosoma mansoni occurs in both genders of a Liberian strain, *Molecular and biochemical parasitology*, 1995, **71**, 269–272.

6 R. GONNERT, Schistosomiasis-Studien. II. Uber die Eibildung bei Schistosoma mansoni und das Schicksal der Eier im Wirtsorganismus, *Zeitschrift fur Tropenmedizin und Parasitologie*, 1955, **6**, 33–52.

7 C. D. Dettman, S. B. Higgins-Opitz and A. Saikoolal, Enhanced efficacy of the paddling method for schistosome infection of rodents by a four-step pre-soaking procedure, *Parasitology research*, 1989, **76**, 183–184.

8 J. Kellershohn, L. Thomas, S. R. Hahnel, A. Grünweller, R. K. Hartmann, M. Hardt, A. Vilcinskas, C. G. Grevelding and S. Haeberlein, Insects in anthelminthics research: Lady beetle-derived harmonine affects survival, reproduction and stem cell proliferation of Schistosoma mansoni, *PLoS neglected tropical diseases*, 2019, **13**, e0007240.

9 I. Zuravka, R. Roesmann, A. Sosic, R. Göttlich and B. Gatto, Bis-3-chloropiperidines containing bridging lysine linkers: Influence of side chain structure on DNA alkylating activity, *Bioorganic & medicinal chemistry*, 2015, **23**, 1241–1250.

10 I. Zuravka, R. Roesmann, A. Sosic, W. Wende, A. Pingoud, B. Gatto and R. Göttlich, Synthesis and DNA cleavage activity of Bis-3-chloropiperidines as alkylating agents, *ChemMedChem*, 2014, **9**, 2178–2185.

11 I. Zuravka, A. Sosic, B. Gatto and R. Göttlich, Synthesis and evaluation of a bis-3-chloropiperidine derivative incorporating an anthraquinone pharmacophore, *Bioorganic & medicinal chemistry letters*, 2015, **25**, 4606–4609.

12 T. Helbing, C. Carraro, A. Francke, A. Sosic, M. de Franco, V. Gandin, R. Göttlich and B. Gatto, Aromatic Linkers Unleash the Antiproliferative Potential of 3-Chloropiperidines Against Pancreatic Cancer Cells, *ChemMedChem*, 2020, **15**, 2040–2051.

13 T. Helbing, M. Georg, F. Stöhr, C. Carraro, J. Becker, B. Gatto and R. Göttlich, Understanding the Alkylation Mechanism of 3-Chloropiperidines – NMR Kinetic Studies and Isolation of Bicyclic Aziridinium Ions, *Eur J Org Chem*, 2021, **2021**, 5905–5913.

14 T. Helbing, M. Kirchner, J. Becker and R. Göttlich, Separation of the Thorpe−Ingold and Reactive Rotamer Effect by Using the Formation of Bicyclic Aziridinium Ions, *Eur J Org Chem*, 2022, **2022**.

6. Author contribution

M. Kirchner performed the synthesis and analytics and prepared the draft.

M. Marner performed the biological studies and prepared the draft.

T. Kramer assisted with the synthesis and analytics.

S. Haeberlein performed the parasitological studies.

F. Mühlemeyer assisted with the biological studies.

M. Oberpaul assisted with the biological studies.

J. Eichberg assisted with the biological studies.

R. Göttlich assisted in preparing the draft and administered the project.

All authors have reviewed the manuscript.
